# Highly Thermally Stable and Gas Selective Hexaphenylbenzene Tröger’s Base Microporous Polymers

**DOI:** 10.1021/acsami.4c15333

**Published:** 2024-12-03

**Authors:** Yue Wu, Ariana R. Antonangelo, C. Grazia Bezzu, Mariolino Carta

**Affiliations:** Department of Chemistry, Faculty of Science and Engineering, Swansea University, Grove Building, Singleton Park, Swansea SA2 8PP, U.K.

**Keywords:** hexaphenylbenzene, Tröger’s base, PIMs, flame retardants, gas adsorption, carbon capture

## Abstract

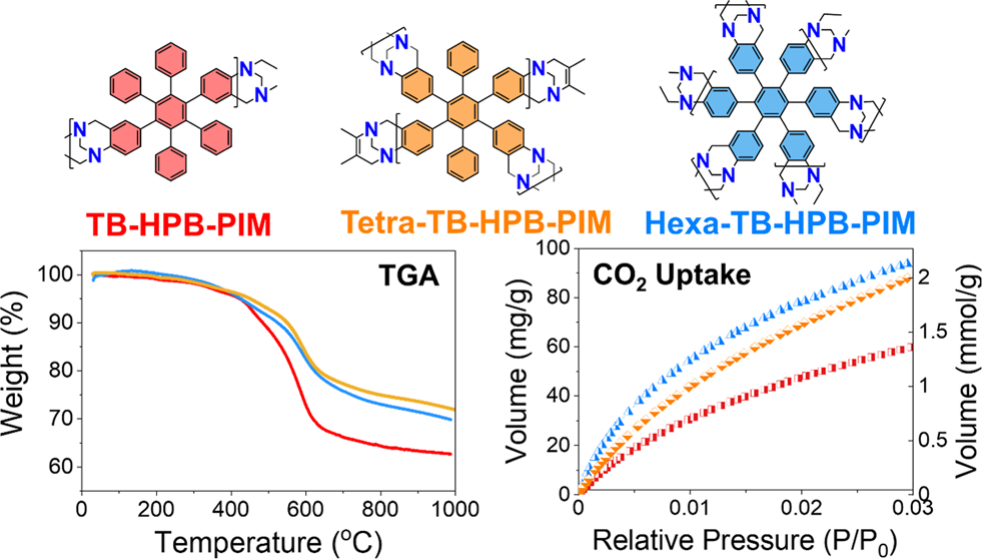

This study shows the multistep synthesis of a series of Tröger’s base polymers of intrinsic microporosity (TB-PIMs) based on a hexaphenylbenzene (HPB) core, with a focus on evaluating their thermal stability, porosity, and CO_2_ capture performance. Both ladder and linear structures were prepared, designed to feature tunable nitrogen content and porosity. Our findings demonstrate that polymers with higher nitrogen content, such as tetra-TB-HPB, exhibit superior CO_2_ affinity and selectivity, attributed to enhanced interactions with CO_2_ and optimized micropore sizes. Linear TB-polymers 1 and 2 are also made for comparison and show competitive performance in carbon capture, suggesting that cost-effective, simpler-to-synthesize materials can achieve efficient gas separation. The study reveals that increased porosity significantly enhances CO_2_ capacity and selectivity, particularly in networked TB-HPB-PIMs with high surface areas and narrow micropores, achieving values up to 544 m^2^ g^–1^, CO_2_ uptake of 2.00 mmol g^–1^, and CO_2_/N_2_ selectivity of 45.6. The thermal properties of these materials, assessed via thermogravimetric analysis (TGA), show that TB-HPB-PIMs maintain robust thermal stability in nitrogen atmosphere, with tetra- and hexa-TB-HPBs leading the series. However, in oxidative environments, denser polymers such as TB-HPB and linear TB-polymer 1 demonstrate higher performance, likely due to restricted air diffusion. Overall, our findings highlight the critical need to balance porosity and thermal stability in TB-HPB-PIMs for applications in gas separation, carbon capture, and the potential for these polymers as flame retardant materials. Tetra-TB-HPB stands out as the most promising material for CO_2_ capture and thermal stability under inert conditions, while denser polymers like TB-HPB offer superior performance in oxidative environments.

## Introduction

**Figure 1:**
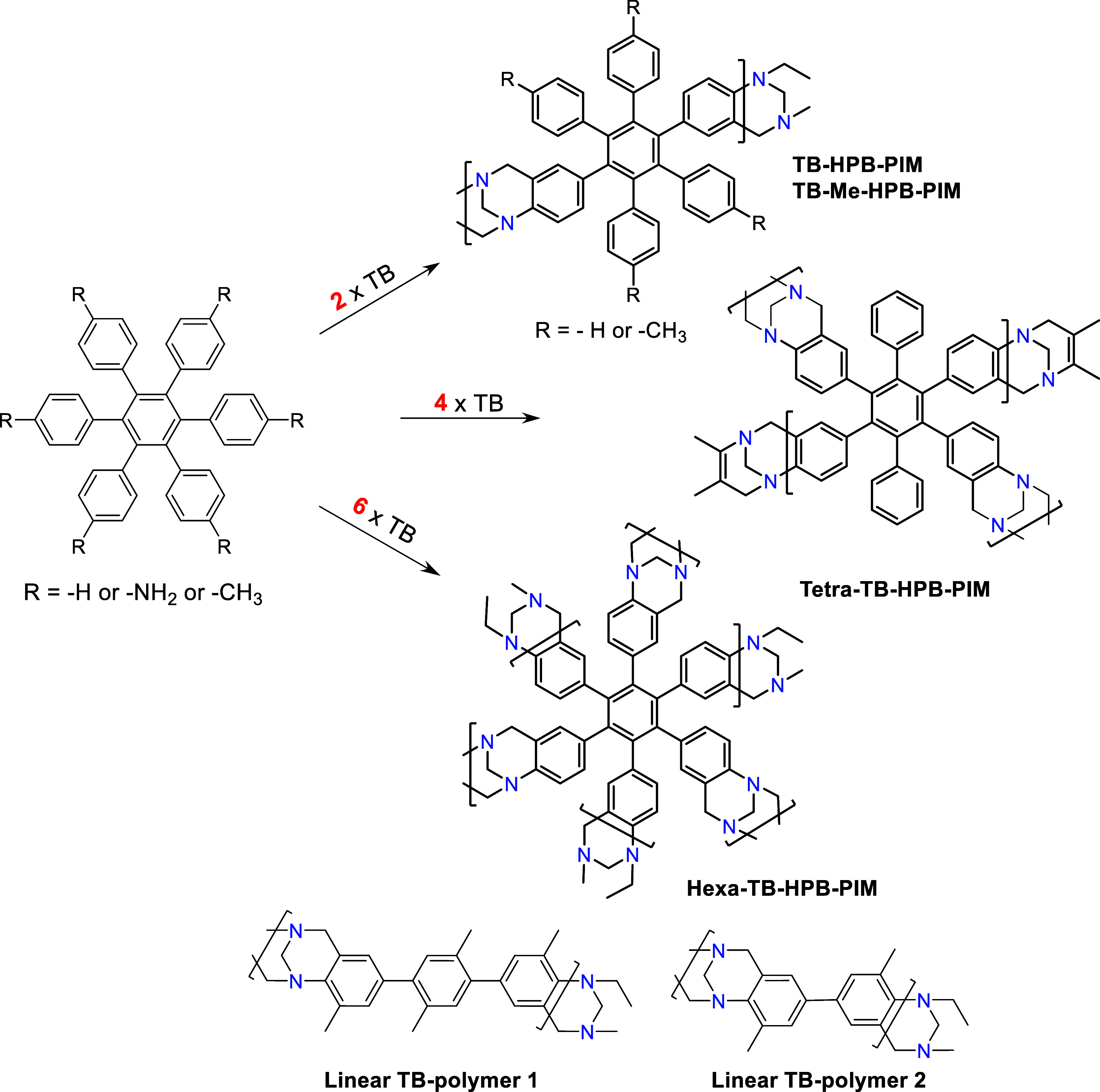
Preparation of different TB-HPB-PIMs. Reagents and conditions: dimethoxy methane (DMM), trifluoroacetic acid (TFA) and dichloromethane (DCM).

As advancements in technology and material chemistry unfold, the unavoidable release of high volumes of gases into the atmosphere becomes increasingly concerning, as it significantly contributes to global warming.^1^ Carbon dioxide, being the predominant greenhouse gas emitted from power plants, is considered as the prime suspect, and since the last ice age its concentration in the atmosphere has increased at a rate approximately 250 times faster than that of natural sources. Substantial scientific evidence overwhelmingly attributes this escalation to human activities, underscoring our responsibility to actively seek and implement effective solutions for its mitigation.^2,3^ In this context, carbon capture and storage (CCS) methodologies are presently implemented on a global scale, showing great potential to mitigate CO_2_ emissions arising from industrial processes. The International Energy Agency (IEA), the International Renewable Energy Agency (IRENA), and the Intergovernmental Panel on Climate Change (IPCC) have all recognized the crucial role of CCS in meeting global climate targets.^4^ Two primary pathways exist for the implementation of CCS: one involves extracting CO_2_ from power plant emissions before its release into the atmosphere, while the other involves removing it from the environment through what is known as direct air capture.^5,6^ While the latter is a relatively new and attracting method, the former is considerably more advanced and, so far, various established options have been reported, such as adsorption, absorption, membranes, and hydrate-based separations.^7−9^ These strategies are commonly linked to the so-called postcombustion carbon capture from flue gas, which refers to the exhaust gas generated from burning fossil fuels and typically consists of a mixture of ∼15% CO_2_ and ∼80% N_2_, along with other minor components such as H_2_O, CO, NO_*x*_, and SO_*x*_.^10,11^ Porous materials are considered ideal for CCS, due to their typically high surface areas, high thermal and mechanical stability and selective gas adsorption, which can be tuned for specific gases using appropriate functional groups.^12^ Among them, the crystalline metal–organic frameworks (MOFs) and zeolites are well-known for their high uptake capacity, tunable surface chemistry, and low energy requirements for regeneration, making them efficient for CO_2_ capture.^13^ Also amorphous materials and polymers proved to be very efficient for carbon capture. Their flexible composition, improved compatibility with other amorphous materials that often are at their interface (especially with membranes),^14^ and ease of synthesis compared to many MOFs, make them attractive candidates.^15^ Polymers of intrinsic microporosity (PIMs) constitute a recently established category of functional amorphous materials.^16^ Their porosity derives from the careful selection of rigid and contorted building blocks (monomers), that aim to prevent an efficient packing of polymer chains in the solid state, which leads to the development of micropores.^17^ Over the past few years, PIMs have proved to be very efficient materials in various applications, including gas separation membranes,^18,19^ gas storage,^20^ electrode coating for electrochemical reactions,^21^ water purification,^22,23^ and catalysis.^24^ PIMs showed remarkable efficacy in the selective separation of carbon dioxide from other gases (i.e., CO_2_ from N_2_ in flue gas carbon capture applications),^25^ establishing themselves as the new state-of-the-art for CO_2_-involved gas separation membranes.^26^ Their outstanding performance is attributed to the combination of a narrow pore size range (typically 3.5–8.5 Å),^27^ which facilitates excellent molecular sieving behavior (CO_2_ has a kinetic diameter of 3.3 Å vs N_2_ 3.64 Å), and an entirely organic backbone that enhances CO_2_ solubility in its backbone.^26,28−30^ However, the main advantage of PIMs probably lies in the extensive range of functional groups that can be incorporated into their monomers, either before polymerization^31^ or introduced into the preformed backbone through postpolymerization methods.^25,32^ This feature allows for precise tuning of their properties, constantly broadening the scope of their applications.^33^ A specific example of this facile functionalization is represented by a recent subclass of PIMs, known as Tröger’s base PIMs (TB-PIMs).^28,34^ The TB core combines a high rigid and contorted structure, typical of highly porous PIMs, with the presence of two bridgehead basic nitrogen atoms that allow for a better capture of CO_2_ due to its mild Lewis acidity. This unique combination makes TB-PIMs ideal for applications such as CO_2_ separation and heterogeneous catalysis.^24,34^ In the synthesis of the bicyclic Tröger’s base, a condensation reaction between an aniline and a methylene precursor (i.e., formaldehyde or dimethoxymethane), under acidic conditions is employed (Figure 1). Consequently, the preparation of a polymer necessitates the presence of amino moieties. If the number of these amino-functionalities is exactly two per monomer, the polymer can be soluble (ladder) and can be used as the main component of membranes for gas separation.^28,34,35^ If the average functionality is over two, the polymer will be highly networked, and so completely insoluble, and can be used for applications such as heterogeneous catalysis and as solid adsorbent materials.^24,36^

Looking at specific molecules that can be used to make microporous materials, hexaphenylbenzenes (HPBs) are of particular interest, as free rotation around each aromatic ring is hindered by the neighbor’s electronic repulsion.^37^ The enhanced rigidity of these monomers makes them perfect for the preparation of novel PIMs. In the past few years, we exploited the HPB core to prepare novel PIMs via the characteristic benzodioxin polymerization, producing polymeric membranes that proved efficient and selective for gas separation applications.^38^ In particular, we demonstrated that we could tailor the synthesis to decorate the HPB core with different substituents, tuning in this way the selectivity for several gas pairs. In addition to their high porosity and facile functionalization, all PIMs, and especially HPB-PIMs, exhibited a remarkable thermal stability.^39^ To the best of our knowledge, no studies have been conducted to systematically assess and improve the thermal properties of PIMs, and this becomes very relevant if these polymers are studied as flame-retardants materials. Research shows that there is a correlation between the ratio of C and N in flame retardant materials, and the lower the C/N ratio better is the performance.^40,41^

In this paper we report a series of PIMs based on HPB and TB cores with varying carbon to nitrogen ratios (C/N) and pore structures were designed and synthesized by altering the type and number of functional groups so that we can improve simultaneously the selectivity of CO_2_ toward N_2_ (aiming at carbon capture applications) and their inherent thermal stability (aiming at potential flame-retardant materials). Additionally, two “linear” TB polymers with increased flexibility due to higher amounts of free rotation sites around the main backbone were prepared, to better understand the impact of porosity on gas capture and thermal stability. It is known that greater flexibility in molecular chains often leads to denser packing and, consequently, decreased porosity.^42,43^

## Materials and Methods

Commercially available reagents and gases were used without further purification. All reactions using air/moisture sensitive reagents were performed in oven-dried or flame-dried apparatus, under a nitrogen atmosphere. TLC analysis refers to analytical thin layer chromatography, using aluminum-backed plates coated with Merck Kieselgel 60 GF254. Product spots were viewed either by the quenching of UV fluorescence, and in some cases by staining with permanganate stain [preparation: potassium permanganate (3 g) + potassium carbonate (20 g) + 5% aqueous NaOH (5 mL) + water (300 mL)]. Melting points were recorded using a Cole-Parmer Stuart Digital Melting Point Apparatus and are uncorrected. Infrared spectra were recorded using a PerkinElmer Spectrum Two FT-IR spectrometer. LRMS were measured using the Advion Interchim Scientific expression compact mass spectrometer. ^1^H NMR spectra were recorded in deuterated solvent, as stated, using an Avance Bruker DPX 500 (500 MHz) instruments, with ^13^C NMR spectra recorded at 126 MHz. Solid-state ^13^C NMR spectra were recorded using a Bruker Avance III spectrometer equipped with a wide-bore 9.4 T magnet (Larmor frequencies of 100.9 MHz for ^13^C). Samples were packed into standard zirconia rotors with 4 mm outer diameter and rotated at a magic angle spinning (MAS) rate of 12.5 kHz. Spectra were recorded with cross-polarization (CP) from ^1^H using a contact pulse (ramped for ^1^H) of 1.5 ms. High-power (ν1 ≈ 100 kHz) TPPM-15 decoupling of ^1^H was applied during acquisition to improve resolution. Signal averaging was carried out for 6144 transients with a recycle interval of 2 s. Chemical shifts are reported in ppm relative to (CH_3_)_4_ Si (TMS) using the CH_3_ signal of l-alanine (δ = 20.5 ppm) as a secondary solid reference. Low-temperature N_2_ (77 and 298 K) and CO_2_ (195, 273 and 298 K) adsorption/desorption measurements of polymer powders were made using a Anton Paar Nova 600. Samples were degassed over 8 h at 80 °C under high vacuum prior to analysis. The gases were supplied by BOC and used without any further purification (N_2_ purity >99.999, CO_2_ purity >99.995%, air: 21% ± 0.5% oxygen, balance nitrogen). The specimen was measured twice after outgas in two different stations to minimize the error, providing the same results. The data were analyzed with the software provided with the instrument. The BET surface area was calculated at a relative pressure *P*/*P*_0_ < 0.1. NLDFT analysis was performed to calculate the pore size distribution and volume, considering a carbon equilibrium transition kernel at 273 K based on a slit-pore model; the kernel is based on a common, one center, Lennard–Jones model. Heats of adsorption were calculated from the CO_2_ curves measured at 273 and 298 K. The data were analyzed with the Anton Paar Kaomi software and fitted with the Langmuir–Freundlich equation and calculated via the Clausius–Clapeyron equation. TGAs were performed using the PerkinElmer Thermal Analyzer STA 6000 at a heating rate of 10 °C/min from 30 to 995 °C. SEM images were recorded with a Hitachi S-4800 field emission (∼1 nm resolution).

### Ideal Adsorption Solution Theory Selectivity Calculation

The ideal adsorption solution theory (IAST) of Myers and Prausnitz^1^ is typically used to calculate the selectivity of binary mixtures of gases from the single isotherms. The isotherms were fitted with Dual-Site Langmuir–Freundlich using the software IAST++^2^ and the selectivity (*S*) was calculated according to the formula
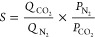
Were *P*_CO_2__ is the partial pressure of CO_2_; *P*_N_2__ is the partial pressure of N_2_; *Q*_N_2__ is the N_2_ uptake; *Q*_CO_2__ is the CO_2_ uptake.

## Results and Discussion

The general procedure for preparing the various HPB-PIMs is outlined in Figure 1. All precursors were obtained using similar methods, which are discussed in the following section. The final bis-, tetra-, and hexa-amino HPB monomers were synthesized through distinct methods and subsequently polymerized under typical TB conditions.^44^

### Synthesis of Diamino-HPB Monomers

**Figure 2:**
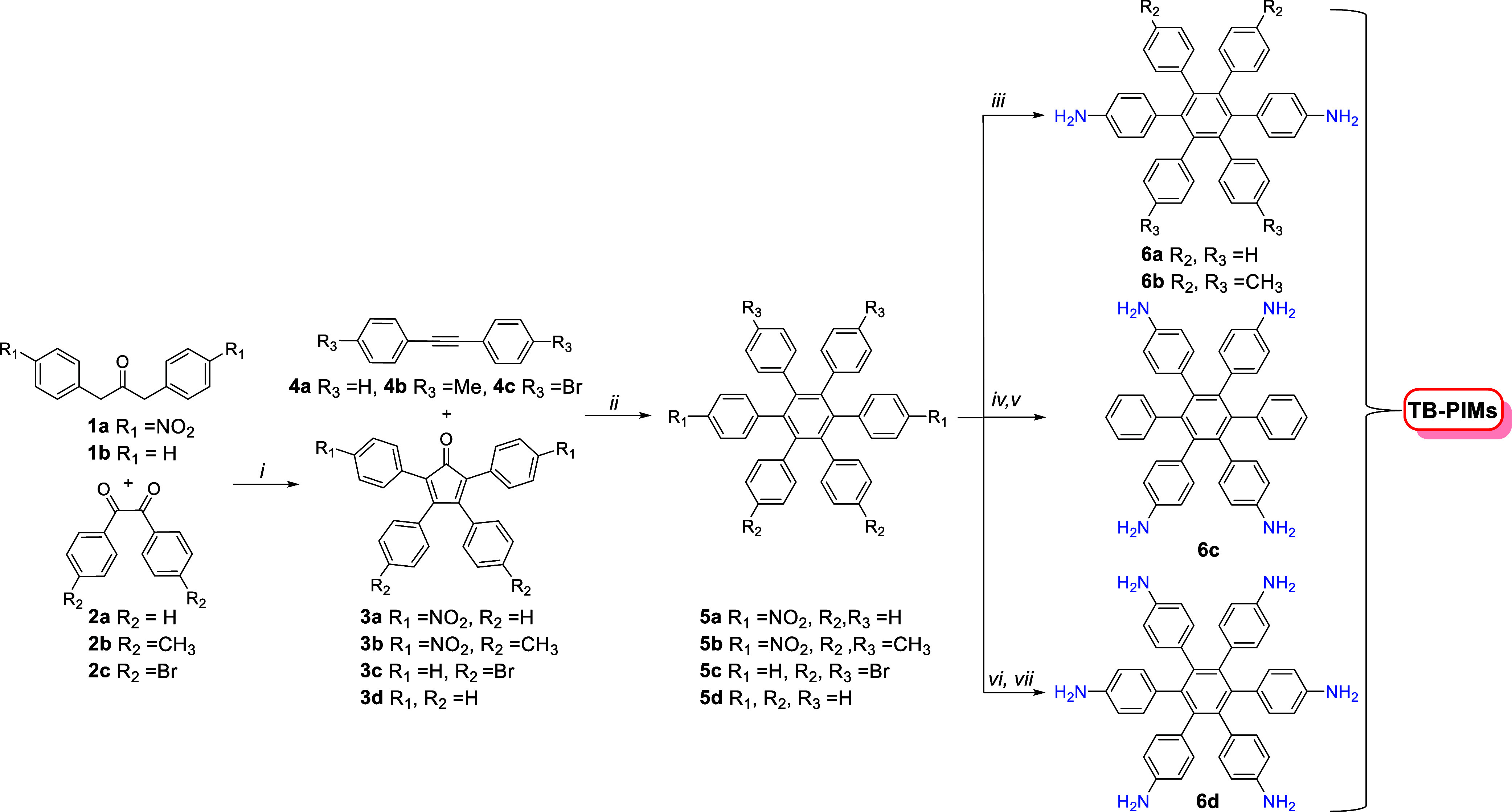
Synthesis of different HPB monomers. Reagents and conditions: (i) KOH, EtOH; (ii) 250 °C in a hydrothermal reactor; (iii) Ni Raney, hydrazine monohydrate, THF; (iv) benzophenone imine, Pd(dba)_3_ and rac-BINAP, sodium *tert*-butoxide, toluene; (v) 2 M HCl; (vi) fuming HNO_3_ and H_2_SO_4_; (vii) Ni Raney, hydrazine monohydrate, DMF.

The synthesis of the ladder-type HPB monomers was achieved using a well-established route involving the Diels–Alder reaction between diphenylacetylene and cyclopentadienone derivatives (CPDs, Figure 2). The diphenylacetylene compounds are obtained through a Sonogashira reaction, while the CPDs are prepared via the reaction of diphenylacetone with a benzil reagent. This versatile synthesis method not only yields relatively high amounts of the desired products but also allows precise control over the positioning of substituents in the final monomers. For example, in our previous work on HPB-catechols, we observed that high molecular weight HPB benzodioxin polymers could be achieved when polymerization sites were positioned as far apart as possible (i.e., in a pseudopara position relative to the central benzene ring). Consequently, we applied this method to synthesize pseudopara bis-aniline HPBs (Figures 2 and 6a–b).

The precursor **1a**, made from 4-nitrophenyl acetic acid, is the key compound for the next steps as it allows the introduction of substituents in “para” position to the HPB core. CPDs **3a** and **3b** are prepared via double aldol condensation via refluxing the 4-nitrophenyl acetic acid and the correspondent benzil **2a** and **2b** in a solution of KOH in ethanol for 1 h.^39^ The dinitro HPBs were obtained in the third step via a Diels–Alder reaction. **3a**–**b** and **4a**–**b** were mixed in a hydrothermal synthesis reactor and heated in the oven at 250 °C, to produce respectively **5a** and **5b** in good yields. Both diamino-HPB monomers were obtained after the reduction of the correspondent nitro-HPB precursors with hydrazine catalyzed by Raney Nickel under nitrogen atmosphere, following a published procedure^45^ and giving **6a** and **6b** in good yields.

### Synthesis of Tetra-amino-HPB Monomer

The two monomers described in the previous section were designed with the goal of producing ladder-type polymers that are expected to be soluble in common organic solvents. However, increasing the nitrogen content can be advantageous for both thermal stability and gas selectivity, as higher nitrogen amounts can enhance decomposition temperatures and influence interactions with polar gases. Consequently, we also aimed to synthesize polymers with more than two amino polymerization sites, such as the tetra-amino **6c** and hexa-amino HPB **6d** shown in Figure 2, which could result in insoluble polymers with potentially enhanced properties. The synthesis of the tetra-amino monomer was initially planned accordingly to the same route as for the diamino-HPB previously described. However, the reaction between commercial dinitro benzil (e.g., compound 2 where R is a nitro group) and 1,3-diphenyl acetone **1b** consistently failed to produce the desired dinitro-CPD, yielding instead mixtures of products and starting materials difficult to be isolated. Given that nitro groups are electron-withdrawing and amino groups are electron-donating, we hypothesized that including amino groups in the benzil might improve the reaction. Nonetheless, this approach also proved challenging. The successful synthesis of the elusive tetra-amino HPB was achieved through a completely different approach. Initially, we prepared the tetra-bromo HPB monomer **5c** by reacting dibromo benzil **2c** with 1,3-diphenyl acetone **1b**, which produced CPD **3c**. This intermediate was then reacted with dibromo acetylene **4c**, yielding **5c** in good yields. Subsequently, the bromine atoms of **5c** were replaced with *N*-fluorene imine via a cross-coupling procedure similar to that reported by Rabbani et al.,^46^ using Pd(dba)_3_ and rac-BINAP. The resulting unstable intermediate was then hydrolyzed to obtain pure tetra-amino HPB **6c**. A detailed visual scheme of this process is provided in Scheme S1.

### Hexa-amino-HPB Monomer

To synthesize the fully substituted HPB **6d**, we began with the preparation of the simplest hexaphenylbenzene **5d**, which was obtained via a Diels–Alder reaction between commercial diphenyl acetylene **4a** and cyclopentadienone **3d**. This hydrocarbon was then nitrated using fuming HNO_3_ and H_2_SO_4_ to obtain the hexa-nitro intermediate. The nitro groups were subsequently reduced with Raney nickel and hydrazine in DMF to yield the final monomer **6d** (Figure 2).^47^ All details of each synthesis and characterization of precursors and monomers are fully described in Supporting Information (^1^H and ^13^C NMR spectra are reported in Figures S22–S40).

### Synthesis and Characterization of HPB Polymers

**Figure 3:**
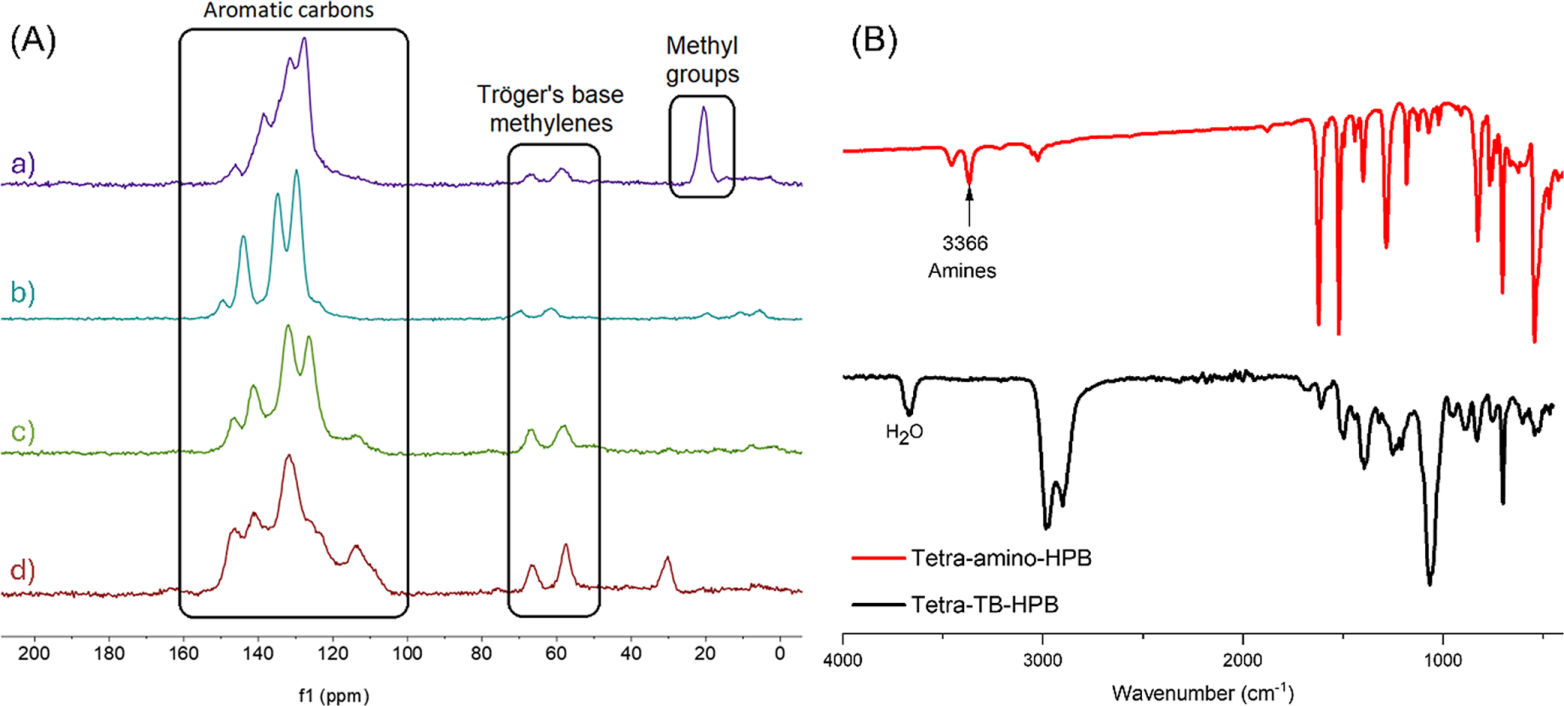
(A) Solid-state NMR of TB-HPB-PIMs: (a) TB-Me-HPB, (b) TB-HPB, (c) tetra-TB-HPB, (d) hexa-TB-HPB; (B) FT-IR of tetra-amino-HPB monomer and tetra-TB-HPB polymer.

The synthesis of all TB-PIMs was carried out using published procedures with minor modifications (Figure 1).^24^ Each amino-HPB monomer was dissolved in dichloromethane (DCM) along with dimethoxymethane as the methylene source, and trifluoroacetic acid (TFA) was added dropwise while maintaining the solution in an ice bath. The reaction was then quenched into an ice-ammonia mixture under vigorous stirring, and the product was collected by filtration as a brown powder. To purify the polymer and remove small impurities and short oligomers, the polymers were refluxed, filtered, and washed sequentially with acetone, THF, DCM, and methanol. The final product was thoroughly dried at 80 °C in a vacuum oven and characterized. In addition to the TB-HPBs, we also synthesized two “linear” TB polymers using the same polymerization method. This was done to compare the rigid and contorted TB-HPBs with the more flexible TB polymers, to evaluate the impact of reduced porosity on thermal properties and gas selectivity. Although the two ladder polymers were anticipated to be soluble, all TB-HPB-PIMs proved insoluble in organic solvents, rendering solution-based characterization methods unsuitable. Consequently, the structural features were confirmed using solid-state ^13^C NMR (SSNMR) and FT-IR spectroscopy (Figure 3A,B).

^13^C NMR spectra reveal that the peaks corresponding to aromatic carbons are distributed in the range of 150–100 ppm, consistent with the characteristics of HPB polymers. The distinctive peaks of Tröger’s base (TB) appear between 70–50 ppm, while the carbon peak from the methyl groups of TB-Me-HPB is observed at 21 ppm. Detailed SSNMR spectra are provided in the Supporting Information (Figure S1), with a comparative analysis of the two “linear” TB polymers shown in Figure S2. FT-IR spectra indicate the complete disappearance of unreacted amino groups, as exemplified by the tetra-TB-HPB polymer (Figure 3B). The peaks associated with amino groups, which are present in the HPB monomer around 3366 cm^–1^, are absent in the TB-HPB-PIM spectrum. This suggests that the polymerization sites have been fully utilized to form the TB units.

**Figure 4:**
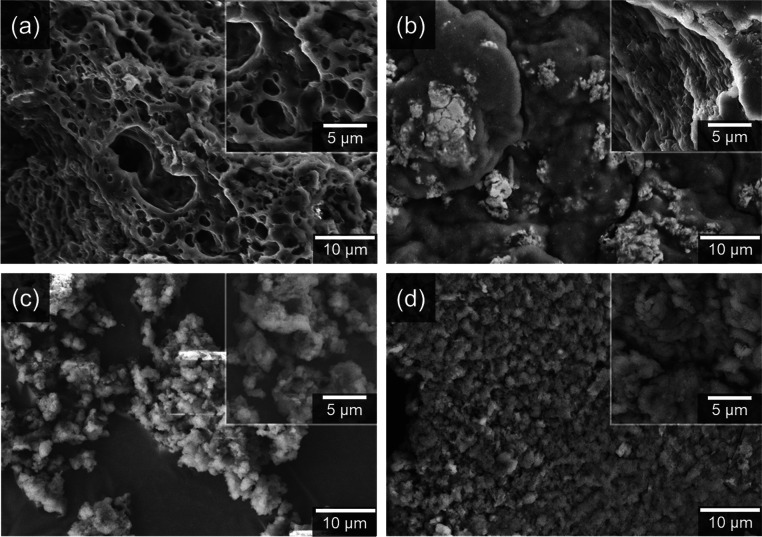
SEM of TB-HPB-PIMs: (a) TB-Me-HPB-PIM, (b) TB-HPB, (c) tetra-TB-HPB, (d) hexa-TB-HPB.

The morphological differences between the polymers are reflected in their scanning electron microscopy (SEM) images (Figure 4). The ladder-type TB-Me-HPB and TB-HPB polymers (Figure 4a,b) exhibit macropores and a smooth surface, likely due to the conformation of their ladder structures.

Despite their poor solubility in common organic solvents, these polymers appear to form a gel-like “film” which is characteristic of ladder polymers. In contrast, the networked polymers, tetra-TB-HPB and hexa-TB-HPB (Figure 4c,d), show a completely amorphous appearance with only microparticle aggregation visible. The SEM images of the “linear” TB polymers reveal a similar morphology to the ladder HPBs, with relatively flat surfaces and featuring shallow holes and cracks (Figure S21). All characterizations show that the polymers are in line with prediction, as they are very similar to previously published ladder and networked TB polymers.^24,44^

### Textural and Thermal Properties

#### Porosity and Pore Size Distribution

**Figure 5:**
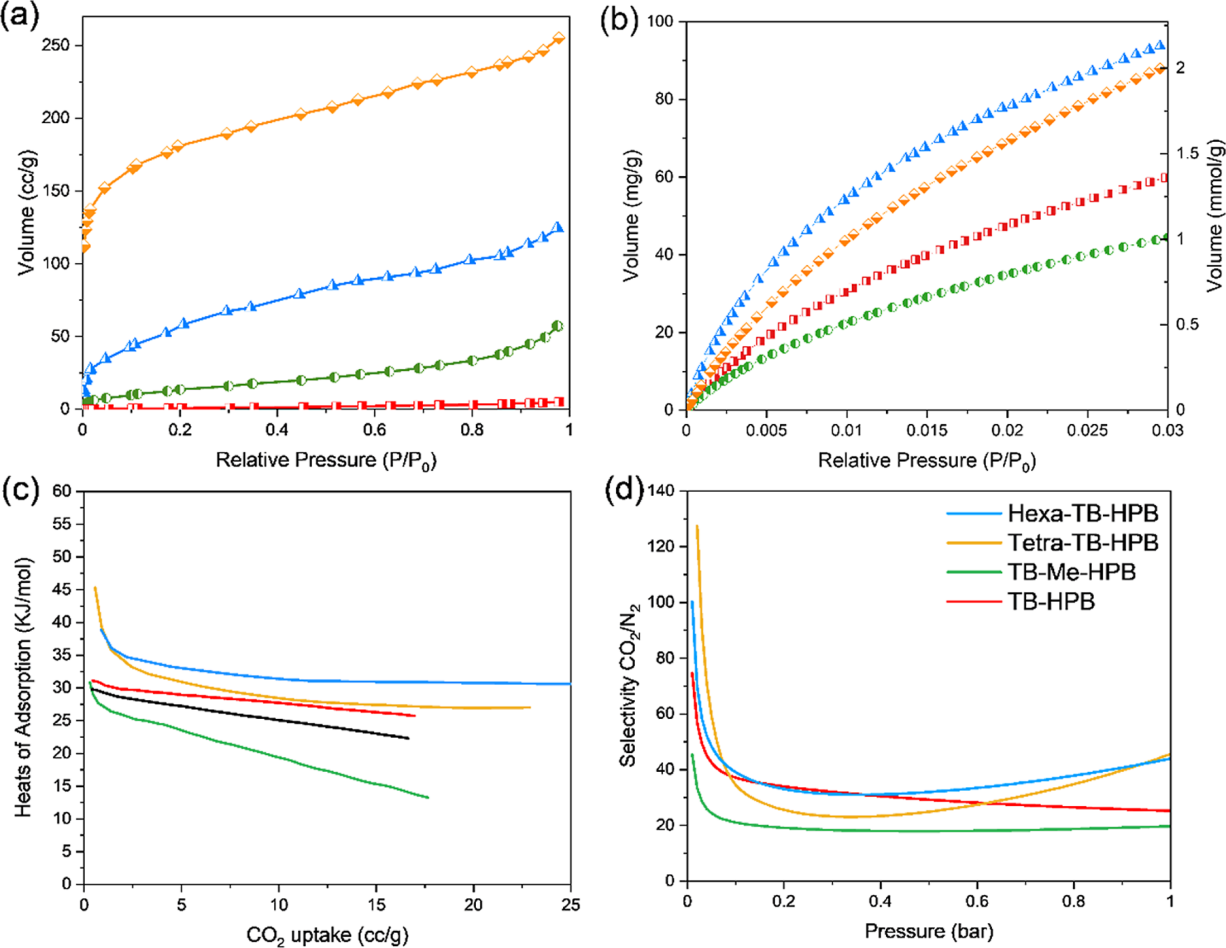
(a) N_2_ adsorption at 77 K of TB-HPB-PIMs; (b) CO_2_ adsorption at 273 K of TB-HPB-PIMs (desorption curves have been removed for clarity); (c) *Q*_st_ of TB-HPB-PIMs; (d) IAST CO_2_/N_2_ selectivity (simulating a 15/85 composition).

The porosity of the TB polymers was evaluated using N_2_ adsorption at 77 K and CO_2_ adsorption at 273 K. Tetra- and hexa-TB-HPB exhibited Type I N_2_ adsorption isotherms with high SA_BET_, indicating significant microporosity. In contrast, with this probe gas the ladder TB-HPB-PIMs displayed Type II isotherms with low SA_BET_ values (below 25 m^2^ g^–1^), suggesting that nitrogen does not effectively penetrate their small pores and that surface area measurements with N_2_ at this temperature are unreliable for these polymers (Figure 5a). Given these observations and our previous experience with flexible TB polymers,^24^ we opted to calculate the SA_BET_ from CO_2_ adsorption at both 195 and 273 K (Table 1). CO_2_, with a smaller kinetic diameter (3.30 Å) compared to N_2_ (3.64 Å), can more effectively access the pores, and higher temperatures facilitate better motion of the rigid molecular chains, which are too constrained at 77 K. Due to the similarity in SA_BET_ values at both temperatures and the convenience of measurements at 273 K, we have included in Table 1 only the values calculated at this temperature. Nevertheless, it is important to emphasize that the trend in porosity is the most critical feature for these materials, given the known limitations of BET measurements.^48,49^ The results presented in Table 1 and Figure 5b for adsorption at 273 K reveal that the ladder TB-HPB-PIMs exhibit relatively good microporosity, with SA_BET_ values ranging from 306 to 330 m^2^ g^–1^. However, appear significantly lower than those of the network polymers, tetra- and hexa-TB-HPB, which have SA_BET_ between 444 and 537 m^2^ g^–1^. The two “linear” polymers, as anticipated due to their increased flexibility, display lower porosity compared to the networked materials (275–362 m^2^ g^–1^) single N_2_ and CO_2_ adsorption isotherms are shown in Figures S10–S12. Nonetheless, they are close in porosity to the ladder PIMs, demonstrating that even by employing these simpler and commercially available monomers, materials with competitive performance can be achieved. Pore size distribution (PSD), calculated from the CO_2_ adsorption curves at 273 K using NLDFT (Figure S13), indicates that tetra and hexa-TB-HPB possess a higher proportion of narrow micropores in the range of 3–4 Å compared to the ladder TB-HPB-PIMs, which further justifies their larger SA_BET_.

**Table 1:** Physical Characterization of Polymers and Gas Selectivity

polymer	CO_2_ adsorption						
	SA_BET_^a^ (m^2^ g^–1^)	SA_BET_^b^ (m^2^ g^–1^)	pore volume^c^ (cc g^–1^)	273 K (1 bar) (mg g^–1^) (mmol g^–1^)	298 K (1 bar) (mg g^–1^) (mmol g^–1^)	CO_2_/N_2_ selectivity^d^	*Q*_st_^e^ (kJ mol^–1^)
TB-Me-HPB-PIM	22	306	0.294	44.6 (1.01)	30.2 (0.69)	19.7	30.9
TB-HPB	7	330	0.309	60.0 (1.36)	25.1 (0.57)	25.2	31.0
tetra-TB-HPB	641	537	0.349	88.2 (2.00)	47.2 (1.07)	45.6	45.3
hexa-TB-HPB	203	444	0.255	94.0 (2.14)	53.4 (1.21)	44.0	38.8
linear TB-polymer 1	54	275	0.304	53.0 (1.20)	33.3 (0.76)	18.3	29.8
linear TB-polymer 2	205	362	0.278	56.4 (1.28)	38.2 (0.87)	27.5	25.0

a Calculated from N_2_ adsorption at 77 K.

b Calculated from CO_2_ adsorption at 273 K.

c At *P*/*P*_0_ ∼ 0.98.

d Calculated according to IAST at 298 K and 1 bar.^51^

e Isosteric heat of adsorption (in kJ mol^–1^) of corresponding gas at zero coverage calculated from isotherms collected at 273 and 298 K and fitted with the Langmuir–Freundlich equation and calculated via the Clausius–Clapeyron equation.

#### Gas Adsorption and Selectivity

To assess the impact of increased nitrogen content per repeat unit, we calculated the heat of adsorption (*Q*_st_) from CO_2_ adsorption at 273 and 298 K for all polymers. Tetra-TB-HPB exhibited the highest *Q*_st_ value of 45.3 kJ mol^–1^, followed by hexa-TB-HPB at 38.8 kJ mol^–1^. The two ladder TB-HPB-PIMs showed slightly lower *Q*_st_ values of approximately 31 kJ mol^–1^. These findings align with expectations, as a higher number of basic TB sites per repeat unit was anticipated to enhance the affinity for the Lewis acidic CO_2_.

Calculating *Q*_st_ is also essential for evaluating the potential energy penalty associated with CO_2_ desorption, which is particularly relevant for carbon capture applications. Previous studies indicate that, while a strong CO_2_ affinity is advantageous, *Q*_st_ values should ideally not exceed 50 kJ mol^–1^ to prevent excessive energy expenditure during desorption.^50^ The values reported in Table 1 and shown in Figure 5c demonstrate that our materials possess an optimal balance between physisorption and chemisorption of CO_2_, providing sufficient affinity while minimizing energy costs for desorption. It is important to note that the two “linear” polymers have lower *Q*_st_ values compared to all the TB-HPBs, showing a relatively lower affinity for CO_2_ compared to the HPBs.

To better assess the potential of these materials for CO_2_ capture, we calculated the IAST selectivity for an ideal CO_2_/N_2_ (15/85) mixture, representative of flue gas (typically separated at 298 K and 1 bar). This calculation evaluates the effectiveness of our polymers in separating CO_2_ from such mixtures, providing insight into their suitability for carbon capture applications.^52,53^ The results presented in Table 1 and shown in Figure 5d demonstrate high selectivity for CO_2_/N_2_ mixtures for the two networked TB-HPBs, which have a higher nitrogen-to-carbon ratio compared to the ladder PIMs (as detailed in Table 2). Among these, tetra-TB-HPB exhibited the highest selectivity (45.6), likely due to its similar C/N ratio but higher SA_BET_ compared to the hexa-TB-HPB (44.0). In contrast, the two ladder HPB-PIMs showed lower selectivity (19.7 and 25.2), which can be attributed to their lower surface areas and higher carbon content, relative to the networked polymers.^54^

**Table 2:** Thermal Properties of TB-HPB-PIMs and Linear TB-Polymers under N_2_ and in Air^a^^,^^b^

polymer	C/N ratio	decomposition temperature (°C) under N_2_ (in air)				char yield (%)	residual mass (%)
		*T*_d5_	*T*_d10_	*T*_d20_	*T*_max_		
TB-Me-HPB-PIM	24.5	394 (351)	432 (446)	526 (509)	572 (563)	58	12
TB-HPB	22.5	432 (418)	487 (479)	565 (527)	582 (580)	63	17
tetra-TB-HPB	13	440 (378)	545 (431)	630 (481)	591 (531)	72	7
hexa-TB-HPB	8.5	458 (423)	620 (459)	616 (445)	587 (512)	70	8
linear TB-polymer 1	12.5	416 (405)			429 (539)	76	6
linear TB-polymer 2	8.5	425 (393)	448 (408)	604 (436)	436 (525)	73	5

a Under N_2_ at 1000 °C.

b In air at 600 °C.

Overall, the enhanced CO_2_ uptake and selectivity observed with an increased proportion of TB units in the TB-HPB-PIMs can be attributed to the optimal combination of several factors: the narrow microporosity of the networked polymers (Figure S13), their high SA_BET_, and their significant nitrogen content. A high surface area, in fact, provides a larger internal free volume able to accommodate more gas, while the nitrogen atoms in the tertiary amine groups create stronger interactions with CO_2_, as evidenced by the high *Q*_st_ values.

Additionally, the narrow micropores, centered around 3.5 Å, are ideally sized to facilitate the passage of smaller CO_2_ molecules while restricting the entry of larger N_2_ molecules.^16^ The two “linear” polymers, once again, demonstrated their competitiveness by closely matching the performance of the two ladder HPBs. This could be due to the similar surface areas and the fact that they are both ladder-like in nature, with the di-HPB having the TB polymerization sites in “pseudopara” positions. To obtain much better performance, it seems necessary to add an extra feature, such as the networking of the structures that cross-link the chains of the tetra- and hexa-substituted TB polymers. This highlights that, although the shape and structure of the polymer chains are crucial, substantial results can also be achieved with more cost-effective and easier-to-synthesize materials. Overall, both tetra and hexa-TB-HPB could be considered as promising candidates for real carbon capture applications. They have comparable gas selectivity and similar CO_2_ uptake. While the tetra-has the highest BET surface area, the hexa-HPB has a lower *Q*_st_, therefore it would require less energy for reactivation/desorption, which puts it in a slightly better position for CCS.

#### Thermogravimetric Analysis

**Figure 6:**
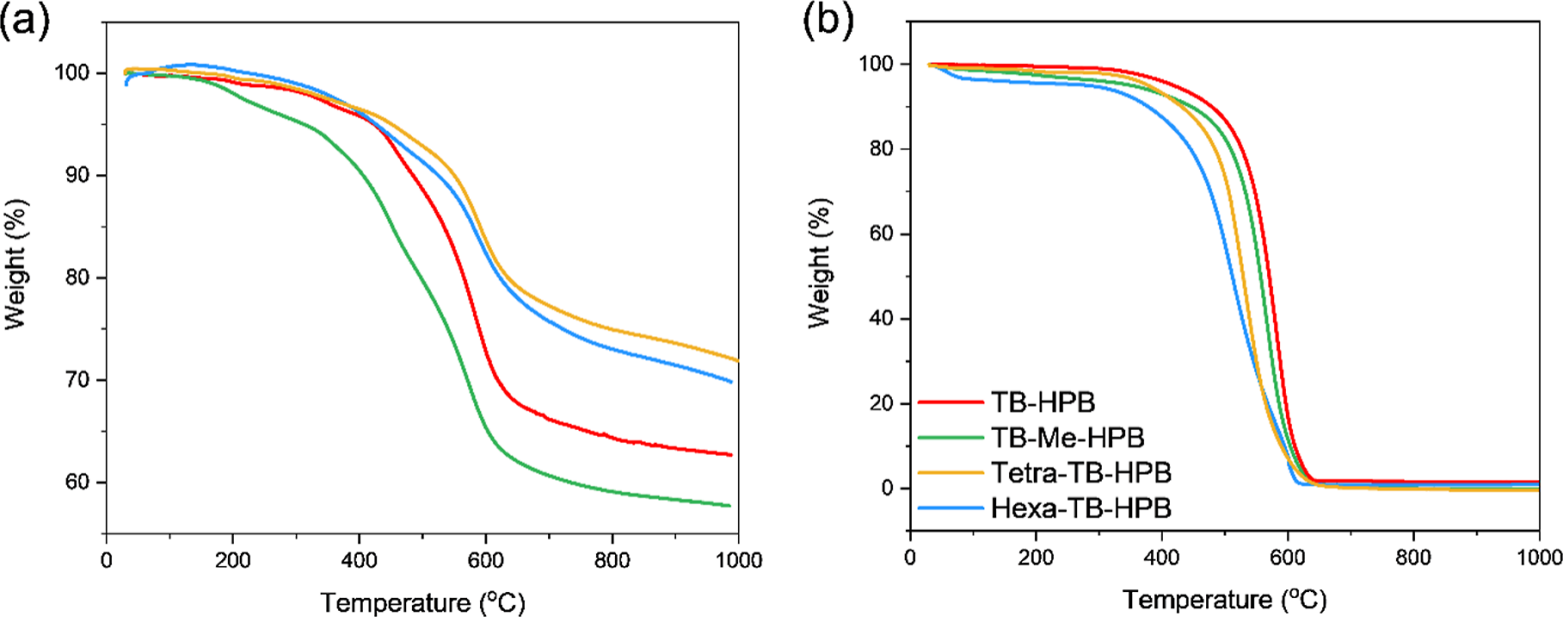
TGAs of TB-HPB-PIMs: (a) under N_2_ flow; (b) in air.

The thermal stability of polymers is closely related to their combustion behavior,^55^ and thermogravimetric analysis (TGA) is a standard method for evaluating it. By measuring the mass loss of a polymer as the temperature increases (with a typical ramp rate of 10 °C per minute), we can assess the thermal decomposition characteristics of each sample.^56,57^ Key results derived from TGA include the char yield (residual weight percent after complete decomposition) and the thermal decomposition temperature (*T*_d_). For a detailed thermal behavior assessment, *T*_d_ typically involves evaluating the maximum weight loss rate temperature (*T*_max_, calculated from DTG see Figures S14–S19), the initial decomposition temperature (*T*_i_), and temperatures corresponding to specific degrees of weight loss, such as *T*_d5_, *T*_d10_ and *T*_d20_ (respectively the temperature at 5, 10 or 20% weight loss). Polymers with higher *T*_d_ values and greater residual weights are considered to have excellent thermal stability. In our study, the thermal properties of the TB-HPB-PIMs were evaluated using TGA analysis under both N_2_ and air (with a cylinder containing 21% ± 0.5% O_2_ in N_2_) atmospheres. The TGA curves measured under N_2_ flow reveal similar mass loss for all four TB-HPB-PIMs (Figure 6). Weight losses below 355 °C, which are less than 5%, are attributed to the removal of residual solvent. Weight losses between 400 and 450 °C are associated with the decomposition and ring opening of the TB units. The final decomposition of the main polymer chains, as indicated by *T*_max_, occurs around 572–591 °C. Similarly, the two “linear” TB polymers exhibit weight losses at approximately 430 and 590 °C, respectively.

Analyzing the thermal decomposition in detail, previous research indicates a trend where a lower C/N ratio (i.e., more nitrogen per repeat unit) is associated with increased char yield.^58,59^ As shown in Table 2, polymers with C/N ratios ranging from 8.5 to 13, including tetra- and hexa-TB-HPB-PIMs as well as linear TB polymers, exhibit char yields over 70%. In contrast, for the ladder TB-HPB polymers with C/N ratios between 22.5 and 24.5, char yields decrease to approximately 60%. Comparing the hexa-TB-HPB and “linear” TB polymer 2, which have similar C/N ratios, we could conclude that porosity does not significantly impact char yield. However, it does contribute to higher *T*_d_ values, an important characteristic for flame-retardant materials. The porous structure likely helps to prevent mass and heat transfer, enhancing thermal stability. In summary, the results reported in Table 2 and Figure 6 indicate that tetra-TB-HPB exhibits the highest thermal stability among all TB-HPB-PIMs, followed by hexa-TB-HPB, and this is primarily due to its high *T*_d_ and char yield.

To assess the thermal stability of TB-HPB-PIMs under real-world conditions, TGA measurements were also conducted in an air atmosphere, yielding results that contrast with those obtained under N_2_ flow. The TGA curves for each TB-HPB-PIM showed a single pronounced weight loss concentrated between 512 and 580 °C. Notably, the highly porous tetra and hexa-TB-HPBs exhibited slightly reduced thermal stability compared to the ladder TB-HPBs. The *T*_max_ values indicate an increasing order of thermal stability: TB-HPB > TB-Me-HPB > tetra-TB-HPB > hexa-TB-HPB (Table 2 and Figure 6b). For the linear TB polymers, TGA analysis in air revealed two overlapping weight loss stages, similar to the behavior observed in N_2_. Given that the polymers generally burn completely due to the presence of oxygen, the residual mass at 600 °C is used to estimate thermal properties rather than char yield. Based on *T*_d_ values and residual mass, TB-HPB exhibits the highest thermal stability, while tetra- and hexa-TB-HPBs show slightly reduced thermal properties.

Comparing the TGA results in nitrogen and air, we conclude that porosity enhances the thermal stability of TB-HPB-PIMs in an oxygen-free environment. However, their thermal properties are significantly affected when oxygen penetrates micropores. Additionally, TB-HPB and linear TB-polymer 1, which feature dense and smooth surfaces, exhibit higher *T*_d_ values and greater residual mass in air, suggesting superior heat resistance. It is speculated that the denser surfaces may partially block the diffusion of air into the sample, thereby contributing to improved thermal stability. To further evaluate the stability of the HPB-TB polymers, we tested them in both aqueous NaOH and 6 N H_2_SO_4_ at temperatures up to 60 °C and found that they remained very stable under these conditions.

Our results align with or surpass previously reported polymers, particularly those made from similar monomeric units and nitrogen content, in terms of both gas separation performance and selectivity (as shown in Table 1) as well as thermal properties (presented in Table 2). For instance, Zhu and co-workers reported triptycene-based Tröger’s base polymers derived from separated dianiline regioisomers, with SA_BET_ values between 870 and 900 m^2^ g^–1^, generally higher than those in this work but with CO_2_/N_2_ selectivities of 20–22, which are lower than ours. The thermal properties were comparable, with decomposition temperatures ranging from 400 to 440 °C.^60^ Similarly, Xia et al. described a series of polyimides synthesized from diamine via catalytic arene–norbornene annulation (CANAL) and 4,4′-(hexafluoroisopropylidene)diphthalic anhydride (6FDA), which exhibited BET surface areas between 200 and 530 m^2^ g^–1^, comparable to those of our materials. Their reported selectivities ranged from 17 to 20, lower than the values presented in this paper, with decomposition temperatures between 450 and 490 °C, in this case closely matching our data.^61^ Kang et al. explored microporous polyimides containing pseudo-Tröger’s base units (with oxygen atoms replacing nitrogen) and 6FDA dianhydrides, achieving BET values of 161 and 172 m^2^ g^–1^ and CO_2_/N_2_ selectivities between 24 and 29. The surface area values were slightly lower than our ladder HPBs, while the selectivities were in the same range. Their thermal stability assessment showed decomposition temperatures around 421–436 °C.^62^ Ma and colleagues prepared Tröger’s base PIMs from –OH functionalized binaphthalenes, reporting surface areas between 153 and 432 m^2^ g^–1^, selectivities between 15 and 21, and thermal decomposition in the range of 385–420 °C.^63^ Zhang and co-workers synthesized Tröger’s base polymers starting from spirobischromane moieties functionalized with anilines. While surface areas were not reported, permeability data showed selectivities between 13 and 15. Their thermal decomposition values ranged from 410 to 420 °C, in line with other materials.^64^

In summary, this comparison shows that, while polymers with Tröger’s base or other nitrogen-containing units generally exhibit similar or slightly lower decomposition temperatures compared to our materials, CO_2_/N_2_ selectivities of our polymers, particularly the tetra- and hexa-phenylbenzene-based Tröger’s bases, outperform those previously reported by others.

## Conclusion

This study offers a comprehensive evaluation of the thermal stability, porosity, and CO_2_ capture performance of various TB-HPB-PIMs, including ladder and linear polymers with different nitrogen contents and pore structures. Our findings indicate that materials with higher nitrogen content, such as tetra-TB-HPB, demonstrate superior CO_2_ affinity and selectivity due to enhanced interactions with CO_2_ and optimized micropore sizes. Nonetheless, the easier to synthesize linear polymers also show competitive performance, emphasizing that effective CO_2_ capture can be achieved with more cost-effective and simpler-to-synthesize materials. Increased porosity notably improves CO_2_ capacity and selectivity, particularly in networked TB-HPB-PIMs with higher surface areas and narrower micropores, resulting in efficient CO_2_ adsorption. TGA analysis reveals that ladder-type TB-HPB-PIMs maintain substantial thermal stability in inert nitrogen atmosphere, with tetra- and hexa-TB-HPBs showing robust performance. In contrast, these polymers exhibit reduced thermal stability in oxidative air environments compared to their ladder counterparts. Conversely, denser polymers such as TB-HPB and linear TB-polymer 1 show greater thermal stability in air, likely due to their ability to restrict air diffusion. In summary, this study underscores the importance of balancing structural and compositional factors to optimize porous polymers and in particular TB-HPB-PIMs for specific applications. Tetra-TB-HPB emerges as the top performer in CO_2_ capture and thermal stability under inert conditions, while denser polymers like TB-HPB offer superior stability in oxidative environments. Thus, tetra and hexa-TB-HPB exhibit excellent overall properties and are promising candidates for applications in flame retardancy and CO_2_ adsorption.

## Supporting Information Available

Materials and methods; synthesis and characterization of monomers and polymers; adsorption studies; various figures; solid state ^13^C NMRs (PDF)
