# Supplementary material for: Highly Thermally Stable and Gas Selective Hexaphenylbenzene Tröger’s Base Microporous Polymers
Source: ACS Appl Mater Interfaces. 2024 Dec 3;16(50):69870–80. doi: 10.1021/acsami.4c15333 (PMC11660159; doi:10.1021/acsami.4c15333)
Supplement: Supplementary file 1 — am4c15333_si_001.pdf [file am4c15333_si_001.pdf]

Supporting Information

Highly Thermally Stable and Gas Selective Hexaphenylbenzene Tröger's Base Microporous Polymers

Yue Wu, Ariana R. Antonangelo, C. Grazia Bezzu and Mariolino Carta *

[a] Department of Chemistry, Swansea University, Faculty of Science and Engineering, Grove Building, Singleton Park, Swansea, SA2 8PP, UK. Email: mariolino.carta@swansea.ac.uk

Table of contents

General methods and equipment 2
IAST Selectivity Calculation 2
Synthesis of monomers 3
General procedure for substituted hexaphenylbenzenes 7
General procedure for di- and hexa-amino-hexaphenylbenzenes 9
Synthesis of Polymers 12
General procedure for TB-PIMs [16] 12
Figures 15
^{1}H NMR of monomers 15
^{13}C SSNMR of linear polymers 16
SSNMR spectra 16
FT-IR spectra 20
Gas adsorption-desorption isotherms 21
TGA and DTG 24
SEM images 26
^{1}H and ^{13}C NMR spectra of precursors and monomers 28

General methods and equipment

Commercially available reagents and gases were used without further purification. All reactions using air/moisture sensitive reagents were performed in oven-dried or flame-dried apparatus, under a nitrogen atmosphere. TLC analysis refers to analytical thin layer chromatography, using aluminium-backed plates coated with Merck Kieselgel 60 GF254. Product spots were viewed either by the quenching of UV fluorescence, and in some cases by staining with permanganate stain (Preparation: potassium permanganate (3 g) + potassium carbonate (20 g) + 5% aqueous NaOH (5 ml) + water (300 ml)). Melting points were recorded using a Cole-Parmer Stuart™ Digital Melting Point Apparatus and are uncorrected. Infrared spectra were recorded using a PerkinElmer Spectrum Two FT-IR Spectrometer. LRMS were measured using the Advion Interchim Scientific expression® compact mass spectrometer. ^{1}H NMR spectra were recorded in deuterated solvent, as stated, using an Avance Bruker DPX 500 (500 MHz) instruments, with ^{13}C NMR spectra recorded at 126 MHz. Solid-state ^{13}C NMR spectra were recorded using a Bruker Avance III spectrometer equipped with a wide-bore 9.4 T magnet (Larmor frequencies of 100.9 MHz for ^{13}C). Samples were packed into standard zirconia rotors with 4 mm outer diameter and rotated at a magic angle spinning (MAS) rate of 12.5 kHz. Spectra were recorded with cross polarisation (CP) from ^{1}H using a contact pulse (ramped for ^{1}H) of 1.5 ms. High-power ($\nu 1 \approx$ 100 kHz) TPPM-15 decoupling of ^{1}H was applied during acquisition to improve resolution. Signal averaging was carried out for 6144 transients with a recycle interval of 2 s. Chemical shifts are reported in ppm relative to $(CH_3)_4Si$ (TMS) using the CH_3 signal of L-alanine (δ = 20.5 ppm) as a secondary solid reference. Low-temperature N_2 (77 K and 298 K) and CO_2 (195 K, 273 K and 298 K) adsorption/desorption measurements of polymer powders were made using a Anton Paar Nova 600. Samples were degassed over 8 hrs at 80 °C under high vacuum prior to analysis. The gases were supplied by BOC and used without any further purification (N_2 purity > 99.999, CO_2 purity > 99.995%, air: 21% ± 0.5% oxygen, balance nitrogen). The specimen was measured twice after outgas in two different stations to minimize the error, providing the same results. The data were analysed with the software provided with the instrument. The BET surface area was calculated at a relative pressure $P/P_0 < 0.1$. NLDFT analysis was performed to calculate the pore size distribution and volume, considering a carbon equilibrium transition kernel at 273 K based on a slit-pore model; the kernel is based on a common, one centre, Lennard-Jones model. Heats of adsorption were calculated from the CO_2 curves measured at 273 K and 298 K. The data were analysed with the Anton Paar Kaomi software and fitted with the Langmuir-Freundlich equation and calculated via the Clausius-Clapeyron equation. TGAs were performed using the Perkin Elmer Thermal Analyzer STA 6000 at a heating rate of 10 °C/min from 30 to 995 °C. SEM images were recorded with a Hitachi S-4800 field emission (~1 nm resolution).

IAST Selectivity Calculation

The ideal adsorption solution theory (IAST) of Myers and Prausnitz[1] is typically used to calculate the selectivity of binary mixtures of gases from the single isotherms. The isotherms were fitted with Dual-Site Langmuir-Freundlich using the software IAST++ [2] and the selectivity (S) was calculated according to the formula:

$$S = \frac{Q_{CO_2}}{Q_{N_2}} \times \frac{P_{N_2}}{P_{CO_2}}$$

Were

P_{CO_2} is the partial pressure of CO_2

P_{N_2} is the partial pressure of N_2

Q_{N_2} is the N_2 uptake

Q_{CO_2} is the CO_2 uptake

Synthesis of monomers

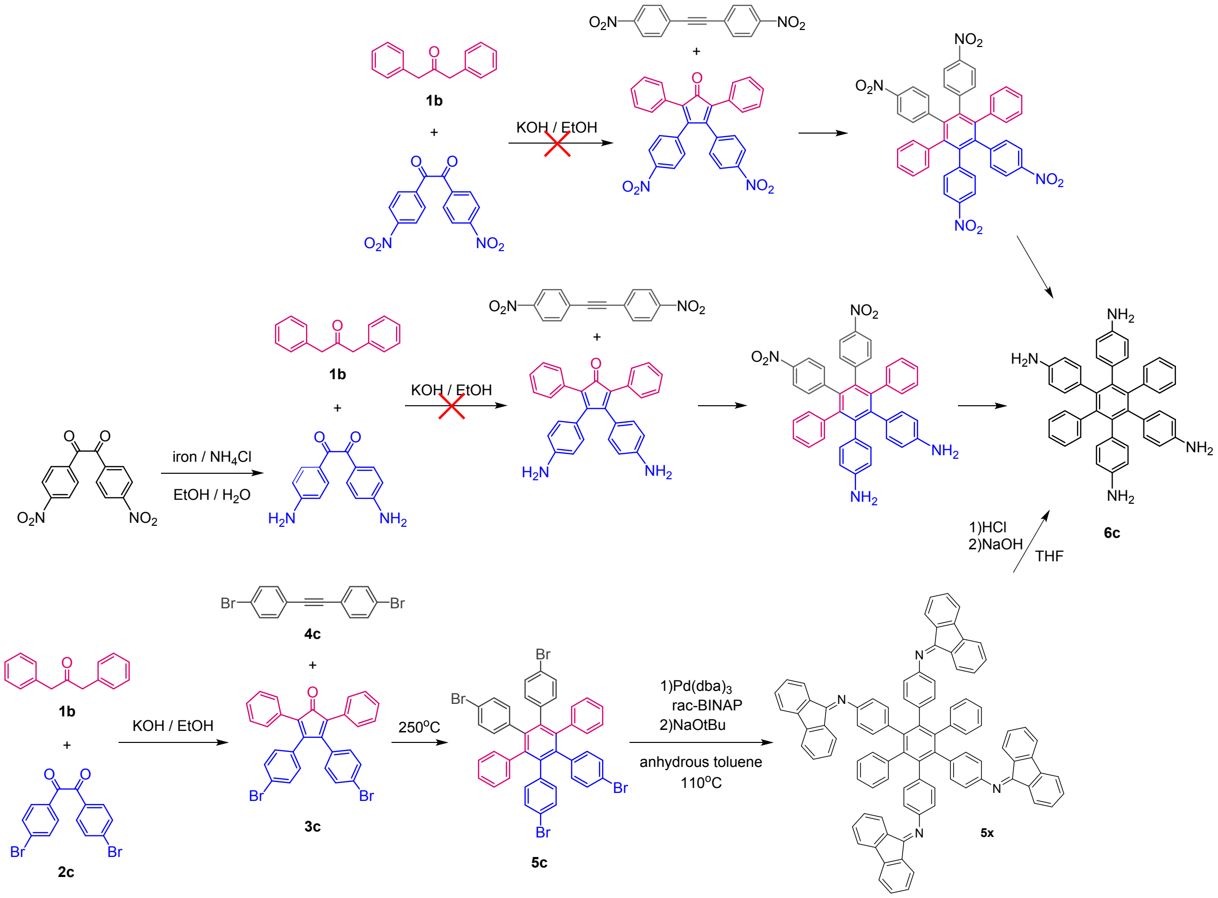

Scheme S1. Different routes for preparing tetra-amino-HPB monomer

1,3-Bis(p-nitrophenyl)acetone (1a)

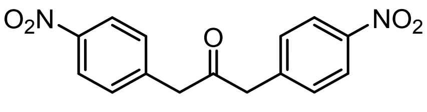

Triethylamine (30.6 mL, 0.22 mmol, 2.2 eq.) was added dropwise to a stirred solution of 4-nitrophenyl acetic acid (18.10 g, 0.1 mmol, 1.0 eq.) in 90 mL of acetic anhydride. After 1.5 hrs, the reaction mixture was slowly poured into 100 mL of cold 15% HCl and a yellow-orange solid was collected and washed with water. The solid was added to a mixture of THF (50 mL) and 9M H_2SO_4 (100 mL) and refluxed overnight. After cooling down, 50 mL of water was added into the system, and extracted with dichloromethane (3x200 mL). The organic extract was dried over anhydrous $MgSO_4$, filtered, and evaporated under reduced pressure. Then the crude product was refluxed with ethanol at 80 °C for 1 hr and hot filtered to afford a yellow solid (10.57 g, 70% yield). Mp, 179-181 °C (lit[3] 178-180 °C). ^{1}H NMR (500 MHz, Chloroform-d): δ 8.23 (d, J = 8.7 Hz, 4H), 7.37 (d, J = 8.7 Hz, 4H), 3.95 (s, 4H). ^{13}C NMR (126 MHz, Chloroform-d): δ 201.9, 147.8, 141.5, 131.4, 124.0, 49.0. LRMS (APCI+) m/z for $[C_{15}H_{12}N_2O_5]^+$: calculated 300.27, found: 300.70.

General procedure for substituted cyclopentadienones

Substituted diphenyl acetone (1.0 eq.) and benzil (1.0 eq.) were refluxed in a solution of KOH (0.5 eq.) in ethanol (10 mL per 0.1 g KOH) for 2 hrs. The reaction was allowed to cool and filtered under vacuum. The product was washed three times with water and once with ethanol to yield a dark powder.

2,5-Bis(p-nitrophenyl)-3,4-diphenyl-2,4-cyclopentadien-1-one (3a)[4]

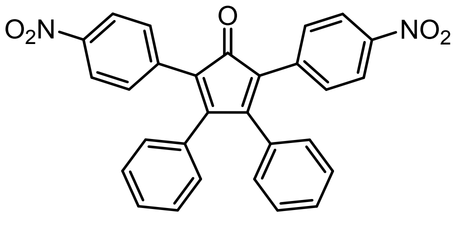

1a (4.81 g, 16.0 mmol) and benzil (3.40 g, 16.0 mmol) were refluxed in a solution of KOH (0.45 g, 8.0 mmol) in ethanol (45 mL) for 2 hrs to yield a red-black powder (5.09 g, 67% yield). Mp, 242-245 °C (lit[4] 245 °C). ^{1}H NMR (500 MHz, Chloroform-d): δ 8.14 (d, J = 8.9 Hz, 4H), 7.43 (d, J = 8.9 Hz, 4H), 7.37 (d, J = 7.5 Hz, 2H), 7.26 (d, J = 7.7 Hz, 4H), 6.93 (d, J = 8.7 Hz, 4H). ^{13}C NMR (126 MHz, Chloroform-d): δ 197.4, 157.6, 146.9, 137.1, 131.7, 130.8, 129.7, 129.0, 128.6, 124.8, 123.4. LRMS (APCI-) m/z for $[C_{29}H_{18}N_2O_5]^-$: calculated 474.46, found 474.43.

2,5-Bis(p-nitrophenyl)-3,4-bis(p-tolyl)-2,4-cyclopentadien-1-one (3b)

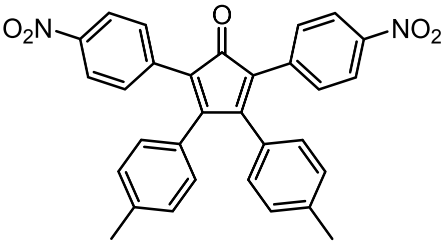

1a (4.81 g, 16.0 mmol) and 4,4'-dimethylbenzil (3.80 g, 16.0 mmol) were refluxed in a solution of KOH (0.45 g, 8.0 mmol) in ethanol (45 mL) for 2 hrs to yield a purple-black powder (4.18 g, 52% yield). Mp, 157-159 °C. ^{1}H NMR (500 MHz, Chloroform-d): δ 8.04 (d, J = 8.9 Hz, 4H), 7.33 (d, J = 8.9 Hz, 4H), 6.97 (d, J = 8.0 Hz, 4H), 6.72 (d, J = 8.1 Hz, 4H), 2.28 (s, 6H). ^{13}C NMR (126 MHz, Chloroform-d): δ 198.1, 157.8, 146.9, 137.6, 130.9, 129.9, 129.4, 129.3, 128.8, 124.0, 123.5, 21.7. LRMS (APCI+) m/z for $[C_{31}H_{22}N_2O_5]^+$: calculated 502.52, found 502.41.

2,5-Diphenyl-3,4-bis(p-bromophenyl)-2,4-cyclopentadien-1-one (3c)[5]

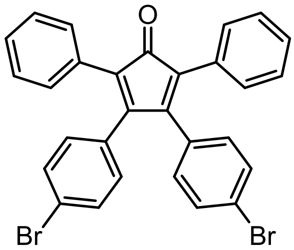

1,3-Diphenylacetone (1.05 g, 5.0 mmol) and 4,4'-dibromobenzil (1.84 g, 5.0 mmol) were refluxed in a solution of KOH (0.14 g, 2.5 mmol) in ethanol (14 mL) for 2 hrs to yield a purple-black powder (2.52 g, 83% yield). Mp, 236-238 °C (lit [5] 233 °C). ^{1}H NMR (500 MHz, Chloroform-d) δ 7.32 (d, J = 8.2 Hz, 4H), 7.24 (overlapping, m, 6H), 7.20 - 7.12 (overlapping, m, 4H), 6.76 (d, J = 8.2 Hz, 4H). ^{13}C NMR (126 MHz, Chloroform-d) δ 199.7, 152.7, 131.8, 131.7, 131.1, 130.4, 130.2, 128.4, 128.0, 126.1, 123.3. LRMS (APCI+) m/z for $[C_{29}H_{18}Br_2O]^+$: calculated 542.26, found 542.80.

1,2-Bis(p-tolyl) ethyne (4b)[6]

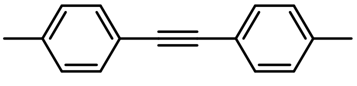

2-Butynedioic acid (2.28 g, 20.0 mmol, 1.0 eq.), $Pd(PPh_3)_4$ (1.16 g, 1.0 mmol, 0.05 eq.), 1,4-bis(diphenylphosphino)butane (0.85 g, 2.0 mmol, 0.1 eq.) and 4-bromotoluene (6.84 g, 40.0 mmol, 2.0 eq.) were combined with DBU (6.09 g, 40.0 mmol, 2.0 eq.) in 100 mL of dimethyl sulfoxide. The resulting mixture was stirred at 110 °C for 2.5 hrs. The reaction was neutralised in 42 mL of saturated aqueous ammonium chloride and extracted with diethyl ether (4×35 mL). The organic extracts were washed with brine (100 mL), dried over $MgSO_4$ and filtered. The solvent was evaporated under reduced pressure. The crude product was recrystallised in

ethanol/water. The final product was collected by filtration which afforded a yellow-brown crystal (2.05 g, 50% yield). Mp, 137-138 °C (lit [6] 136.5-137.8 °C). ^{1}H NMR (500 MHz, Chloroform-d) δ 7.34 (d, J = 8.2 Hz, 4H), 7.07 (d, J = 7.9 Hz, 4H), 2.29 (s, 6H). ^{13}C NMR (126 MHz, Chloroform-d) δ 138.2, 131.5, 129.1, 120.4, 88.9, 21.5. LRMS (APCI+) m/z for $[C_{16}H_{14}+H]^+$: calculated 207.30, found 207.14.

1,2-Bis(p-nitrophenyl) ethyne[7]

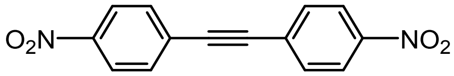

1-Iodo-4-nitrobenzene (0.45 g, 1.9 mmol, 1.0 eq.) was added with $Pd(PPh_3)_4$ (42.0 mg, 0.04 mmol, 0.02 eq.) and CuI (14.0 mg, 0.08 mmol, 0.04 eq.). Then 5 mL of acetonitrile and 5 mL of triethylamine were added to form a heterogeneous mixture. The reaction mixture was degassed by passing through nitrogen for 30 min before adding 1-ethynyl-4-nitrobenzene (0.30 g, 2.1 mmol, 1.11 eq.). The reaction was allowed to stir at room temperature under nitrogen atmosphere overnight. The reaction was diluted with 10 mL of saturated aqueous ammonium chloride and extracted with ethyl acetate (5×30 mL). Combined organic phases were washed with brine (2×80 mL), dried over $MgSO_4$ and filtered. The solvent was evaporated under reduced pressure to give crude solid product. The crude product was recrystallised in dichloromethane/petroleum ether. The final product was collected by filtration which left a yellow-brown crystal (0.40 g, 83% yield). Mp, 199-202 °C (lit [7] 205-207 °C). ^{1}H NMR (500 MHz, Chloroform-d) δ 8.26 (d, J = 8.9 Hz, 4H), 7.72 (d, J = 8.8 Hz, 4H). ^{13}C NMR (126 MHz, Chloroform-d) δ 147.8, 132.8, 129.0, 123.9, 92.1. LRMS (APCI+) m/z for $[C_{14}H_8N_2O_4]^+$: calculated 268.05, found 267.93.

4,4′-Dinitrobenzil [8]

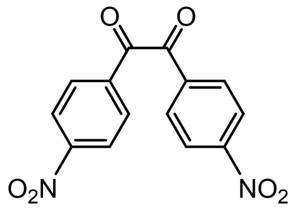

A mixture of 1,2-bis(4-nitrophenyl) acetylene (5.02 g, 18.7 mmol, 1.0 eq.), $PdCl_2$ (0.33 g, 1.9 mmol, 0.1 eq.), and 180 mL of dimethyl sulfoxide was stirred at 140 °C for 12 hrs. After being cooled, the solution was poured into water and extracted with dichloromethane. The combined organic layer was washed with brine, dried over $MgSO_4$ and filtered. The crude product that was purified by silica gel column chromatography (eluent: dichloromethane) to give a yellow powder (1.34 g, 71% yield). Mp, 210-212 °C (lit [8] 211-213 °C). ^{1}H NMR (500 MHz, Chloroform-d) δ 8.39 (d, J = 8.8 Hz, 4H), 8.22 (s, 4H). ^{13}C NMR (126 MHz, Chloroform-d) δ 190.3, 151.5, 136.7, 131.2, 124.3. LRMS (APCI+) m/z for $[C_{14}H_8N_2O_6+H]^+$: calculated 301.04 found 301.04.

4,4′-Diaminobenzil [9]

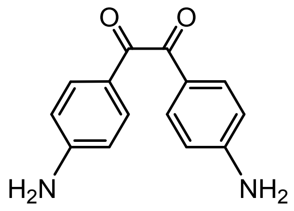

4,4′-Dinitrobenzil (0.20 g, 0.7 mmol, 1.0 eq.), iron powder (0.56 g, 10.0 mmol, 15 eq.), and NH_4Cl (0.08 g, 1.5 mmol, 2.2 eq.) were added in a mixed solvent of ethanol (16 mL) and water (6 mL) and refluxed at 80 °C for 25 hrs. After being cooled, the reaction was filtered over celite. Collected filtrate was added into 15 mL of brine and extracted with ethyl acetate. The organic phase was washed with brine three times, dried with $MgSO_4$, filtered, and evaporated. The crude product was purified by silica gel column chromatography (eluent: ethyl acetate:dichloromethane = 1:9) affording a brown powder (0.10 g, 63% yield). Mp, 169-171 °C (lit [10] 169 °C). ^{1}H NMR (500 MHz, DMSO-d_6) δ 7.51 (d, J = 8.5 Hz, 4H), 6.59 (d, J = 8.8 Hz, 4H), 6.42 (d, J = 8.7 Hz, 4H). ^{13}C NMR (126 MHz, DMSO-d_6) δ 193.8, 155.7, 132.4, 121.1, 113.4. LRMS (APCI+) m/z for $[C_{14}H_{12}N_2O_2+H]^+$: calculated 241.09, found 241.85.

General procedure for substituted hexaphenylbenzenes

Substituted cyclopentadienone (1.0 eq) and diphenyl acetylene (1.1 eq) were mixed in a hydrothermal synthesis reactor and heated in the oven at 250 °C for 2 hrs (ramped at 5 °C/min). The reaction was allowed to cool to room temperature. The solid was transferred to a sintered funnel with cold methanol, filtered and washed with warm hexane three times, and dried under vacuum affording the final products.

1,4-Bis(p-nitrophenyl)-2,3,5,6-tetraphenylbenzene (5a)

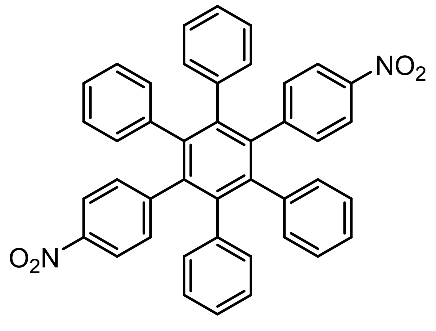

3a (4.44 g, 9.4 mmol) and diphenyl acetylene (1.83 g, 10.3 mmol) were reacted affording a yellow-brown powder (4.88 g, 84% yield). Mp >300 °C. ^{1}H NMR (500 MHz, Chloroform-d): δ 7.75 (d, J = 8.8 Hz, 4H), 7.01 (d, J = 8.8 Hz, 4H), 6.89 (m, 12H), 6.80 (m, 8H). ^{13}C NMR (126 MHz, Chloroform-d): δ 148.0, 145.7, 140.5, 139.3, 132.2, 131.2, 127.3, 126.3, 122.2. LRMS (APCI+) m/z for $[C_{42}H_{28}N_2O_4]^+$: calculated 624.68, found 624.31.

1,4-Bis(p-nitrophenyl)-2,3,5,6-tetra(p-tolyl)benzene (5b)

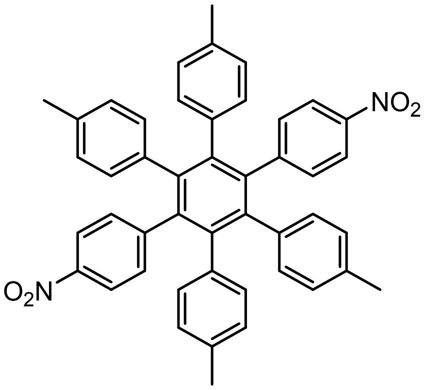

3b (1.00 g, 2.0 mmol) and bis(p-tolyl) ethyne (0.45 g, 2.2 mmol) were reacted affording a yellow-brown powder (0.93 g, 68% yield). Mp >300 °C. ^{1}H NMR (500 MHz, Chloroform-d): δ 7.74 (d, *J* = 8.7 Hz, 4H), 6.96 (d, *J* = 8.8 Hz, 4H), 6.69-6.63 (overlapping, m, 16H), 2.11 (s, 12H). ^{13}C NMR (126 MHz, Chloroform-d): δ 148.6, 145.5, 140.4, 139.6, 136.5, 135.6, 132.2, 131.1, 128.0, 122.1, 21.2. LRMS (APCI+) m/z for $[C_{46}H_{36}N_2O_4]^+$: calculated 680.79, found 680.61.

1,2,4,5-Tetra(p-bromophenyl)-3,6-diphenylbenzene (5c)

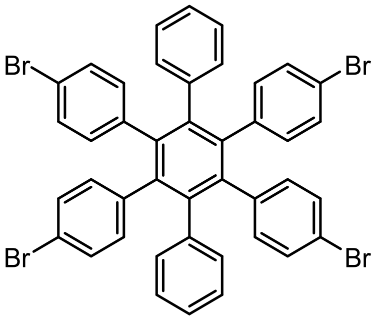

3c (2.30 g, 4.2 mmol) and bis(4-bromophenyl) acetylene (1.56 g, 4.6 mmol) were reacted affording a pale pink powder (3.44 g, 95% yield). Mp >300 °C. ^{1}H NMR (500 MHz, DMF-d7) δ 7.70 (d, *J* = 8.2 Hz, 8H), 7.58 (d, *J* = 8.2 Hz, 8H), 7.50 – 7.31 (m, 4H), 7.30 – 7.10 (m, 4H), 6.96 (t, *J* = 7.9 Hz, 2H). ^{13}C NMR (126 MHz, DMF-d7) δ 147.3 134.5, 133.1, 123.7, 90.4. LRMS (APCI+) m/z for $[C_{42}H_{36}Br_4]^+$: calculated 850.29, found 850.54.

Hexaphenylbenzene (5d)

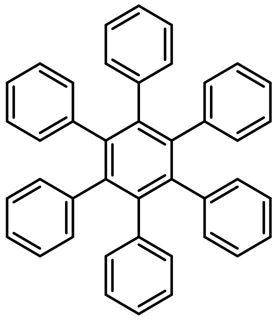

Tetraphenylcyclopentadienone (3.84 g, 10.0 mmol) and diphenyl acetylene (1.96 g, 11.0 mmol) were reacted affording a pale pink powder (4.99 g, 93% yield). Mp >300 °C (lit [11] >300 °C). ^{1}H NMR (500 MHz, $CDCl_3$) δ 6.98 – 6.79 (m, 30H). ^{13}C NMR (126 MHz, $CDCl_3$) δ 140.6, 140.3, 131.4, 126.6, 125. LRMS (APCI+) m/z for $[C_{42}H_{30}]^{+}$: calculated 534.70, found 535.14.

Hexa(p-nitrophenyl) benzene [12]

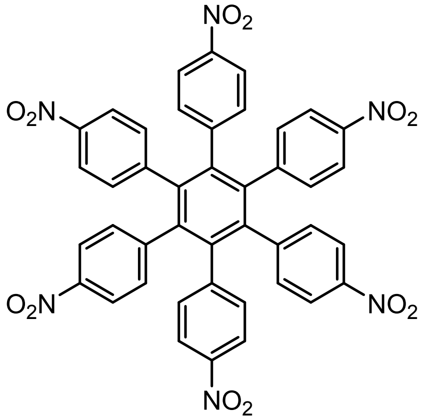

Hexaphenylbenzene (1.60 g, 3.0 mmol) was slowly added into a mixture of 37 mL of fuming HNO_3 and 18 drops of H_2SO_4 at 0 °C. Then 7.3 mL of acetic acid and 3.7 mL of ethyl acetate was mixed and added into the reaction dropwise. The reaction was stirred for 20 hrs at room temperature and poured into 140 mL of cold water. The resulting precipitate was collected by filtration, washed twice with acetone, dried under vacuum, and then recrystallized from dimethyl sulfoxide. The crystals were isolated by filtration and washed three times with acetone to afford a yellow solid (2.34 g, 97% yield). Mp >300 °C (lit [12]>360 °C). ^{1}H NMR (500 MHz, DMF-d7) δ 8.08 (d, J = 7.8 Hz, 12H), 7.60 (d, J = 8.4 Hz, 12H). ^{13}C NMR (126 MHz, DMF-d7) δ 147.4, 146.8, 140.1, 133.7, 123.6. LRMS (APCI-) m/z for $[C_{42}H_{24}N_6O_{12}\text{-}H]^{-}$: calculated 803.68, found 803.64.

General procedure for di- and hexa-amino-hexaphenylbenzenes

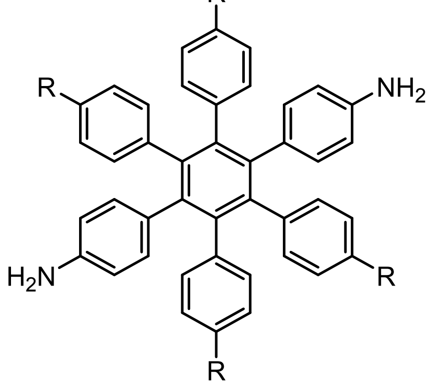

Nitro-substituted hexaphenylbenzene was added to an appropriate amount of THF and stirred under N_2 atmosphere for 15 min. A spatula tip of Raney Nickel was quickly added into the solution, followed by dropwise adding hydrazine monohydrate (5.0 eq. per 1.0 eq. of nitro group). The reaction was refluxed at 60 °C overnight and then cool to room temperature. The

Raney Nickel was isolated by filtration over celite, washed with warm THF and disposed into aqua regia. Collected filtrate was evaporated under reduced pressure. The raw product was refluxed in methanol overnight and hot filtered to afford a brown solid.

1,4-Bis(p-aminophenyl)-2,3,5,6-tetraphenylbenzene (R=H) (6a)

The reaction of **5a** (1.56 g, 2.5 mmol) and hydrazine monohydrate (1.2 mL, 25.0 mmol) in 35 mL of THF produced the final product (0.71 g, 51% yield). Mp >300 °C (lit [13] 453 °C). ^{1}H NMR (500 MHz, Chloroform-d) δ 6.85 (m, 20H), 6.57 (s, 4H), 6.23 (s, 4H), 3.31 (s, 4H). ^{13}C NMR (126 MHz, Chloroform-d): δ 143.4, 141.3, 140.7, 134.0, 132.5, 131.7, 131.4, 126.7, 125.1, 113.9. LRMS (APCI+) m/z for $[C_{42}H_{32}N_2]^+$: calculated 564.71, found 564.69.

1,4-Bis(p-aminophenyl)-2,3,5,6-tetra(p-tolyl) benzene (R=CH$_3$) (6b)

The reaction of **5b** (2.72 g, 4.0 mmol) and hydrazine monohydrate (2.0 mL, 40.0 mmol) in 60 mL of THF produced the crude product which was purified by silica gel column chromatography (eluent: ethyl acetate:petroleum ether = 2:3) to yield the final product (0.20 g, 23% yield). Mp >300 °C. ^{1}H NMR (500 MHz, Chloroform-d) δ 6.68 (s, 16H), 6.57 (d, *J* = 8.3 Hz, 4H), 6.22 (d, *J* = 8.4 Hz, 4H), 3.15 (s, 4H), 2.12 (s, 12H). ^{13}C NMR (126 MHz, Chloroform-d): δ 143.1, 140.6, 140.2, 138.4, 134.0, 132.4, 132.0, 131.5, 127.3, 113.9, 21.2. LRMS (APCI+) m/z for $[C_{46}H_{40}N_2]^+$: calculated 620.84, found 621.04.

Hexa(p-aminophenyl) benzene (R=NH$_2$) (6d)

The reaction of **5d** (2.00 g, 2.5 mmol) and hydrazine monohydrate (3.6 mL, 75.0 mmol) in 35 mL of N, N-dimethylformamide produced the final product (2.59 g, 83% yield). Mp >300 °C ^{1}H NMR (500 MHz, DMSO-d6) δ 6.36 (d, *J* = 8.1 Hz, 12H), 6.03 (d, *J* = 8.2 Hz, 12H), 4.49 (s, 12H). ^{13}C NMR (126 MHz, Chloroform-d): δ 145.0, 140.7, 132.0, 130.3, 113.2. LRMS (APCI+) m/z for $[C_{42}H_{36}N_6+H]^+$: calculated 625.80 found 626.65.

1,2,4,5-Tetra(p-(N-diphenylmethylene) phenyl)-3,6-diphenylbenzene [14]

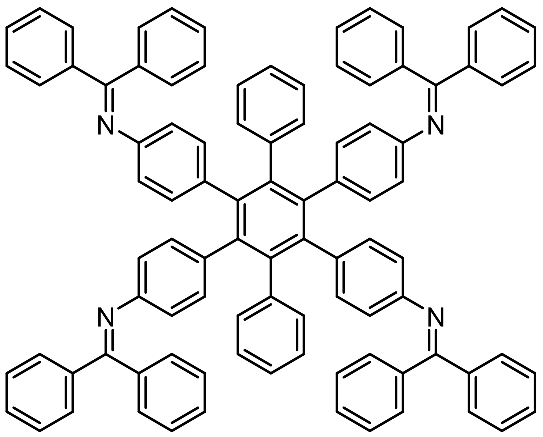

Tris(dibenzylideneacetone)dipalladium (0) (2.19 g, 2.4 mmol, 0.5 eq.) and rac-BINAP (2.96 g, 4.8 mmol, 1.0 eq.) were charged in a 250 mL round bottom flask under N_2 atmosphere and 116 mL of anhydrous toluene was added. The solution was stirred at 110 °C for 30 min under N_2 atmosphere then cooled to room temperature. To the resultant solution was added **5c** (4.08 g,

4.8 mmol, 1.0 eq.), benzophenone imine (4.2 mL, 25.0 mmol, 5.2 eq.), sodium tert-butoxide (2.40 g, 25.0 mmol, 5.2 eq.), and the mixture was stirred at 110 °C for 24 hrs under N_2 atmosphere. The system was cooled to room temperature, diluted with DCM then filtered and evaporated under reduced pressure. The residue was subjected to column chromatography on silica gel (eluent: 2% of methanol in DCM) and evaporated under reduced pressure to afford an orange solid (2.82 g, 47% yield). Mp >300 °C. ^{1}H NMR (500 MHz, Chloroform-d) δ 7.68 (d, J = 7.2 Hz, 8H), 7.42 (t, J = 7.3 Hz, 4H), 7.36 (t, J = 7.6 Hz, 8H), 7.28 – 7.22 (overlapping, m, 12H), 6.93 (d, J = 5.9 Hz, 8H), 6.83 (overlapping, m, 6H), 6.66 (d, J = 6.9 Hz, 4H), 6.49 (d, J = 8.1 Hz, 8H), 6.22 (d, J = 8.1 Hz, 8H). ^{13}C NMR (126 MHz, Chloroform-d) δ 167.5, 148.0, 140.8, 140.5, 140.1, 139.9, 137.7, 136.0, 135.8, 132.4, 131.6, 131.4, 130.4, 130.1, 129.6, 129.2, 128.4, 128.3, 128.1, 127.9, 126.7, 125.1, 119.6, 77.3, 77.0, 76.8, 53.4. LRMS (APCI+) m/z for $[C_{94}H_{58}N_4]^+$: calculated 1243.53, found 1243.03.

1,2,4,5-Tetra(p-aminophenyl)-3,6-diphenylbenzene (6c) [14]

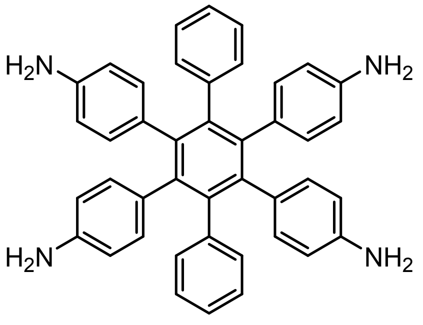

2 M aqueous HCl solution (5.7 mL, 11.4 mmol, 1.1 eq.) was added to a solution of 1,2,4,5-tetra(p-(N-diphenylmethylene) phenyl)-3,6-diphenylbenzene (3.28 g, 2.6 mmol, 1.0 eq.) in THF (150 mL), and the mixture was stirred at room temperature for 1 hr. The precipitate was isolated by filtration, washed with THF and petroleum ether, and dried under vacuum to afford the tetraammoniumhexaphenylbenzene tetrachloride salt as a pale-yellow solid (0.59 g, 42% yield). Mp >300 °C. ^{1}H NMR (500 MHz, DMSO-d6) δ 9.67 (s, 8H), 6.98 (d, J = 7.8 Hz, 8H), 6.90 (d, J = 5.8 Hz, 8H), 6.87 – 6.68 (overlapping, m, 10H). ^{13}C NMR (126 MHz, DMSO) δ 140.9, 139.8, 139.7, 132.4, 131.2, 130.1, 127.3, 126.4, 121.7. The neutralization was carried out by stirring a suspension of the salt (0.44 g, 0.6 mmol, 1.0 eq.) in 15 mL of THF with 1 M aqueous NaOH solution (2.6 mL, 2.6 mmol, 4.4 eq.). The resulting solid was filtered, washed with THF and petroleum ether, and dried under vacuum to give an off-white solid (0.21 g, 59% yield). Mp >300 °C ^{1}H NMR (500 MHz, DMSO-d6) δ 6.84 (t, J = 7.3 Hz, 4H), 6.77 (overlapping, m, 6H), 6.39 (d, J = 8.0 Hz, 8H), 6.01 (d, J = 8.0 Hz, 8H), 4.53 (s, 8H). ^{13}C NMR (126 MHz, DMSO) δ 145.4, 142.3, 141.0, 140.4, 131.9, 131.6, 129.3, 126.7, 125.1, 113.1. LRMS (APCI+) m/z for $[C_{42}H_{34}N_4]^+$: calculated 594.76, found 595.02.

*Note: **6c** is air sensitive (presumably due to amine oxidation) and therefore we used its HCl-salt form in subsequent polymerization steps.*

3,2',5',3''-Tetramethyl-4,4''-diamino-p-terphenyl[15]

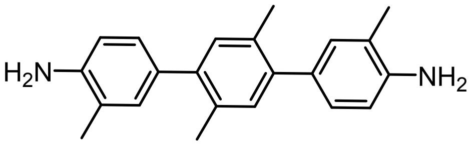

Synthesis procedure refer to published literature, affording a light brown solid (68% yield). Mp, 176-177 °C (lit [15] 177−179 °C). ^{1}H NMR (500 MHz, Chloroform-d) δ 7.12 (s, 2H), 7.10 – 7.03 (m, 4H), 6.74 (d, J = 7.9 Hz, 2H), 3.65 (s, 4H), 2.29 (s, 6H), 2.23 (s, 6H). ^{13}C NMR (126 MHz, Chloroform-d) δ 143.4, 140.4, 132.6, 132.4, 132.0, 131.5, 128.0, 122.1, 114.7, 25.0, 20.2, 17.6. LRMS (APCI+) m/z for $[C_{22}H_{24}N_2]^+$: calculated 316.45, found 317.03.

Synthesis of Polymers

General procedure for TB-PIMs [16]

The aromatic amine was dissolved in dimethoxymethane (2.0-2.5 eq. per 1.0 eq. of amino group) at 0 °C. An appropriate amount of trifluoroacetic acid was added dropwise, and the mixture was stirred at room temperature for 2 to 4 days. A modest amount of dichloromethane was allowed to be added if needed (to better dissolve the aromatic amine). The reaction mixture was then slowly poured into vigorously stirred aqueous ammonium hydroxide solution (100 mL) at 0 °C and left for 4 hrs. The resulting solid was collected by filtration, washed with water and refluxed with acetone, tetrahydrofuran, dichloromethane and methanol in turn. The final polymer was filtered and dried under vacuum at 80 °C overnight. The polymers were analysed by FT-IR spectroscopy, TGA, and isothermal gas adsorption (SA_{BET} was calculated from CO_2 adsorption isotherm at 273 K).

TB-HPB-PIM

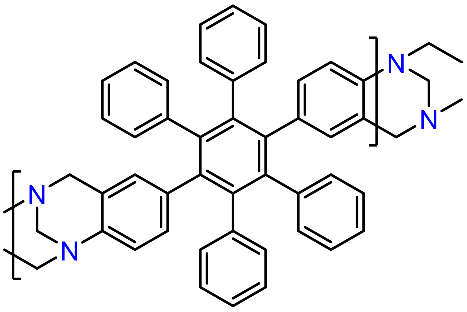

The reaction of **6a** (0.71 g, 1.2 mmol, 1.0 eq.), dimethoxymethane (0.6 mL, 6.2 mmol, 5.0 eq.), trifluoroacetic acid (2.8 mL, 37.2 mmol, 30.0 eq.) and 10 mL of dichloromethane produced di-TB-HPB-PIM (0.70 g, 94% yield). SA_{BET}= 238 m^{2}g^{-1}, CO_2 adsorption at 273 K/1 bar= 1.36 mmol g^{-1}, T_{max}= 582 °C. FTIR-ATR (cm^{-1}): 1065, 1201, 1493, 3029, 3048, 3742. ^{13}C SSNMR (101 MHz) δ 146.1, 138.6, 131.6, 127.8, 66.7, 58.9, 20.6.

TB-Me-HPB-PIM

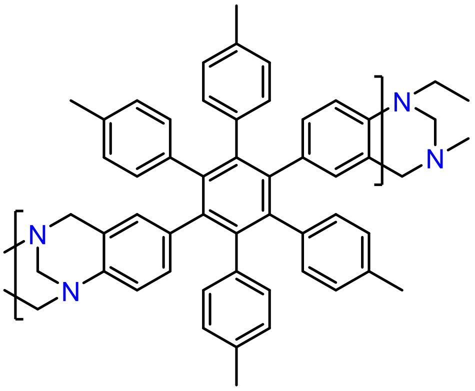

The reaction of **6b** (1.00 g, 1.6 mmol, 1.0 eq.), dimethoxymethane (0.7 mL, 8.1 mmol, 5.0 eq.), trifluoroacetic acid (3.7 mL, 48.3 mmol, 30.0 eq.) and 25 mL of dichloromethane produced di-TB-tetramethyl-HPB-PIM (0.84 g, 89% yield). SA_{BET}= 242 m^2g^{-1}, CO_2 adsorption at 273 K/1 bar= 1.01 mmol g^{-1}, T_{max}= 572 °C. FTIR-ATR (cm^{-1}): 1064, 1514, 2906, 2974, 3668. ^{13}C SSNMR (101 MHz) δ 146.1, 138.6, 131.6, 127.8, 66.7, 58.9, 20.6.

Tetra-TB-HPB-PIM

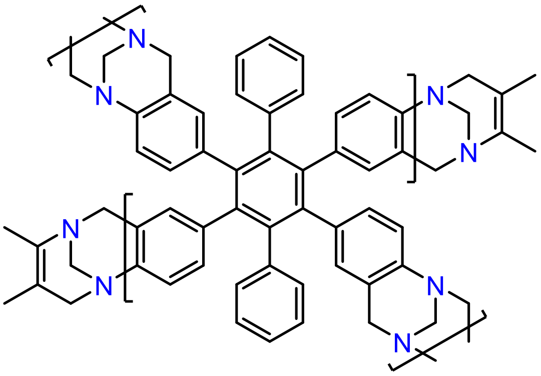

The reaction of **6c** (0.79 g, 1.1 mmol, 1.0 eq.), dimethoxymethane (0.9 mL, 10.6 mmol, 10.0 eq.), trifluoroacetic acid (4.9 mL, 63.6 mmol, 60.0 eq.) and 11 mL of dichloromethane produced tetra-TB-HPB-PIM (0.67 g, 88% yield). SA_{BET}= 516 m^2g^{-1}, CO_2 adsorption at 273 K/1 bar= 2.00 mmol g^{-1}, T_{max}= 591 °C. FTIR-ATR (cm^{-1}): 1065, 2899, 2981, 3668. ^{13}C SSNMR (101 MHz) δ 146.4, 141.3, 131.9, 126.5, 113.1, 67.4, 57.8.

Hexa-TB-HPB-PIM

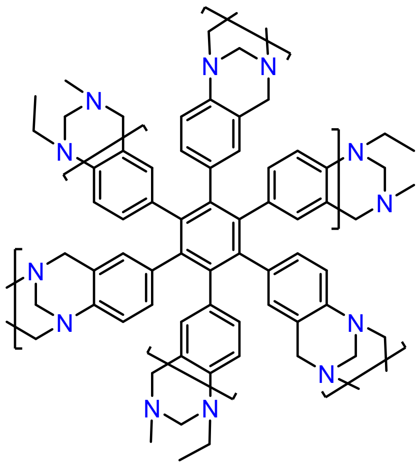

The reaction of **6d** (0.50 g, 0.8 mmol, 1.0 eq.), dimethoxymethane (0.9 mL, 9.6 mmol, 12.0 eq.) and trifluoroacetic acid (6.5 mL, 85.0 mmol, 106.0 eq.) produced hexa-TB-HPB-PIM (0.51 g, 83% yield). SA_{BET}= 444 m^2g^{-1}, CO_2 adsorption at 273 K/1 bar= 2.14 mmol g^{-1}, T_{max}= 587 °C. FTIR-ATR (cm^{-1}): 1064, 2899, 2981, 3667. ^{13}C SSNMR (101 MHz) δ 146.6, 141.4, 131.7, 113.4, 66.8, 57.5.

Linear TB-polymer 1

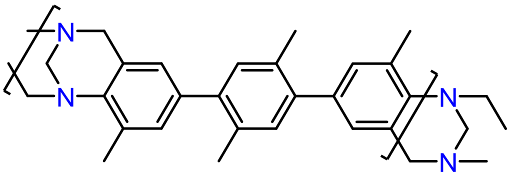

The reaction of 3,2',5',3''-tetramethyl-4,4''-diamino-p-terphenyl (0.80 g, 3.2 mmol, 1.0 eq.), dimethoxymethane (1.1 mL, 12.6 mmol, 4.0 eq.), trifluoroacetic acid (5.6 mL, 72.7 mmol, 23.0 eq.) and 5.5 mL of dichloromethane produced linear TB-polymer 1 (0.60 g, 54% yield). SA_{BET}= 275 m^2g^{-1}, CO_2 adsorption at 273 K/1 bar= 1.20 mmol g^{-1}, T_{max}= 429 °C. FTIR-ATR (cm^{-1}): 866, 941, 1213, 1471, 2915, 2945. ^{13}C SSNMR (101 MHz) δ 141.7, 137.4, 134.6, 128.3, 124.8, 64.5, 52.1, 14.3.

Linear TB-polymer 2

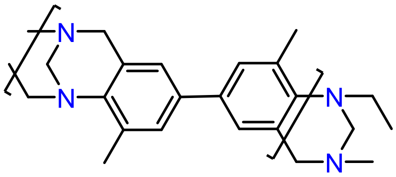

The reaction of 2-tolidine (0.42 g, 2.0 mmol, 1.0 eq.), dimethoxymethane (0.7 mL, 8.0 mmol, 4.0 eq.), trifluoroacetic acid (4.6 mL, 60.0 mmol, 30.0 eq.) and 10 mL of dichloromethane produced linear TB-polymer 3 (0.46 g, 98% yield). SA_{BET}= 362 m^2g^{-1}, CO_2 adsorption at 273 K/1 bar= 1.28 mmol g^{-1}, T_{max}= 436 °C. FTIR-ATR (cm^{-1}): 857, 1067, 1211, 1470, 2885, 2972. ^{13}C SSNMR (101 MHz) δ 145.2, 138.1, 132.2, 127.9, 67.6, 55.1, 16.2.

Figures

¹H NMR of monomers

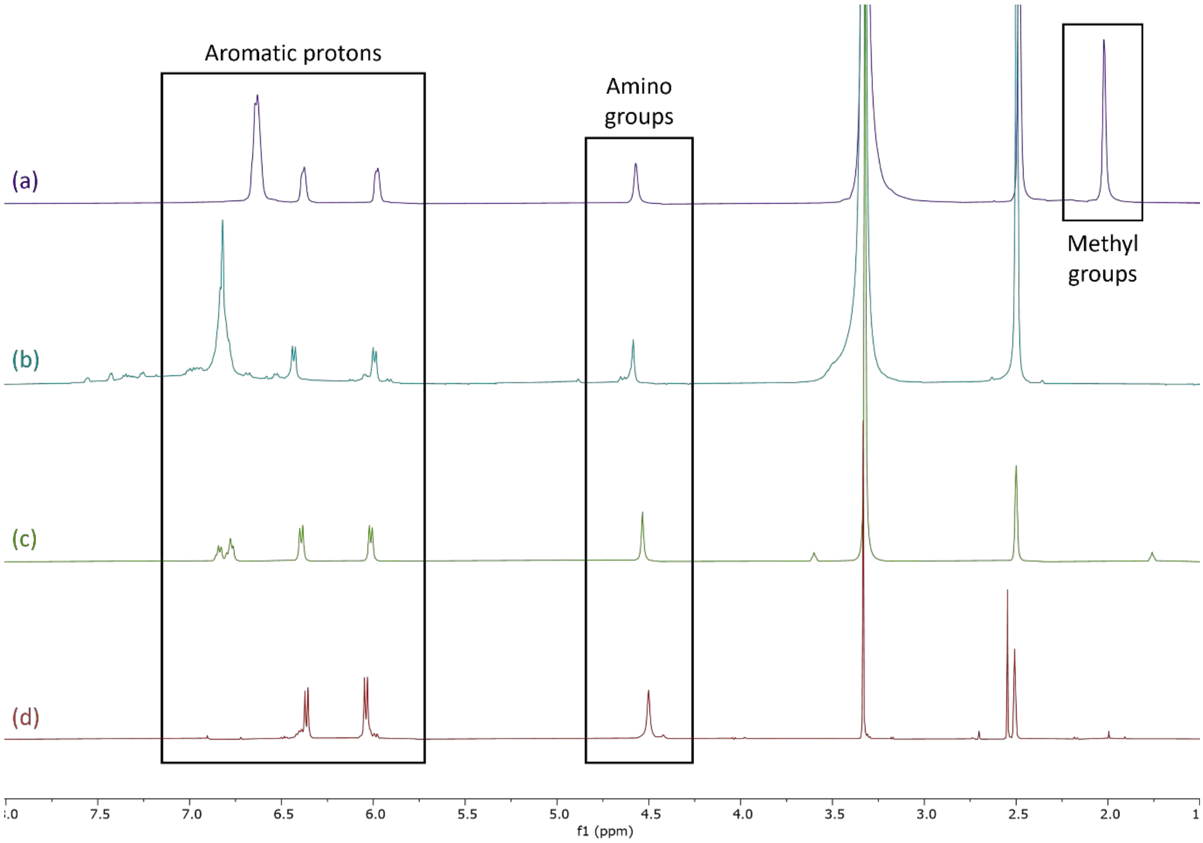

Figure S1. Proton NMR of diamino-HPB monomers:(a) diamino-Me-HPB, (b) diamino-HPB, (c) tetraamino-HPB, (d) hexaamino-HPB.

^{13}C SSNMR of linear polymers

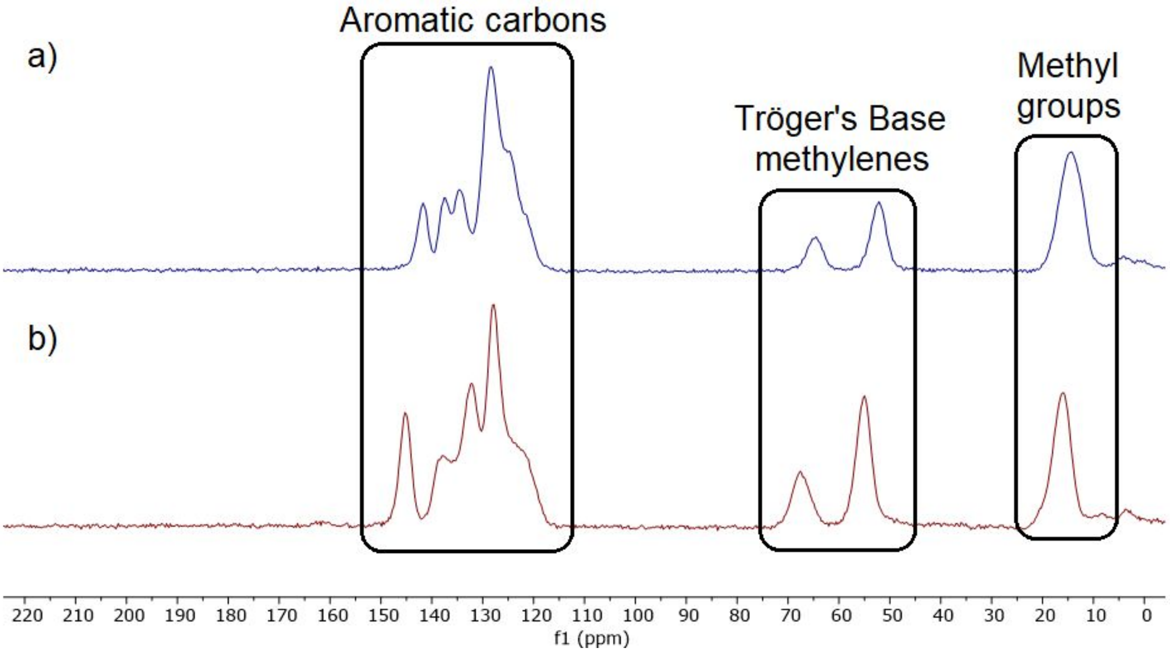

Figure S2. ^{13}C solid state NMR of linear-TB-polymers: (a) linear TB-polymer 1, (b) linear TB-polymer 2.

SSNMR spectra

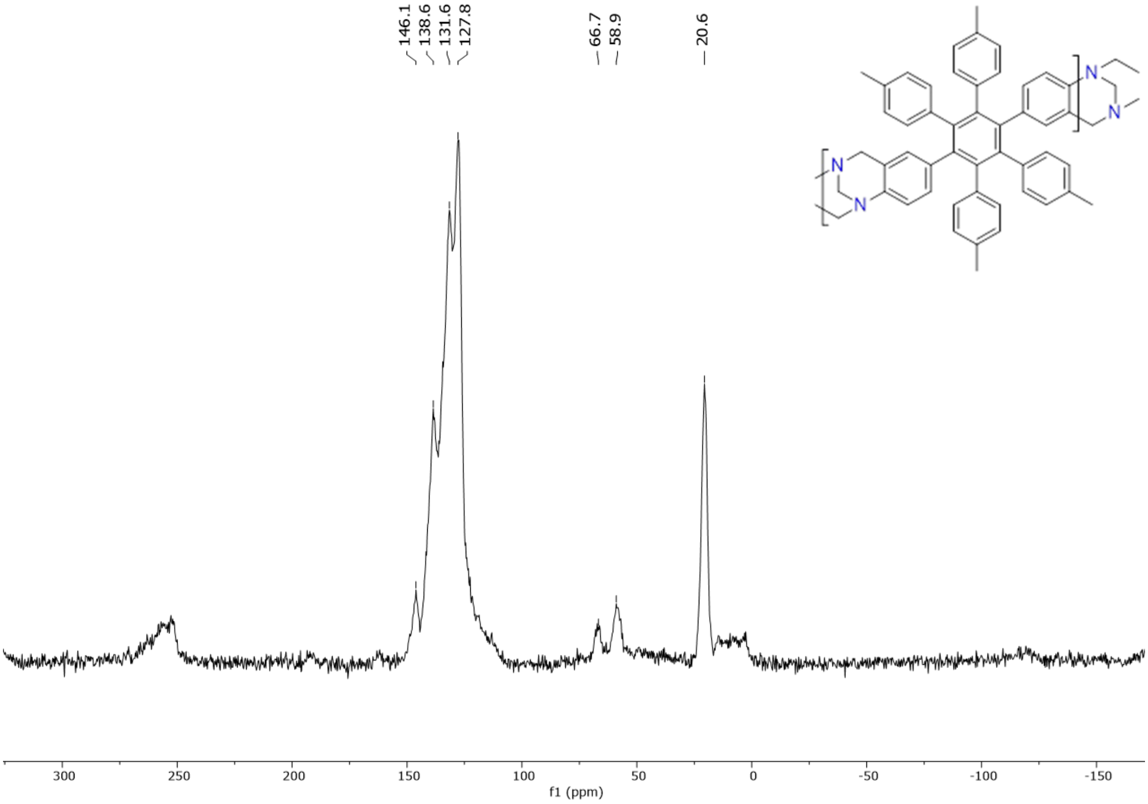

Figure S3. SSNMR for di-TB-tetra-Me-HPB-PIM.

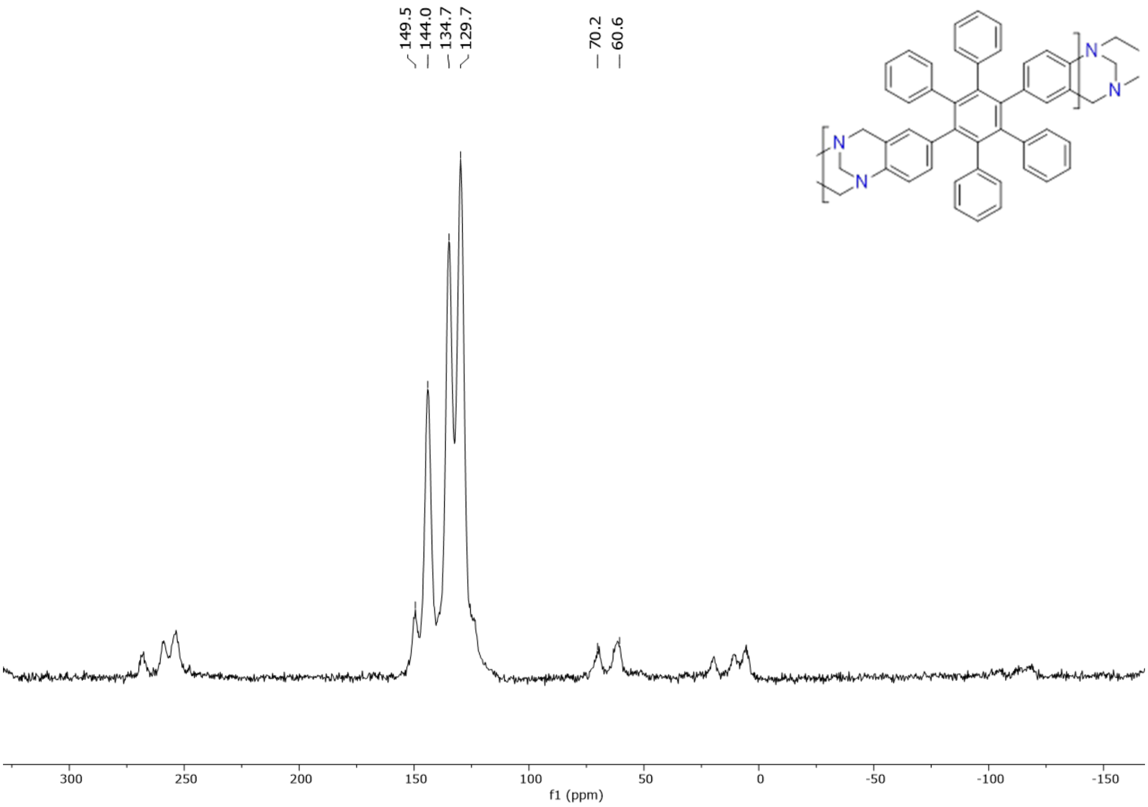

Figure S4. SSNMR for di-TB-HPB-PIM.

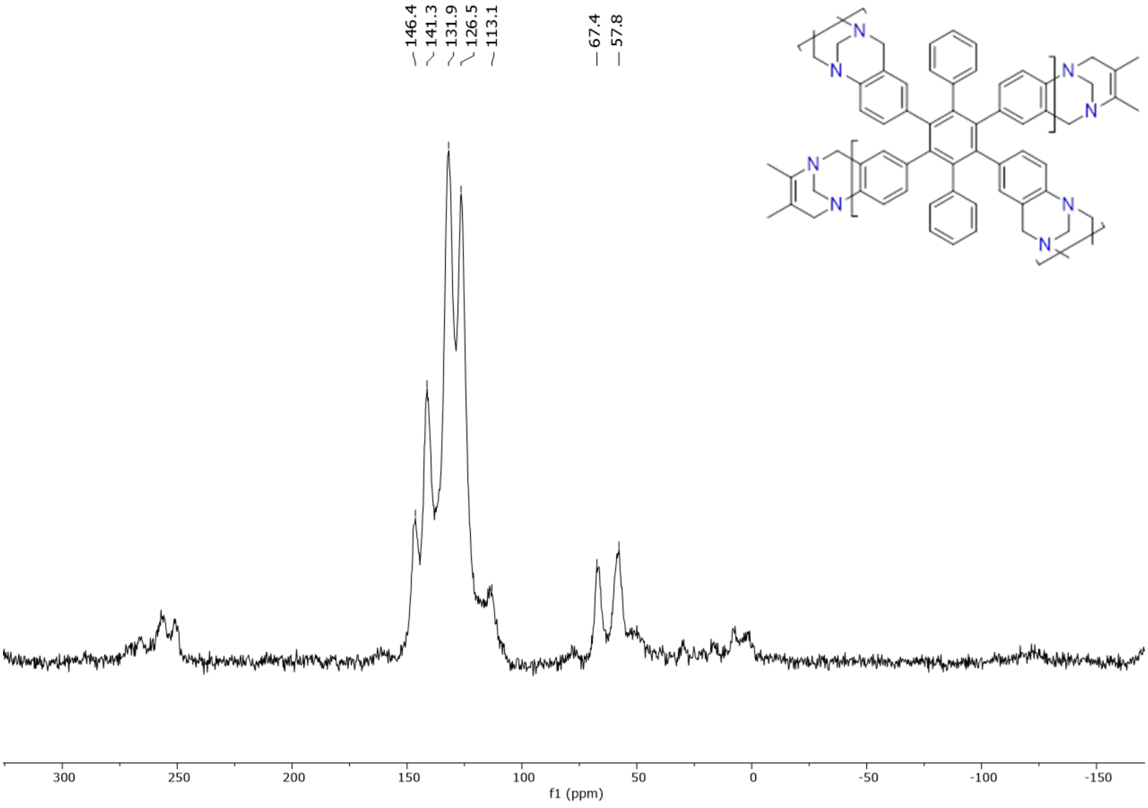

Figure S5. SSNMR for tetra-TB-HPB-PIM.

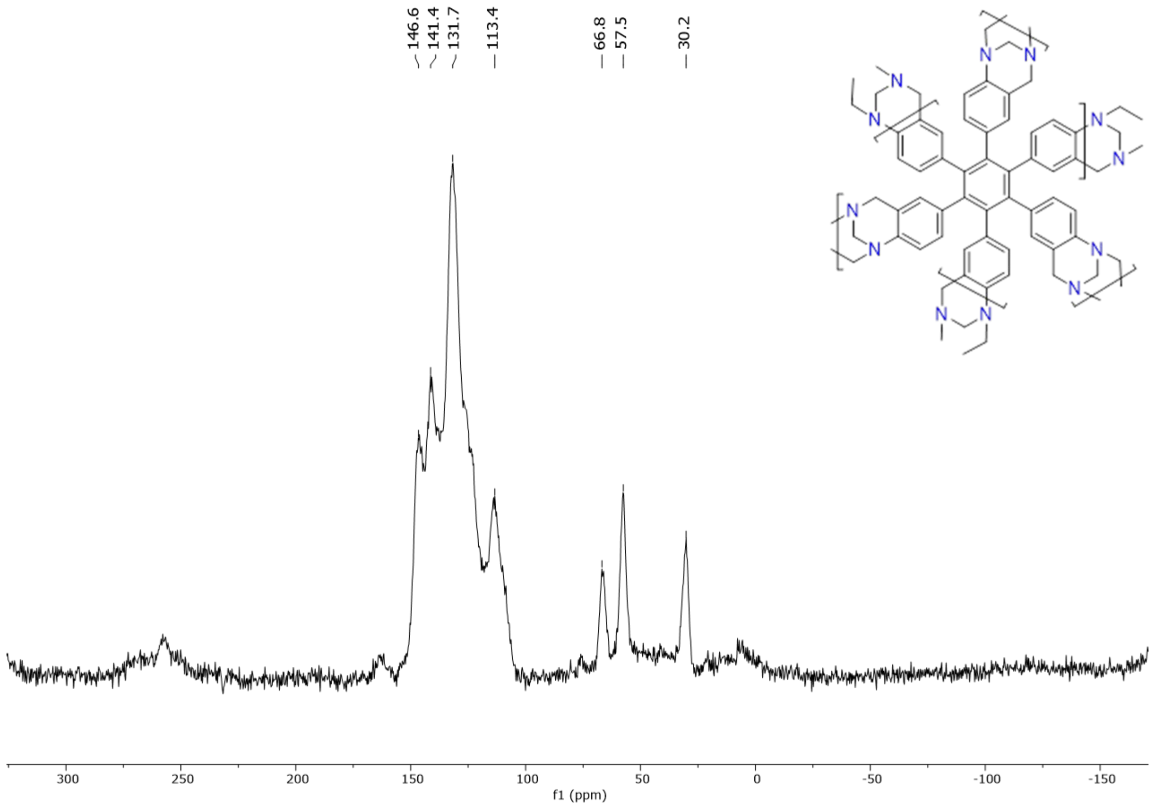

Figure S6. SSNMR for hexa-TB-HPB-PIM.

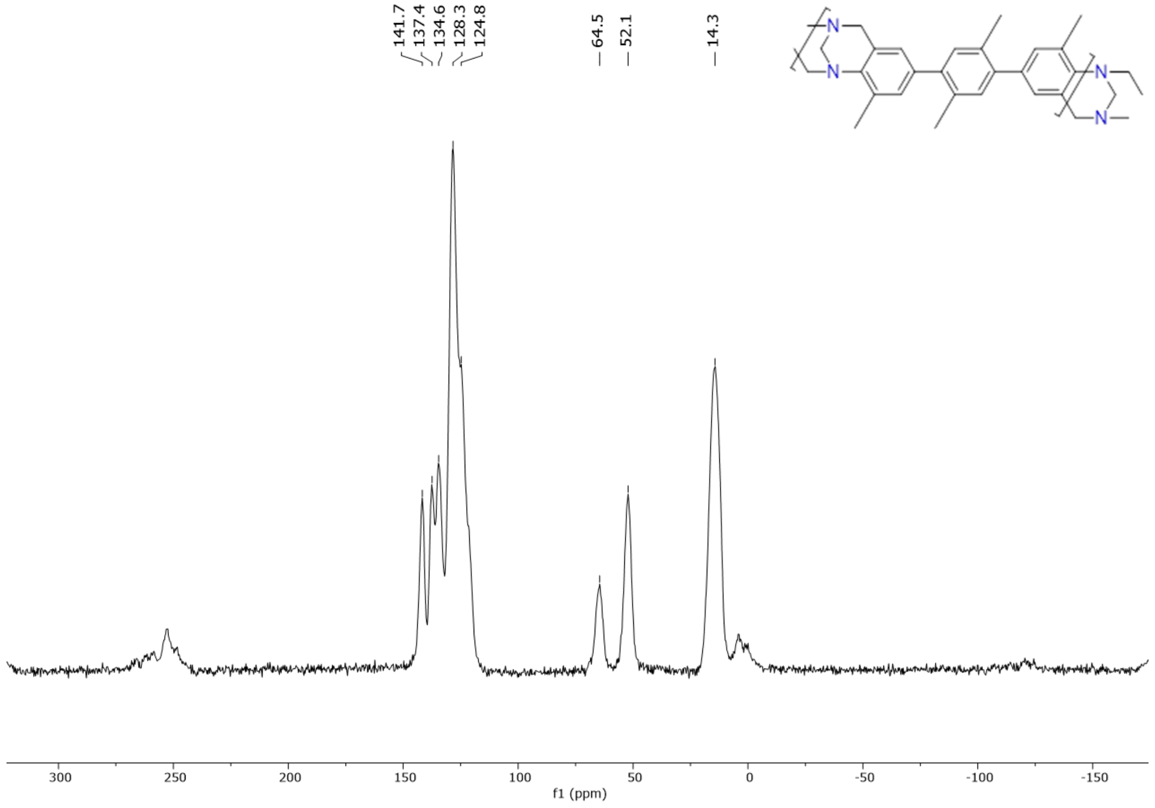

Figure S7. SSNMR for Linear TB-polymer 1.

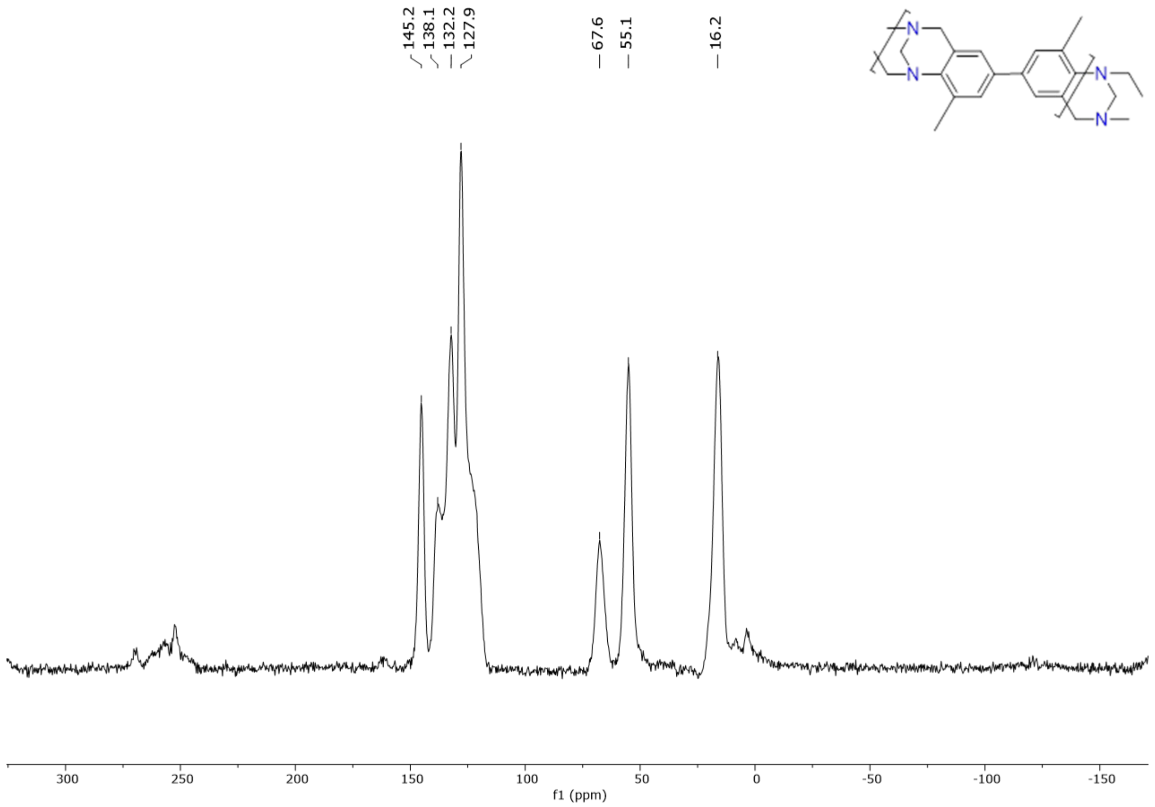

Figure S8. SSNMR for Linear TB-polymer 2.

FT-IR spectra

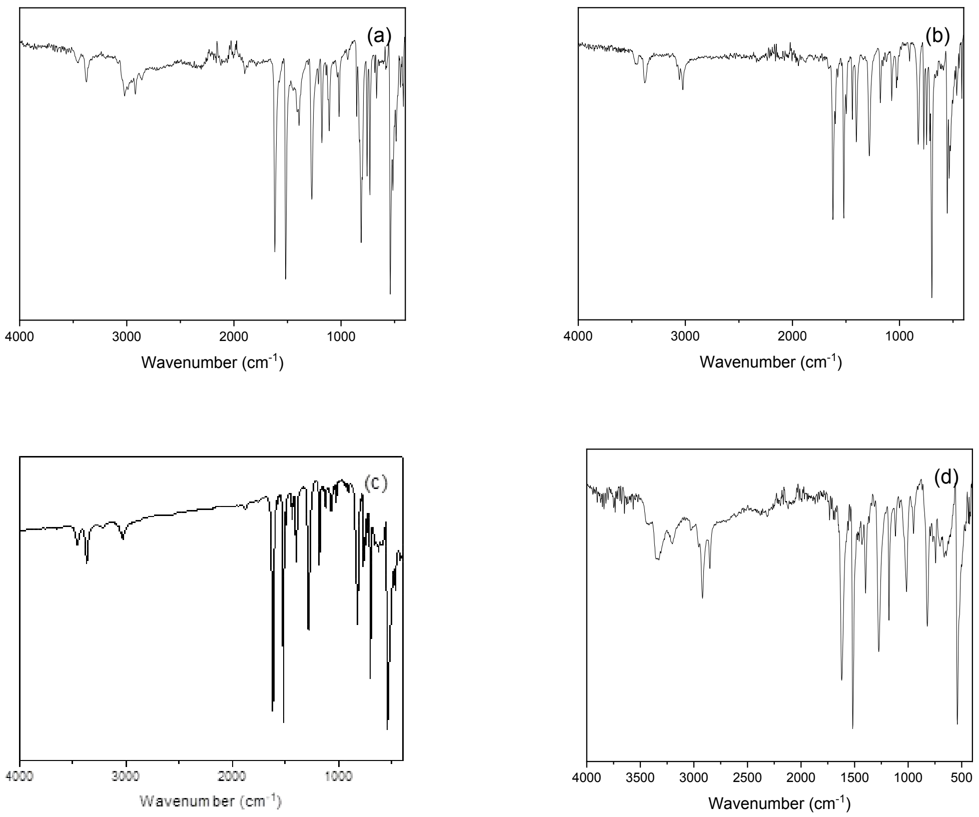

Figure S9. FT-IR for HPB monomers: (a) di-amino-Me-HPB, (b) di-amino-HPB, (c) tetra-amino-HPB, (d) hexa-amino-HPB.

Gas adsorption-desorption isotherms

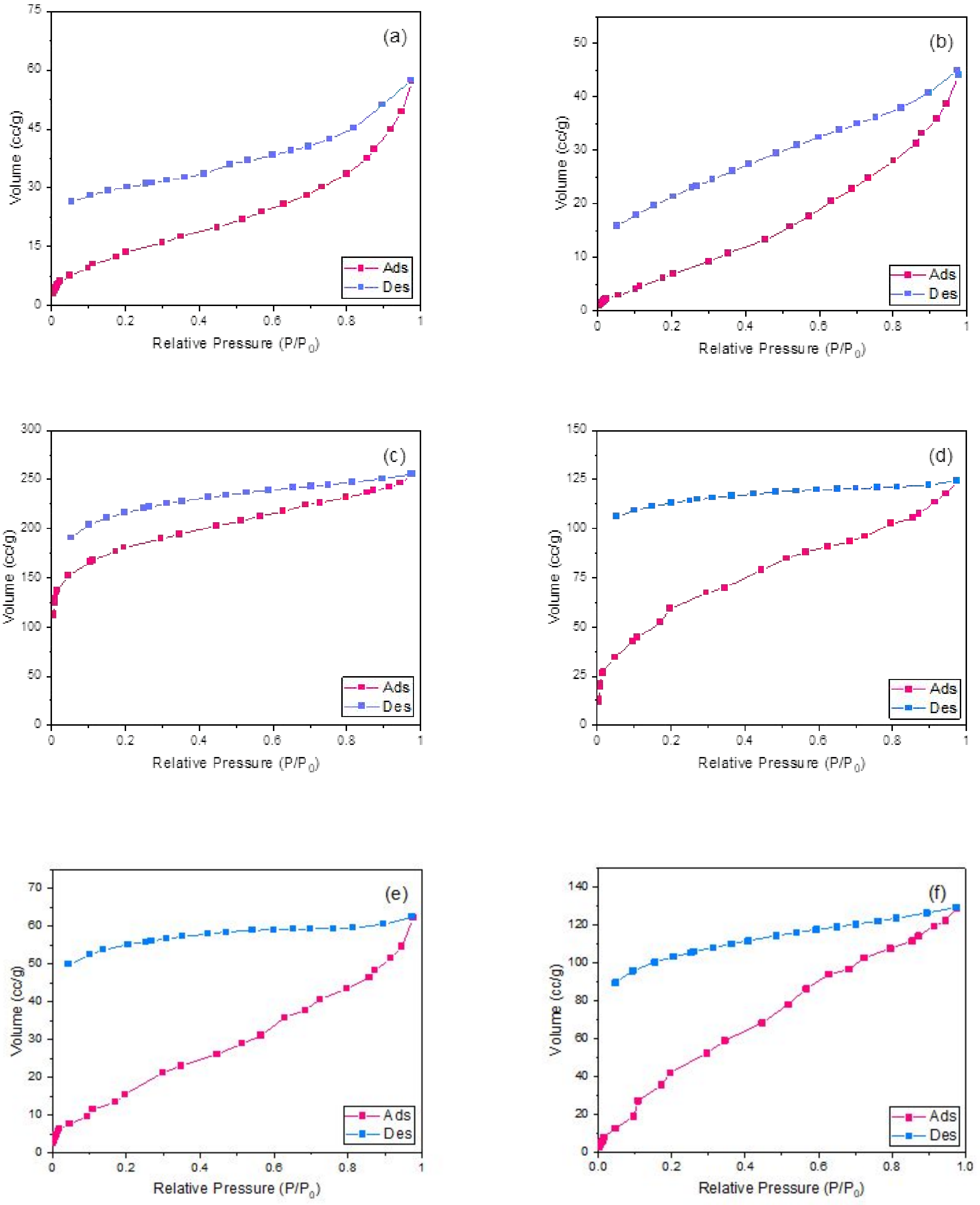

Figure S10. Nitrogen adsorption-desorption isotherms for TB-HPB-PIMs: (a) TB-Me-HPB-PIM, (b) TB-HPB-PIM, (c) tetra-TB-HPB-PIM, (d) hexa-TB-HPB-PIM, (e) Linear TB-polymer 1, (f) Linear TB-polymer 2.

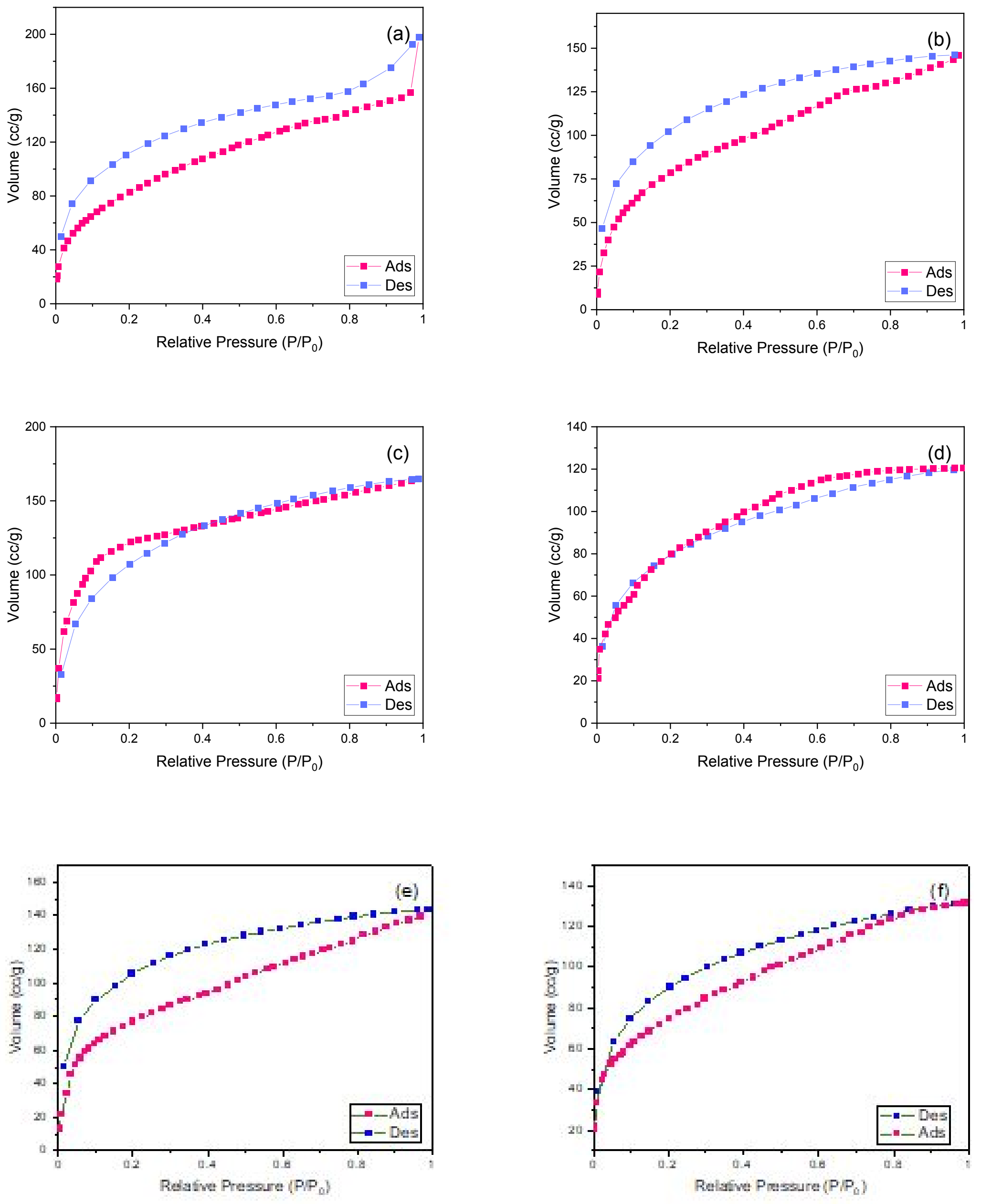

Figure S11. CO_2 adsorption-desorption isotherms at 195 K for TB-HPB-PIMs: (a) TB-Me-HPB-PIM, (b) TB-HPB-PIM, (c) tetra-TB-HPB-PIM, (d) hexa-TB-HPB-PIM (e) Linear TB-polymer 1, (f) Linear TB-polymer 2.

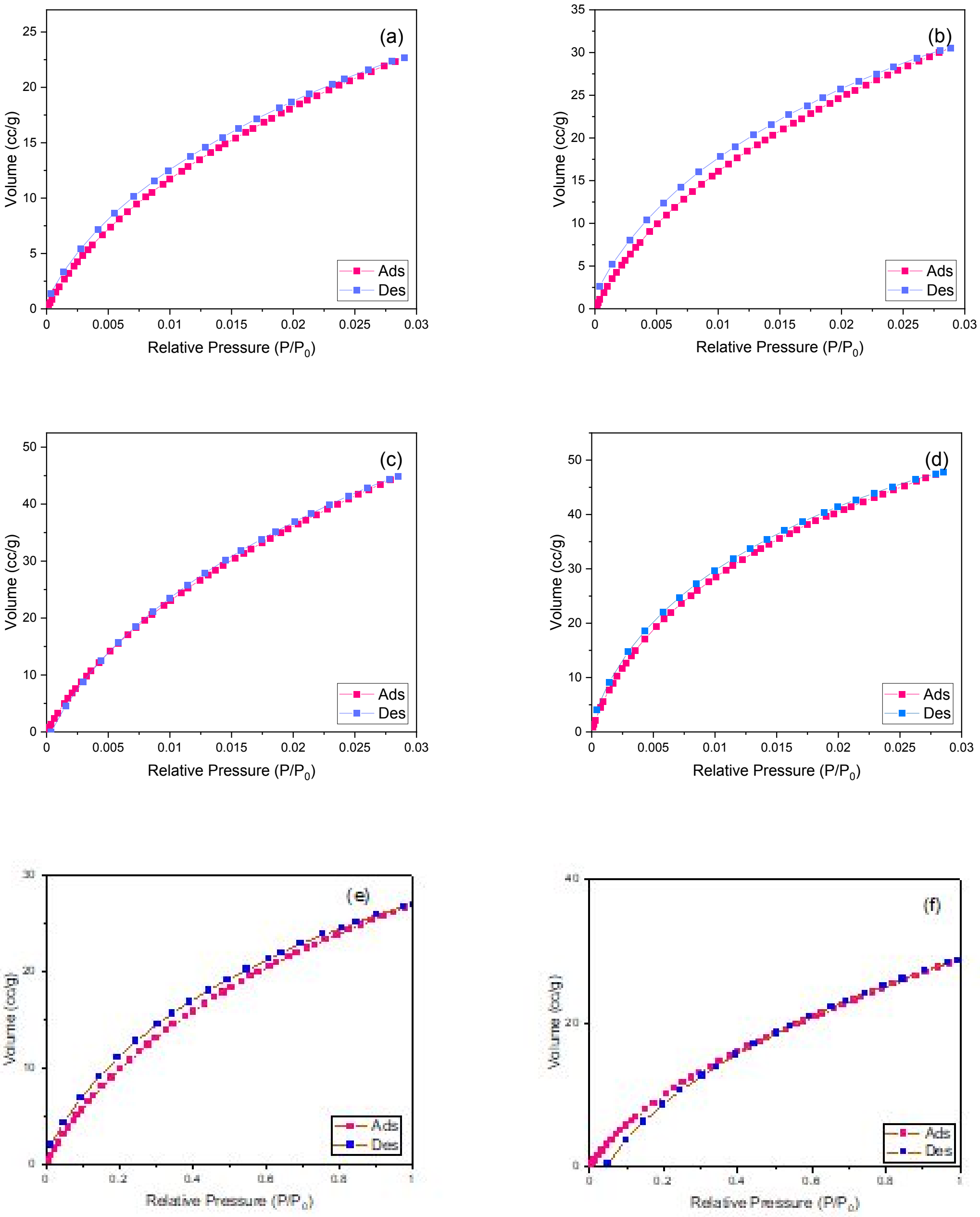

Figure S12. CO_2 adsorption-desorption isotherms at 273 K for TB-HPB-PIMs: (a) TB-Me-HPB-PIM, (b) TB-HPB-PIM, (c) tetra-TB-HPB-PIM, (d) hexa-TB-HPB-PIM, (e) Linear TB-polymer 1, (f) Linear TB-polymer 2.

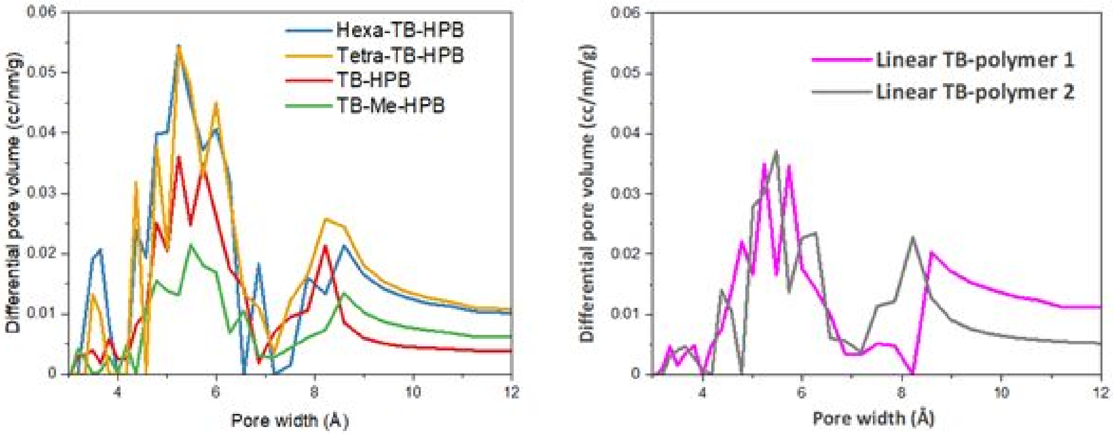

Figure S13. Pore size distribution of TB-HPB, Linear TB-polymer 1 and Linear TB-polymer 2 calculated from CO_2 adsorption at 273 K using the NLDFT model.

TGA and DTG

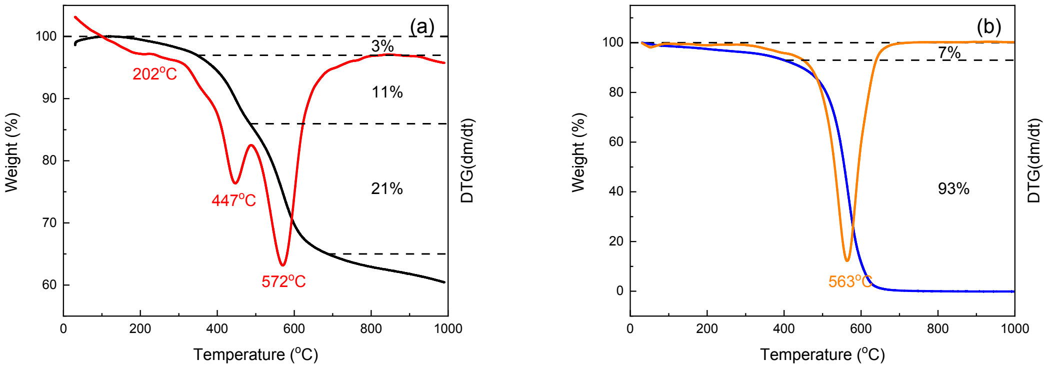

Figure S14. TB-Me-HPB-PIM: (a) TGA and DTG under nitrogen, (b) TGA and DTG under air.

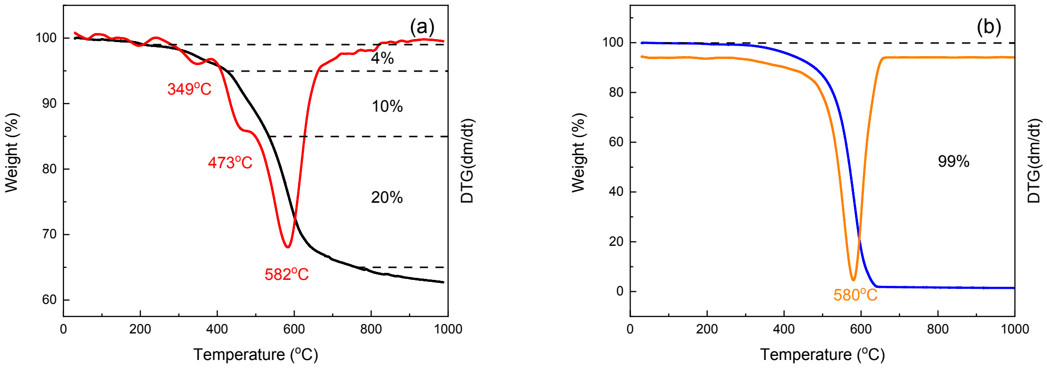

Figure S15. TB-HPB-PIM: (a) TGA and DTG under nitrogen, (b) TGA and DTG under air.

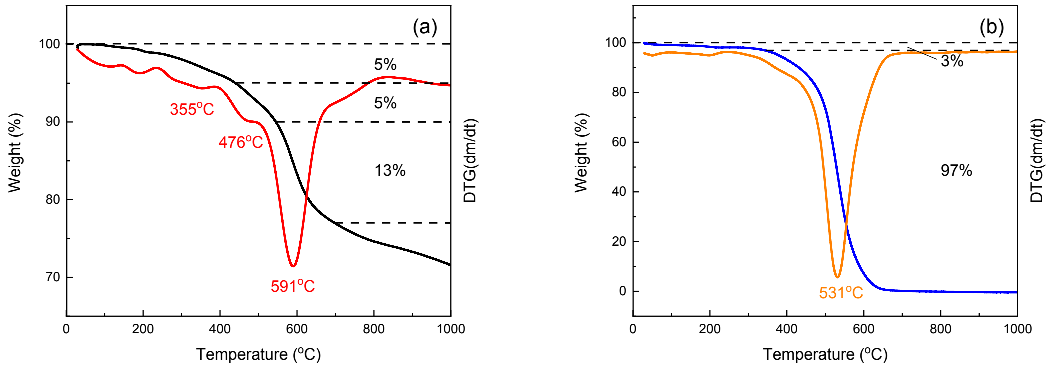

Figure S16. Tetra-TB-HPB-PIM: (a) TGA and DTG under nitrogen, (b) TGA and DTG under air.

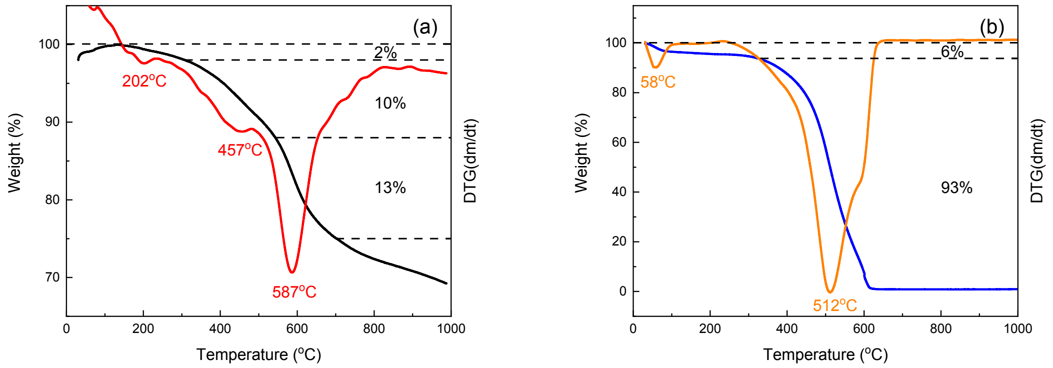

Figure S17. Hexa-TB-HPB-PIM: (a) TGA and DTG under nitrogen, (b) TGA and DTG under air.

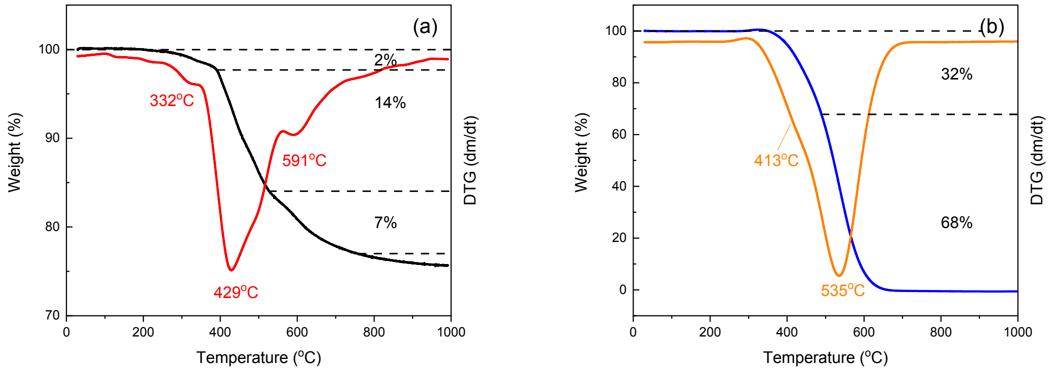

Figure S18. Linear TB-polymer 1: (a) TGA and DTG under nitrogen, (b) TGA and DTG under air.

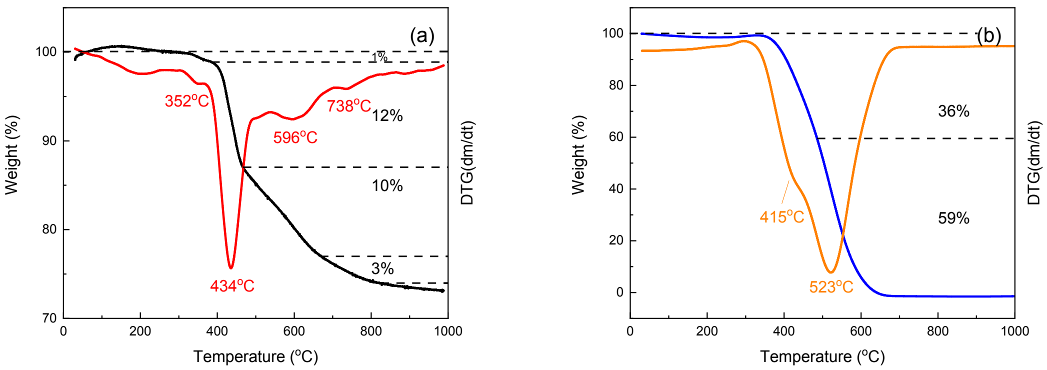

Figure S19. Linear TB-polymer 2: (a) TGA and DTG under nitrogen, (b) TGA and DTG under air.

SEM images

(a)

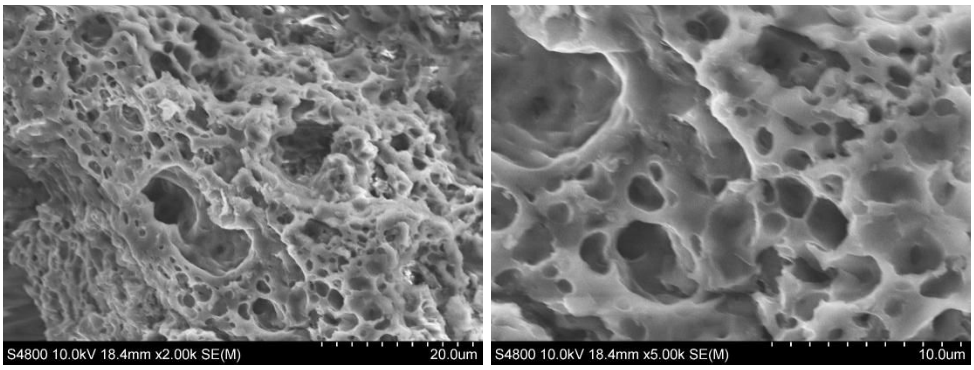

(b)

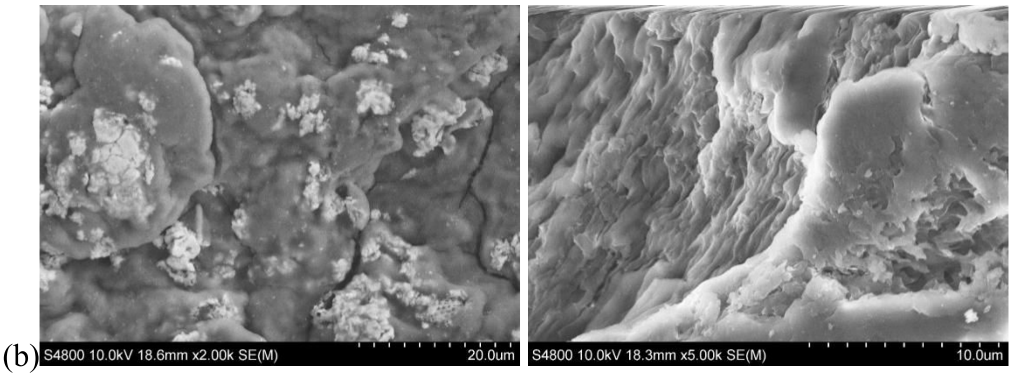

(c)

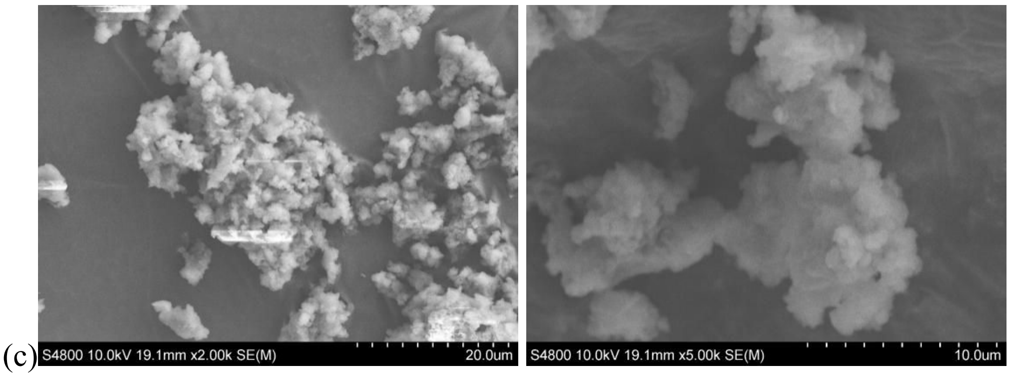

(d) 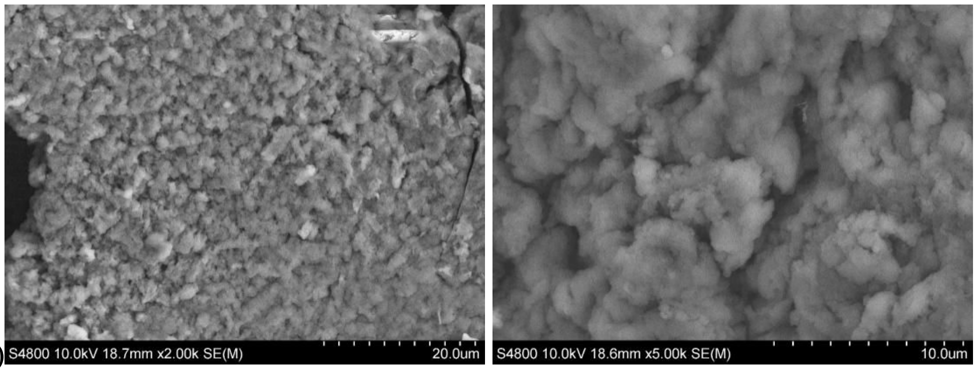

Figure S20. SEM of TB-HPB-PIMs: (a) TB-tetra-Me-HPB, (b) TB-HPB, (c) tetra-TB-HPB, (d) hexa-TB-HPB.

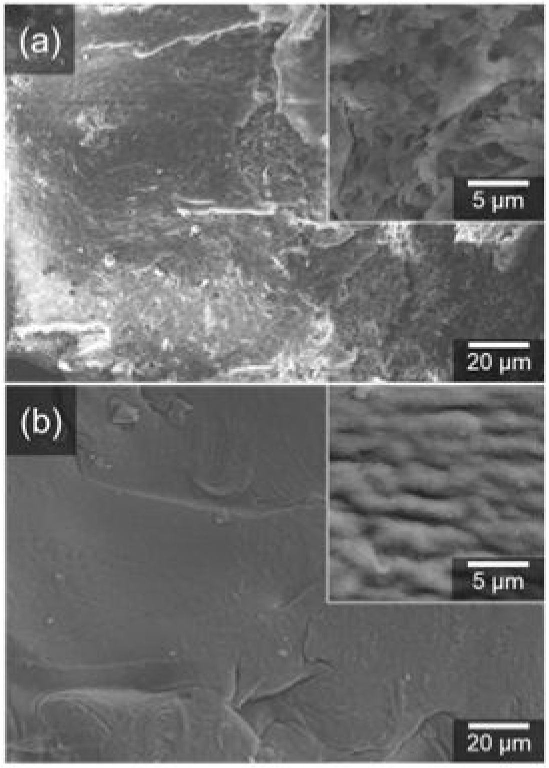

Figure S21. SEM of linear TB-polymers: (a) linear TB-polymer 1, (b) linear TB-polymer 2.

^{1}H and ^{13} C NMR spectra of precursors and monomers

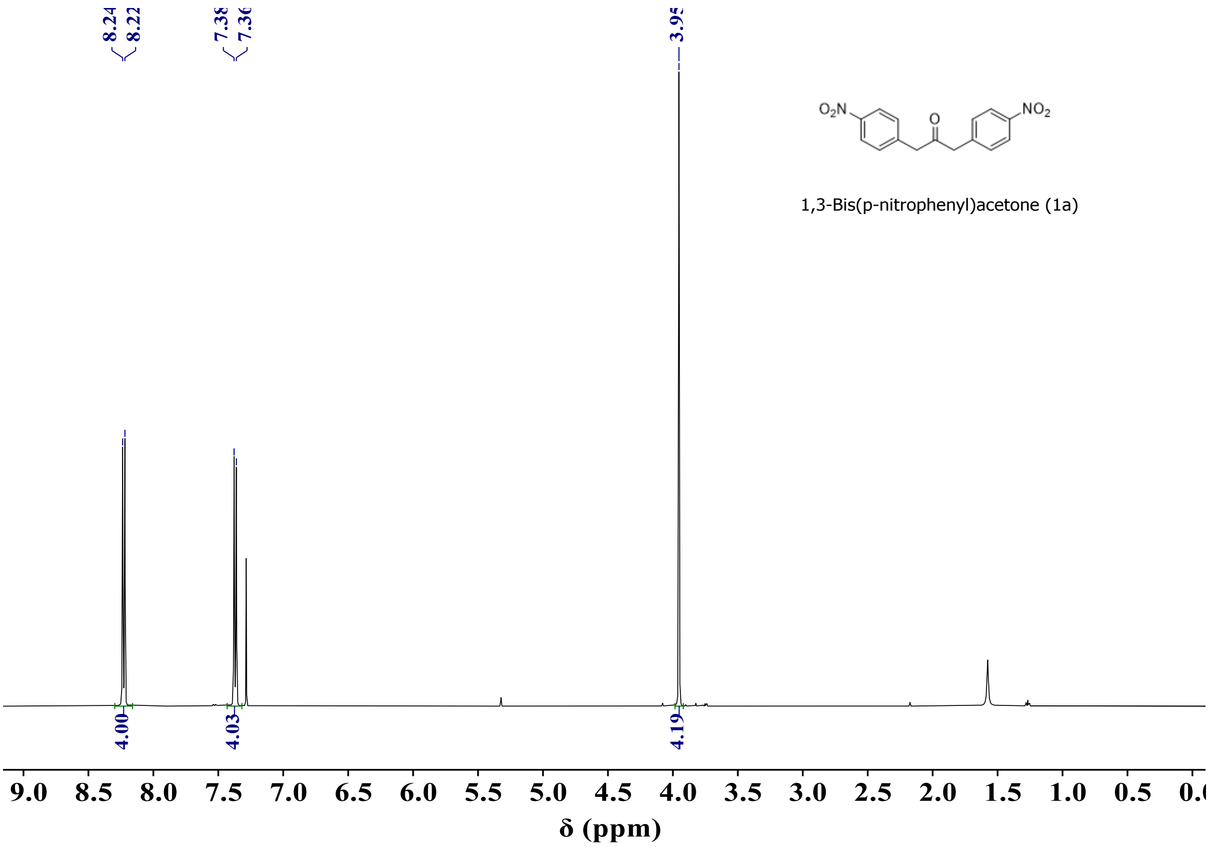

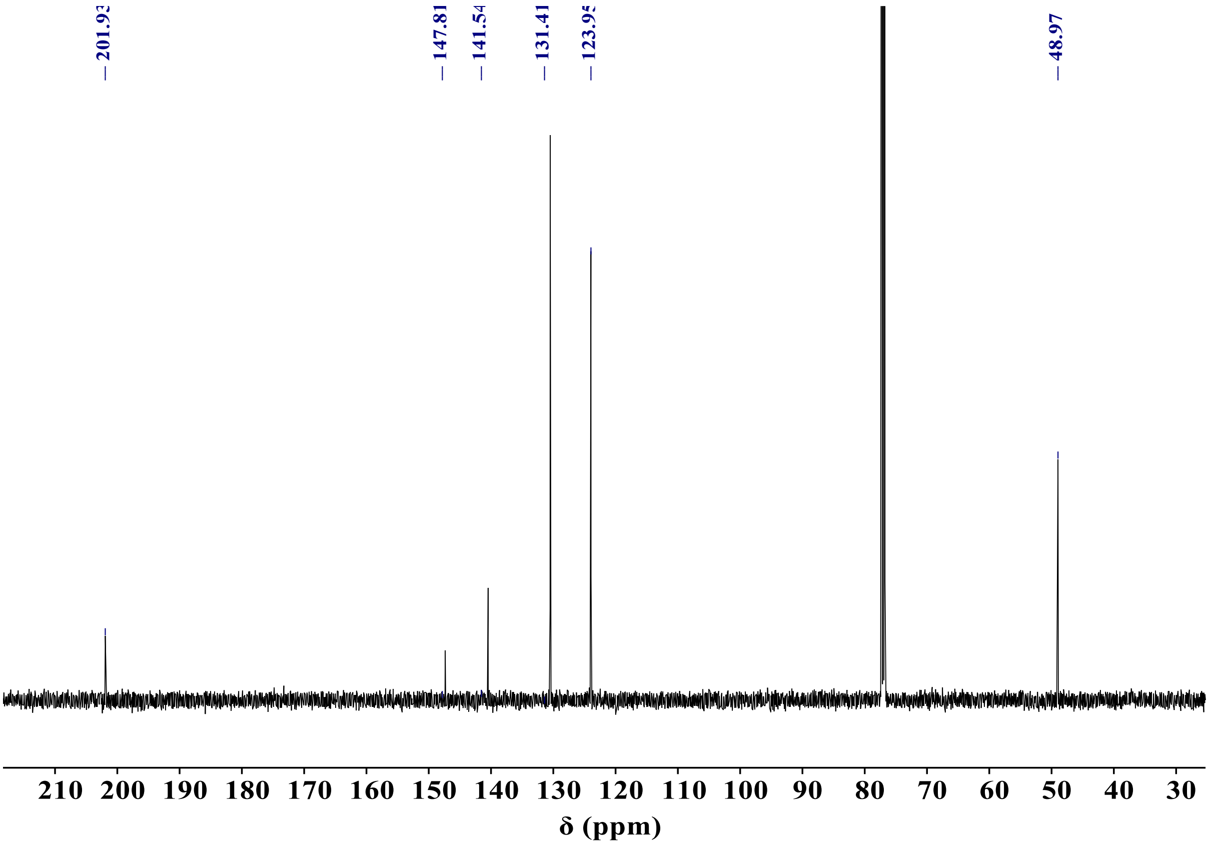

Figure S22. ^{1}H and ^{13} C NMR of 1,3-bis(*p*-nitrophenyl)acetone (1a)

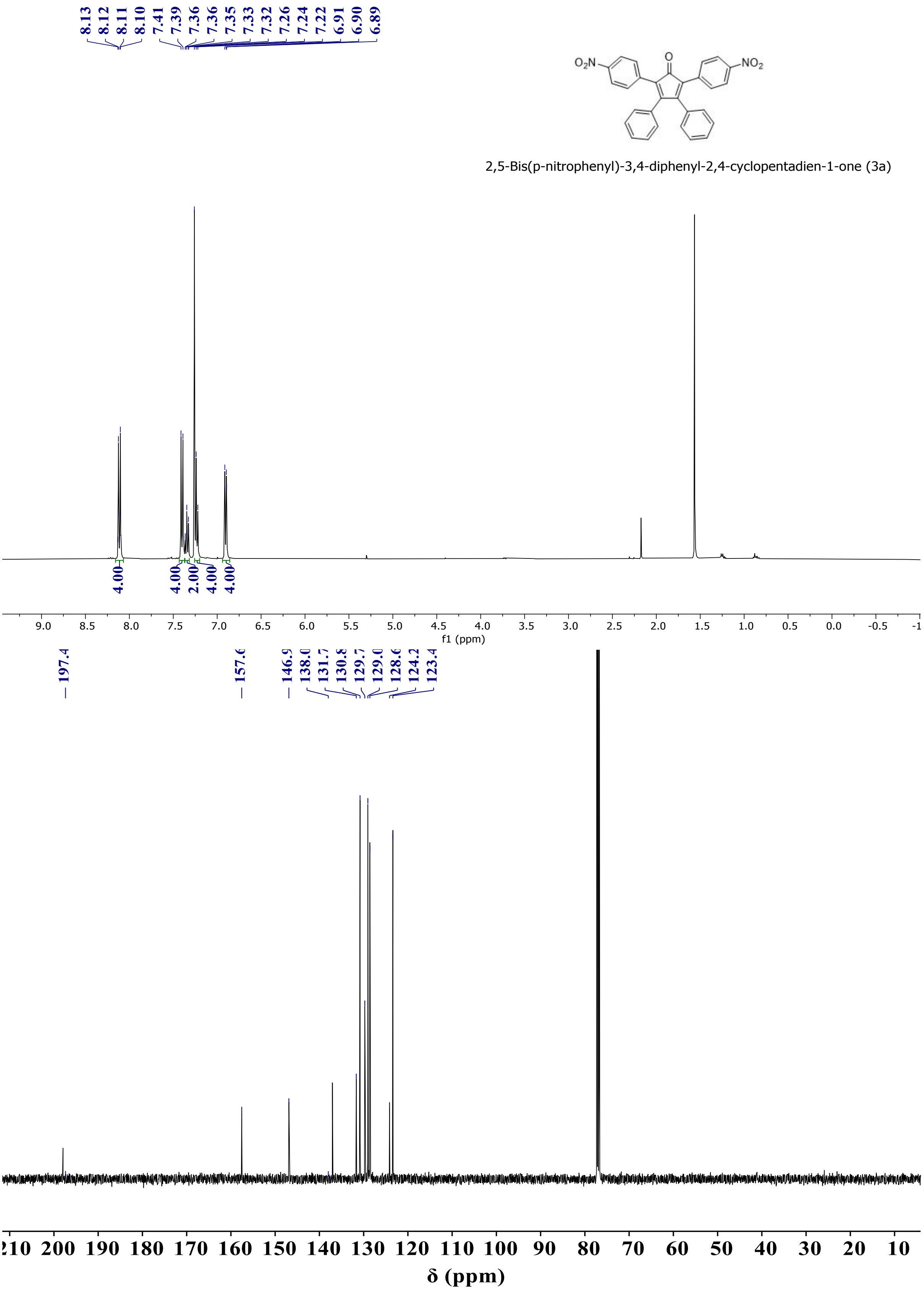

Figure S23. ^{1}H and ^{13}C NMR of 2,5-bis(p-nitrophenyl)-3,4-diphenyl-2,4-cyclopentadien-1-one (3a)

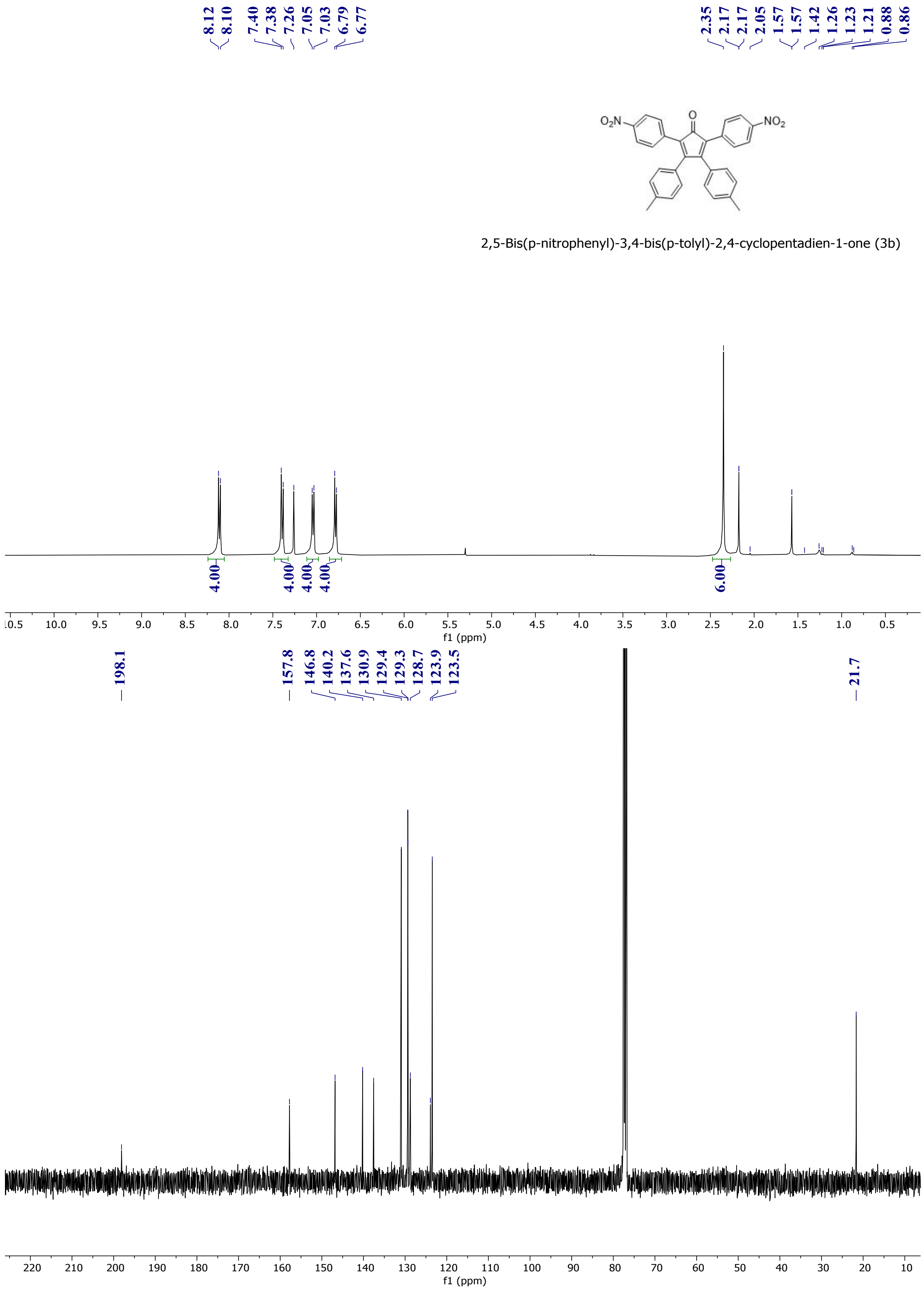

Figure S24. ^{1}H and ^{13}C NMR of 2,5-bis(p-nitrophenyl)-3,4-bis(p-tolyl)-2,4-cyclopentadien-1-one (3b)

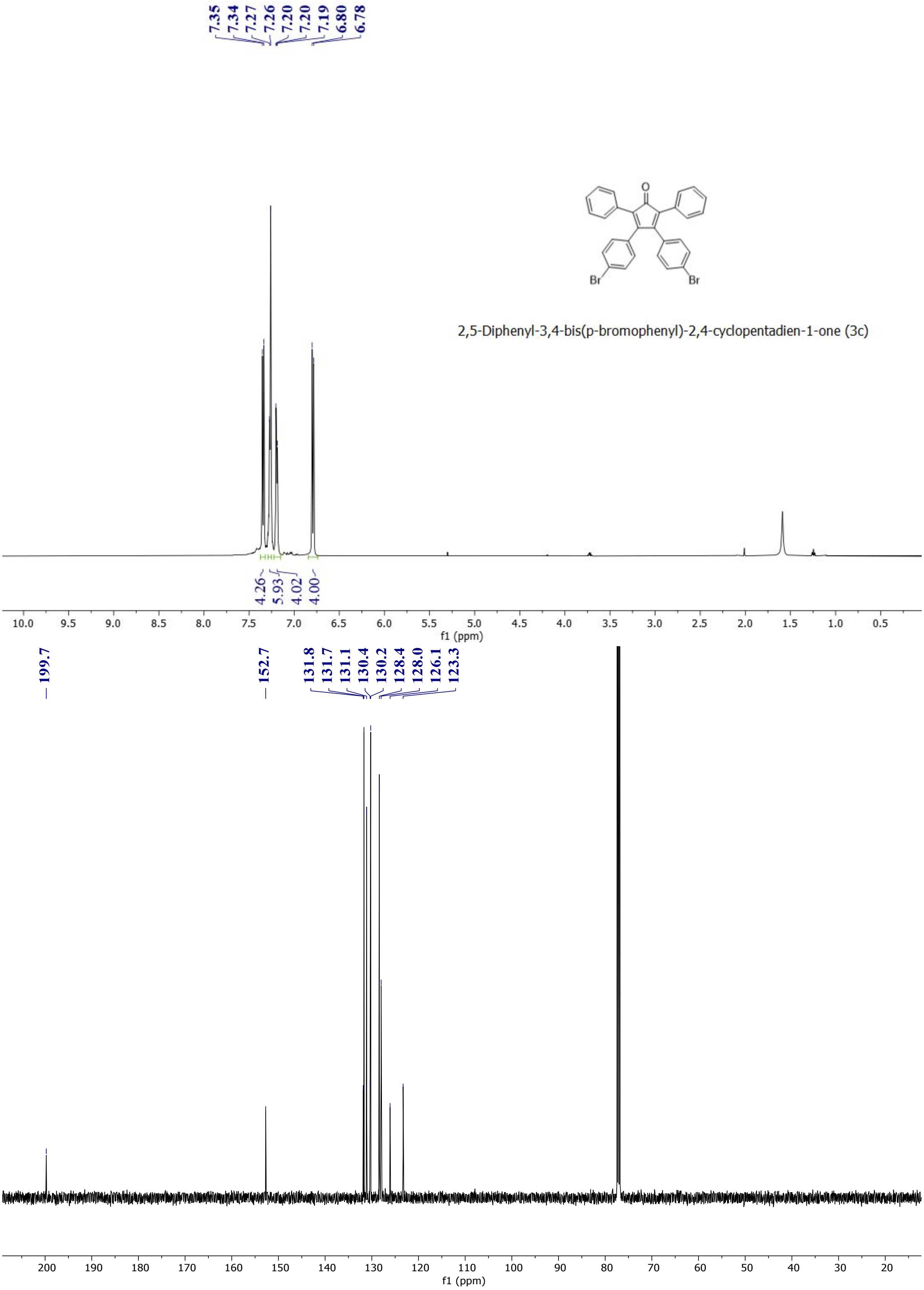

Figure S25. ^{1}H and ^{13}C NMR of 2,5-diphenyl-3,4-bis(p-bromophenyl)-2,4-cyclopentadien-1-one (3c)

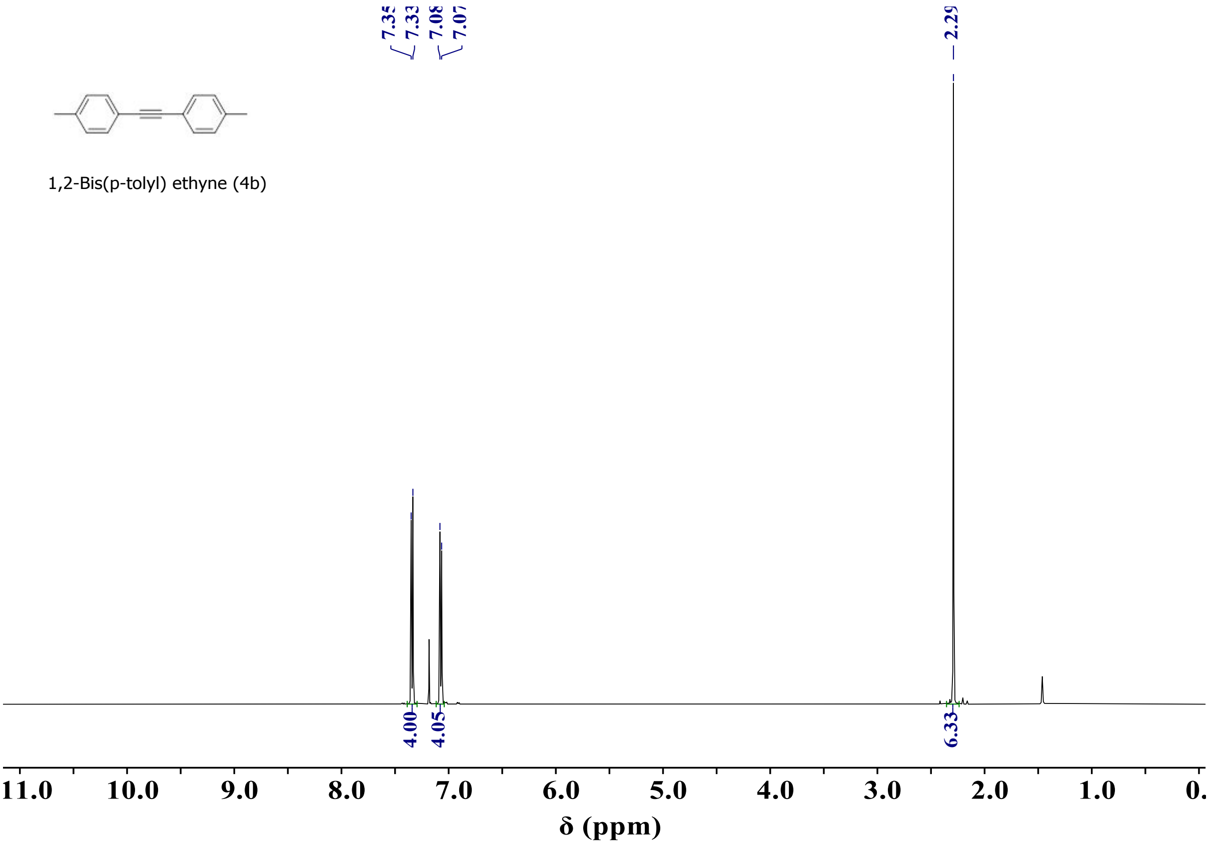

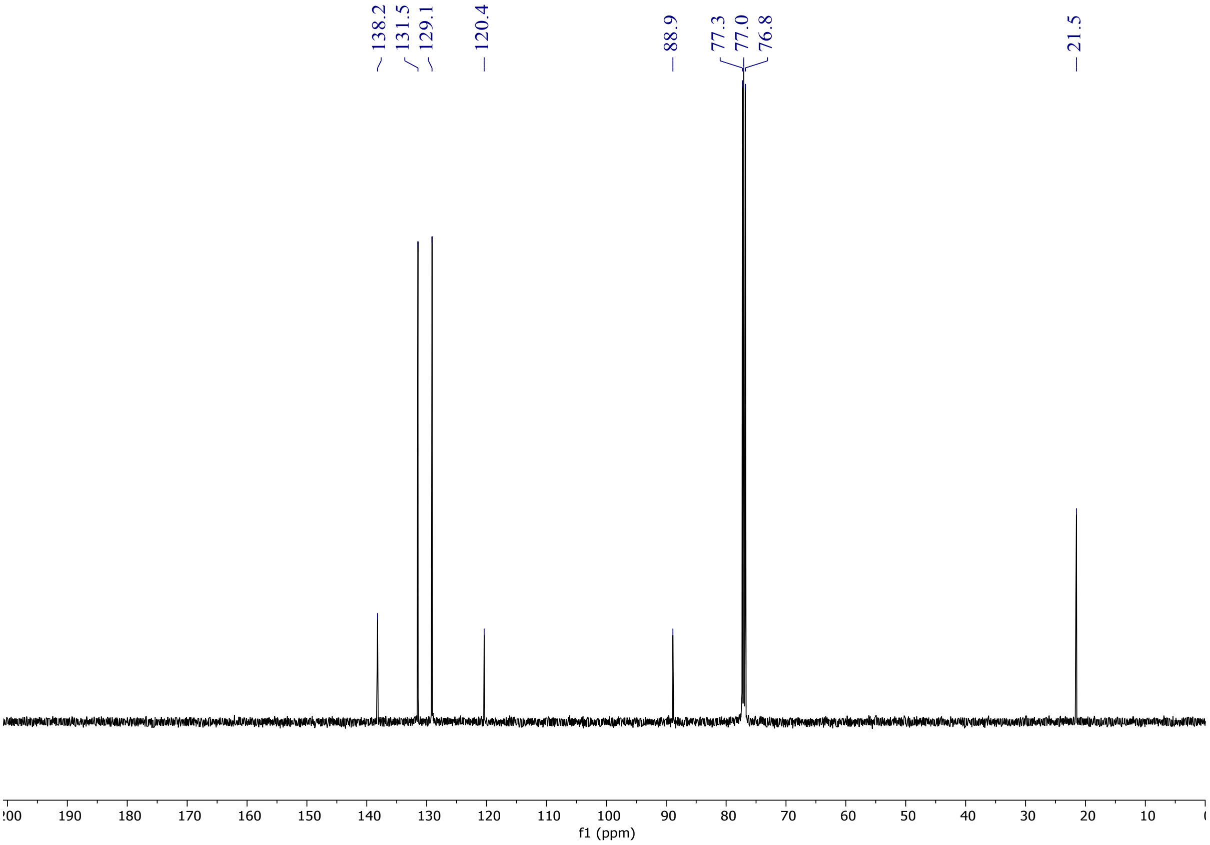

Figure S26. ^{1}H and ^{13}C NMR of 1,2-bis(p-tolyl) ethyne (4b)

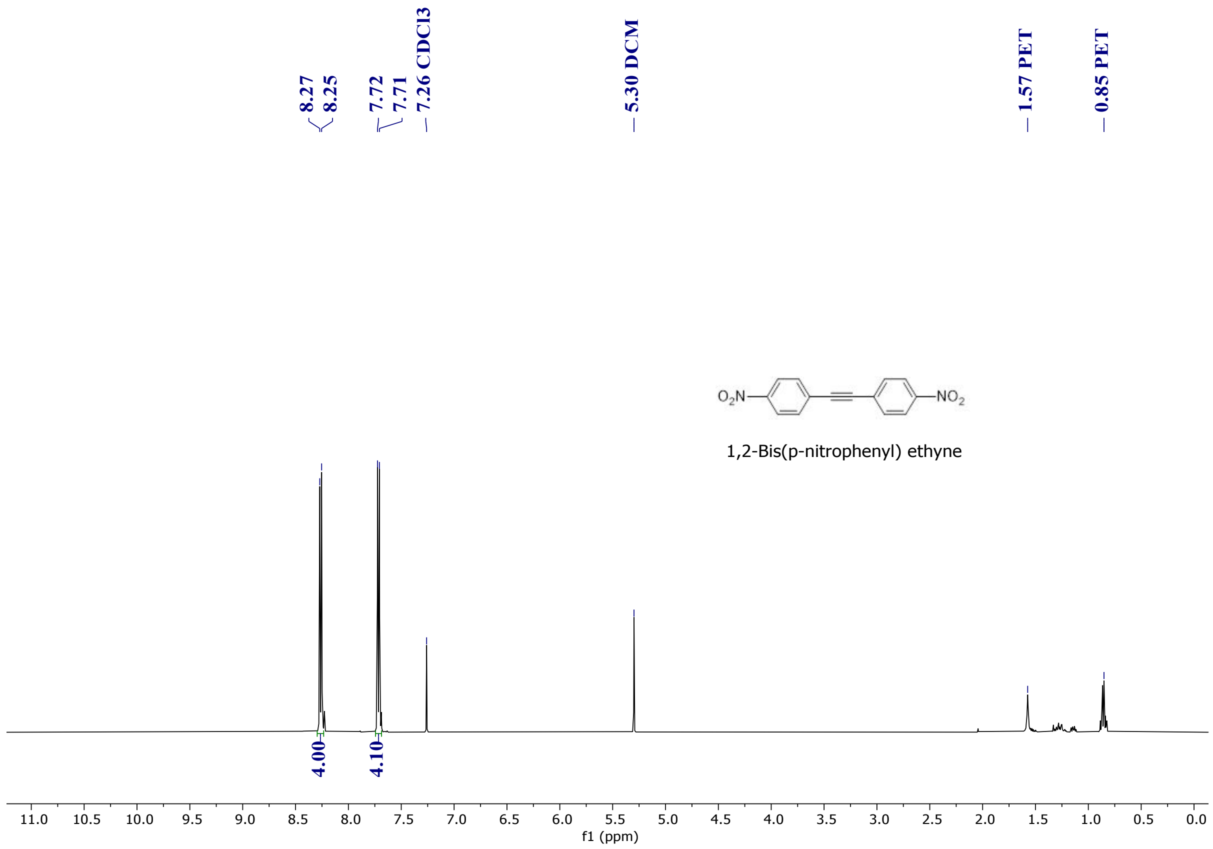

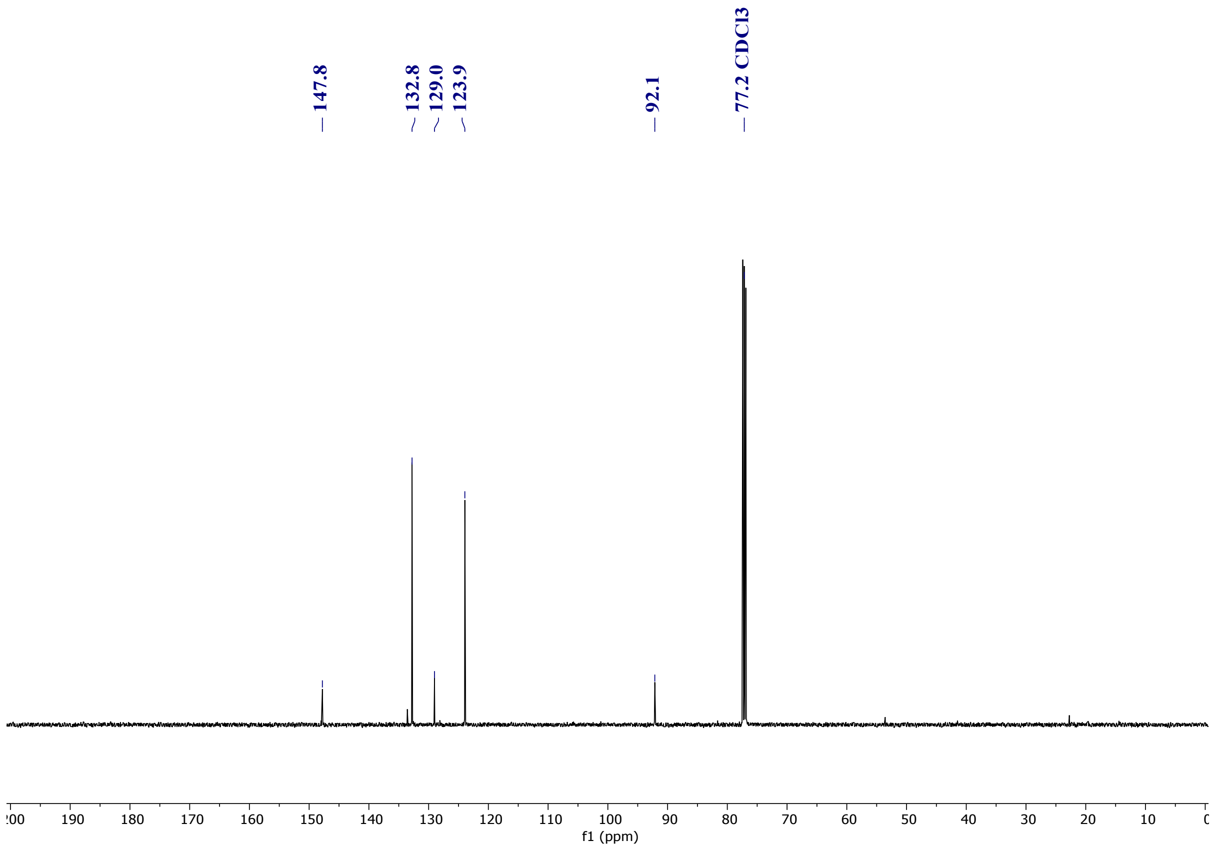

Figure S27. ^{1}H and ^{13}C NMR of 1,2-bis(p-nitrophenyl) ethyne

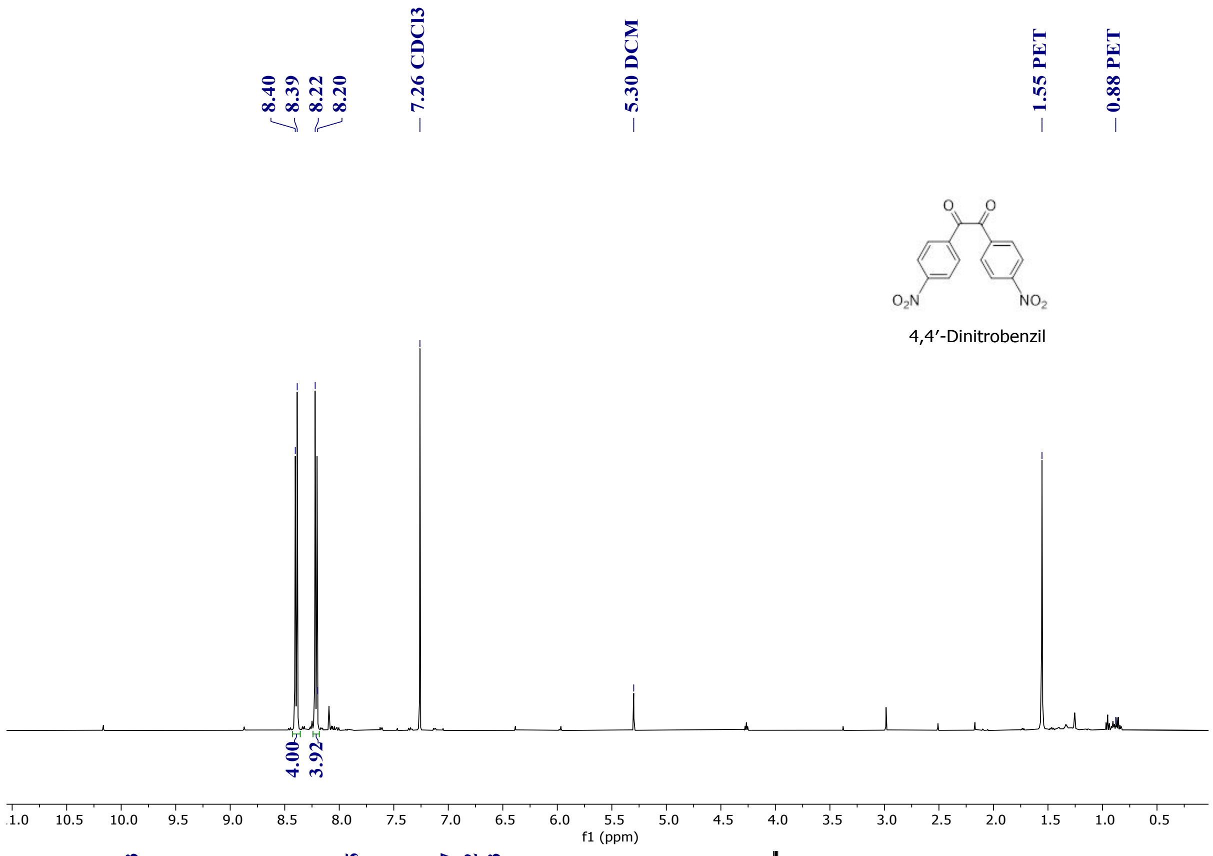

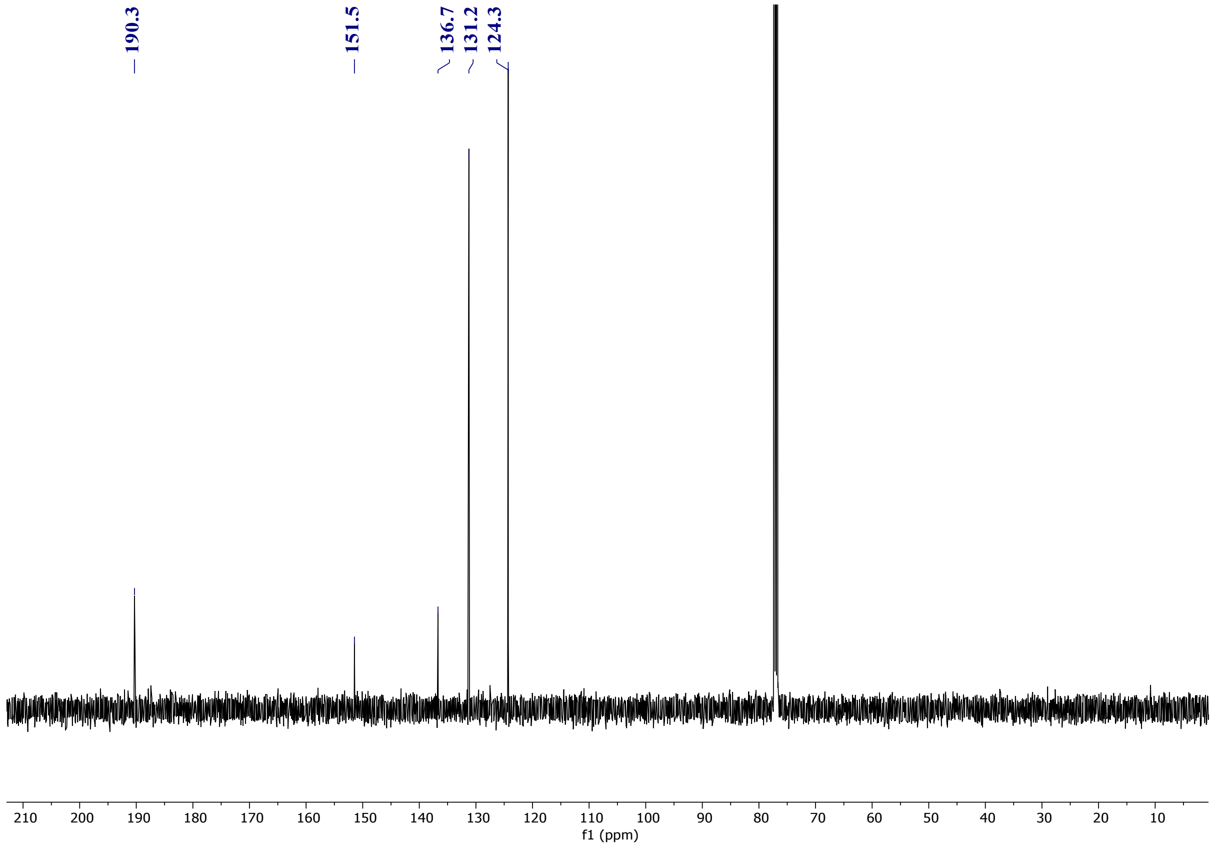

Figure S28. ^{1}H and ^{13} C NMR of 4,4′-dinitrobenzil

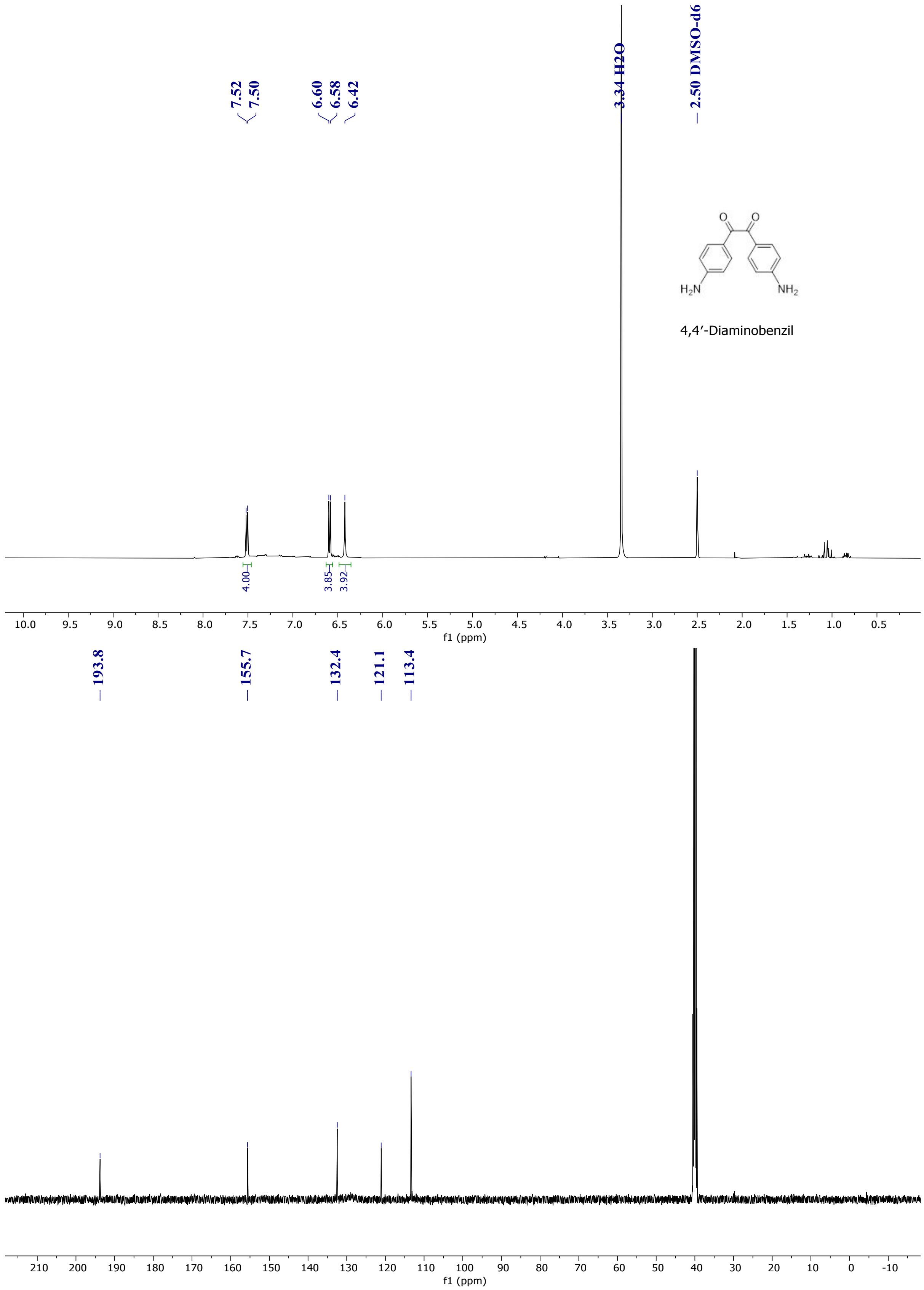

Figure S29. ^{1}H and ^{13}C NMR of 4,4′-diaminobenzil

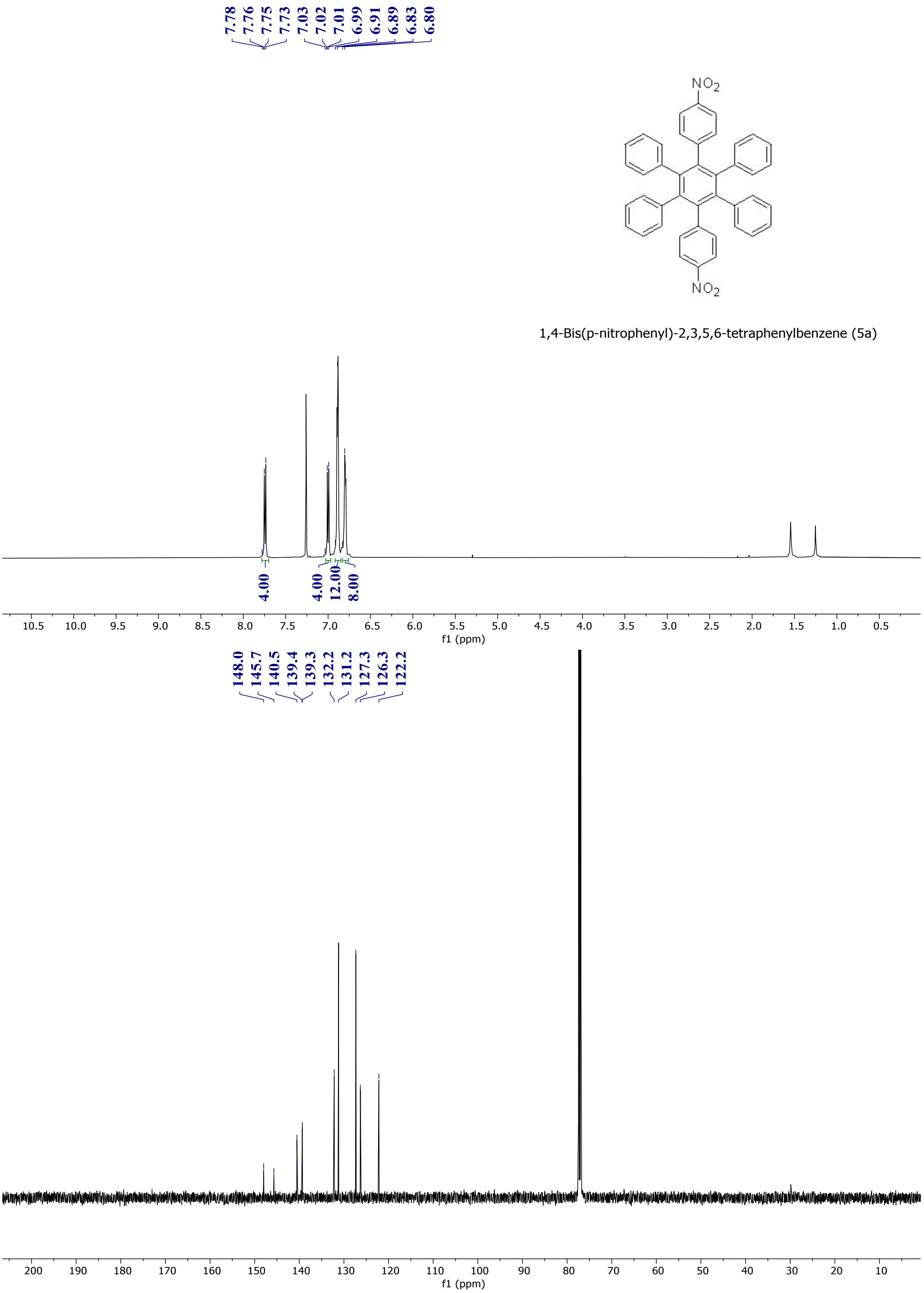

Figure S30. ^{1}H and ^{13}C NMR of 1,4-bis(p-nitrophenyl)-2,3,5,6-tetraphenylbenzene

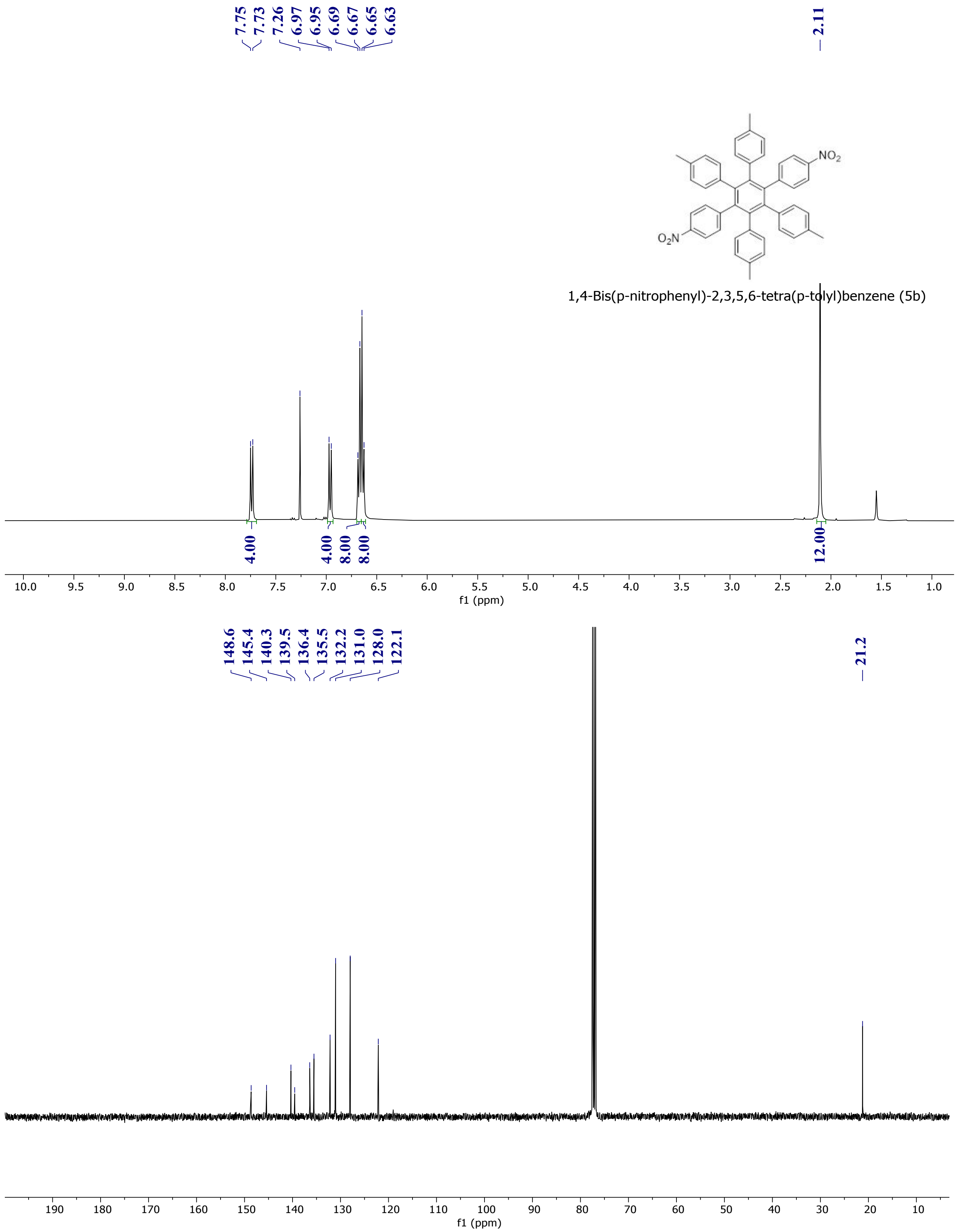

Figure S31. ^{1}H and ^{13}C NMR of 1,4-bis(p-nitrophenyl)-2,3,5,6-tetra(p-tolyl)benzene (5b)

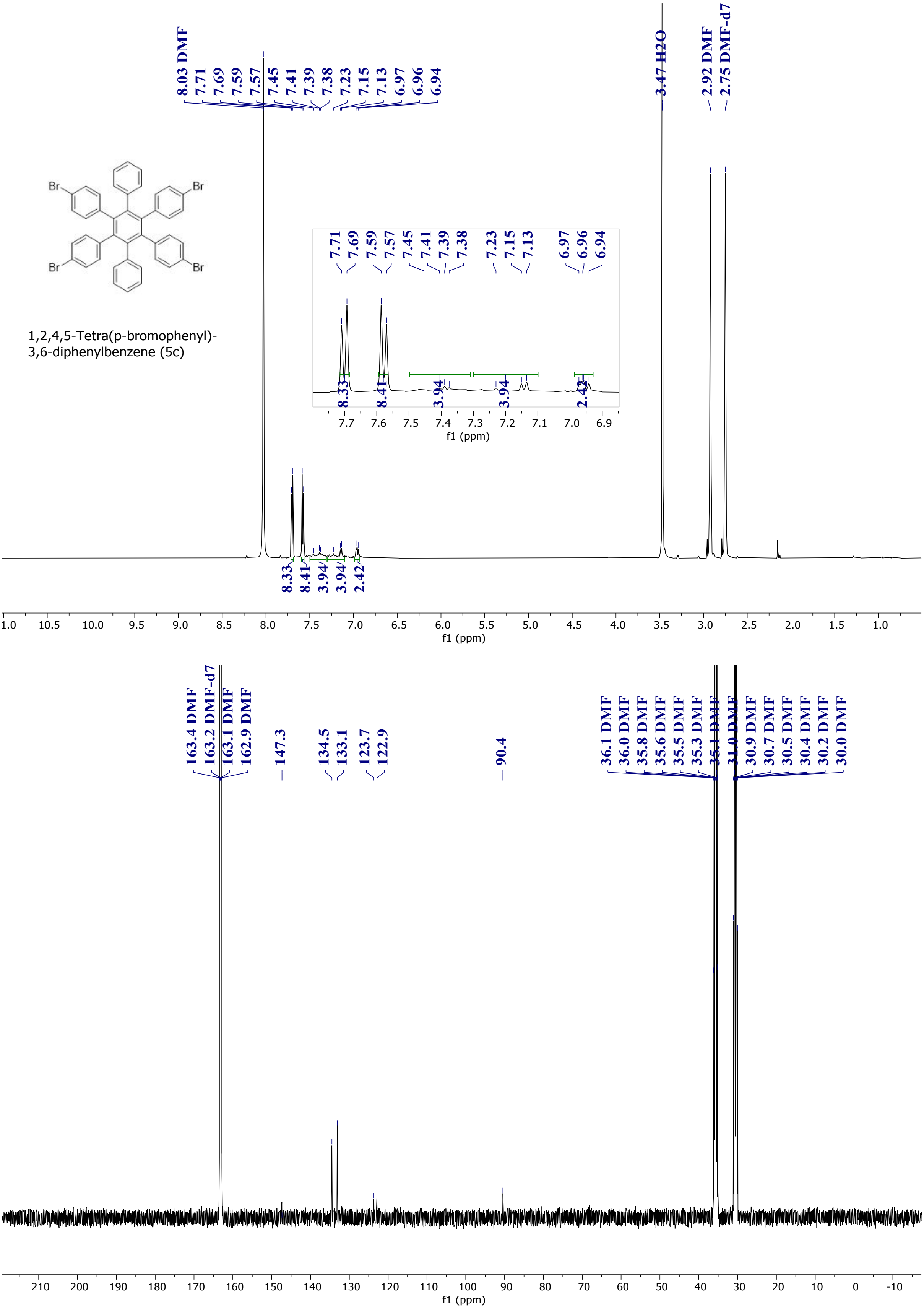

Figure S32. ^{1}H and ^{13}C NMR of 1,2,4,5-tetra(p-bromophenyl)-3,6-diphenylbenzene (5c). Inset shows peaks details

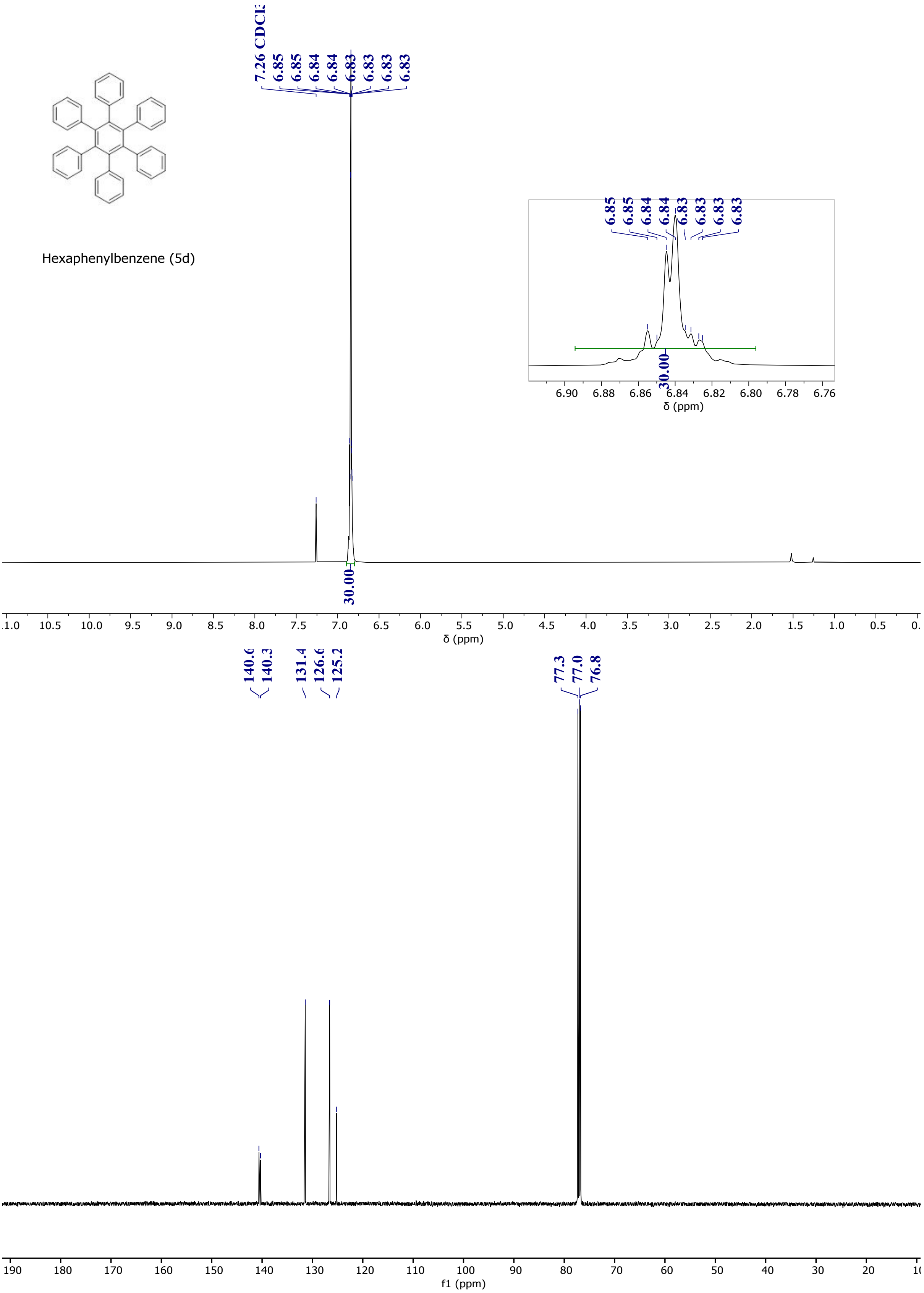

Figure S33. ^{1}H and ^{13}C NMR of hexaphenylbenzene (5d). Inset shows peaks details.

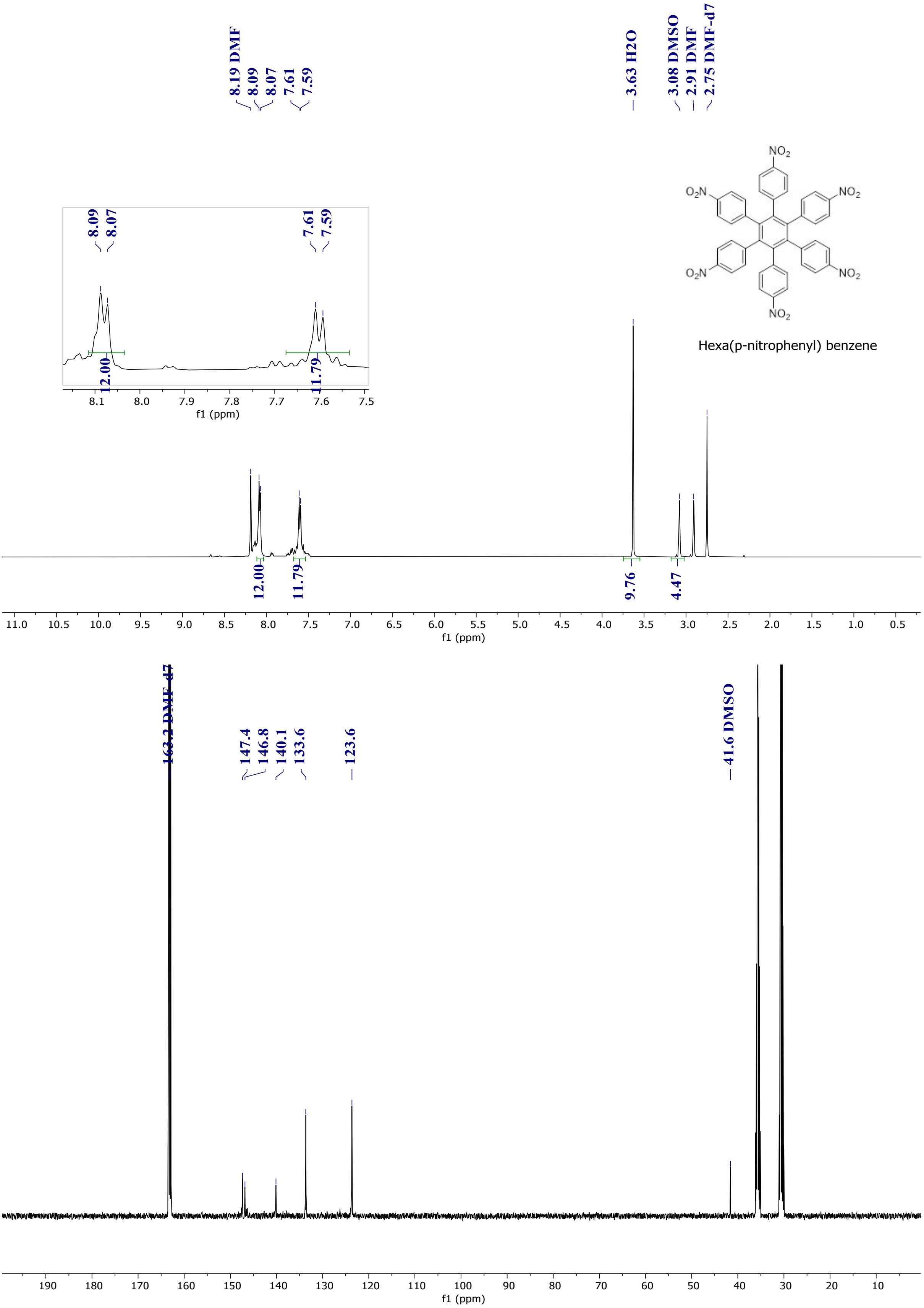

Figure S34. ^{1}H and ^{13}C NMR of hexa(p-nitrophenyl) benzene. Inset shows peaks details

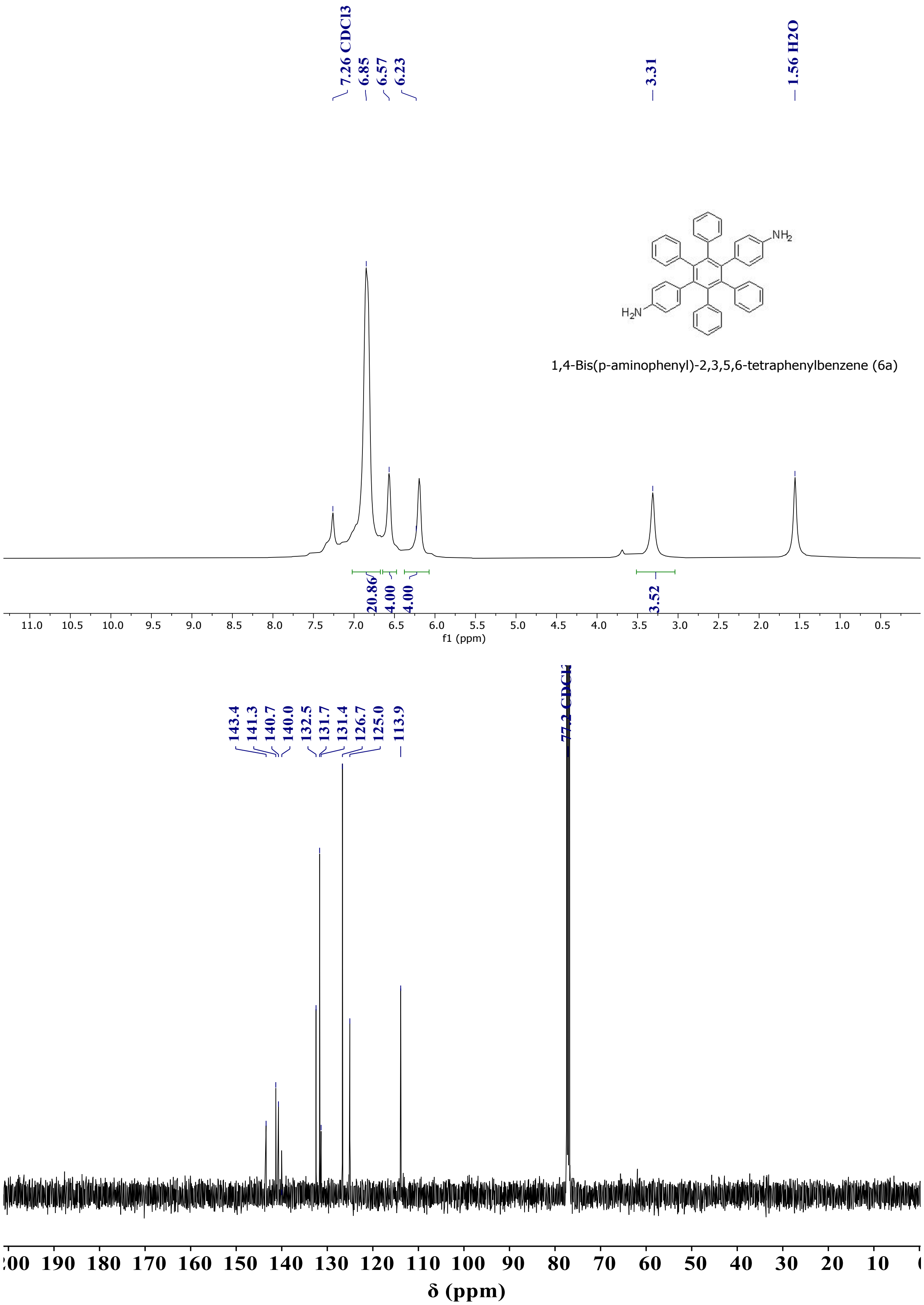

Figure S35. ^{1}H and ^{13}C NMR of 1,4-bis(p-aminophenyl)-2,3,5,6-tetraphenylbenzene (6a).

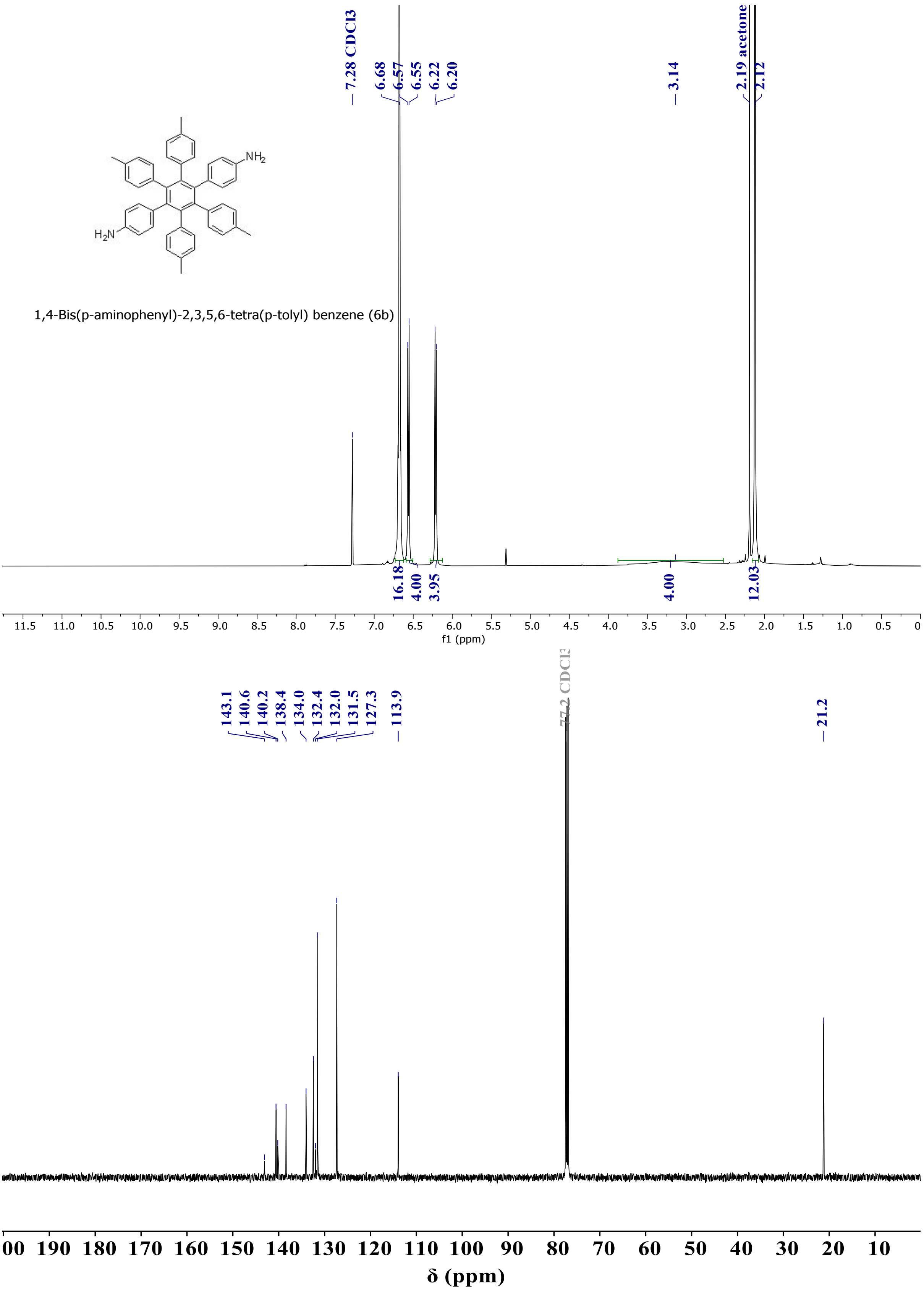

Figure S36. ^{1}H and ^{13}C NMR of 1,4-bis(p-aminophenyl)-2,3,5,6-tetra(p-tolyl) benzene (6b)

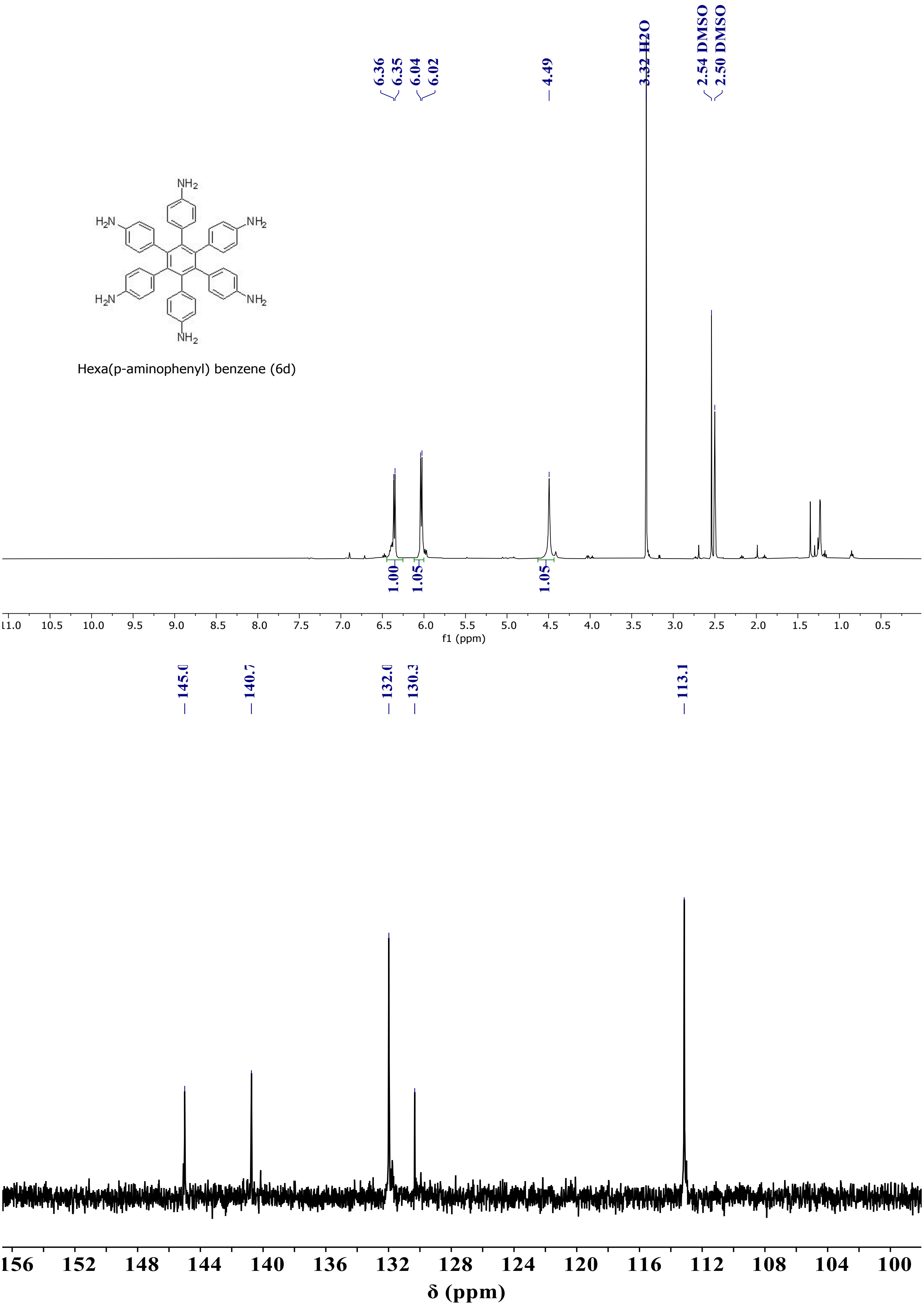

Figure S37. ^{1}H and ^{13}C NMR of hexa(p-aminophenyl) benzene ($R=NH_2$) (6d)

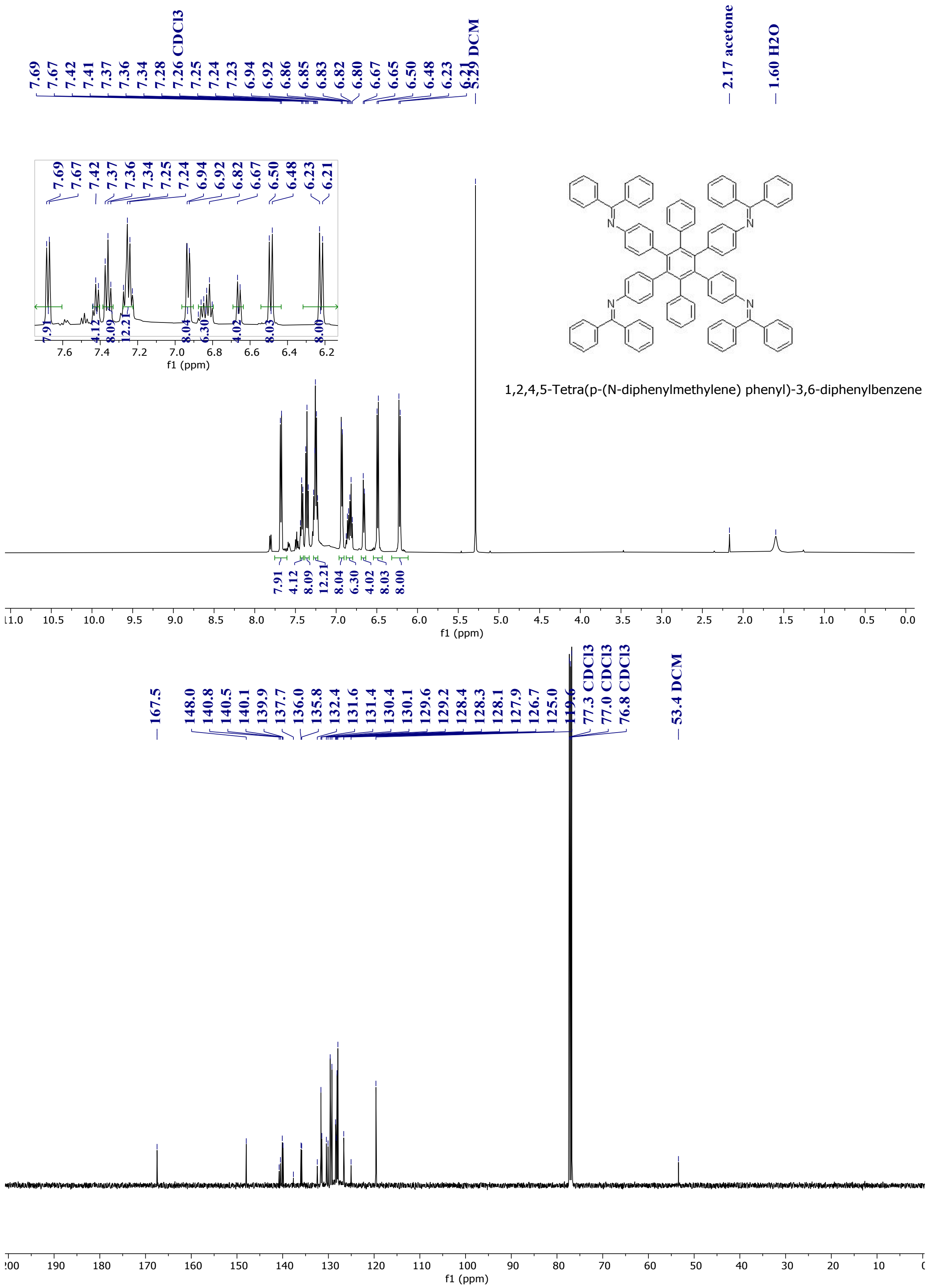

Figure S38. ^{1}H and ^{13}C NMR of 1,2,4,5-tetra(p-(N-diphenylmethylene) phenyl)-3,6-diphenylbenzene. Inset shows peaks details

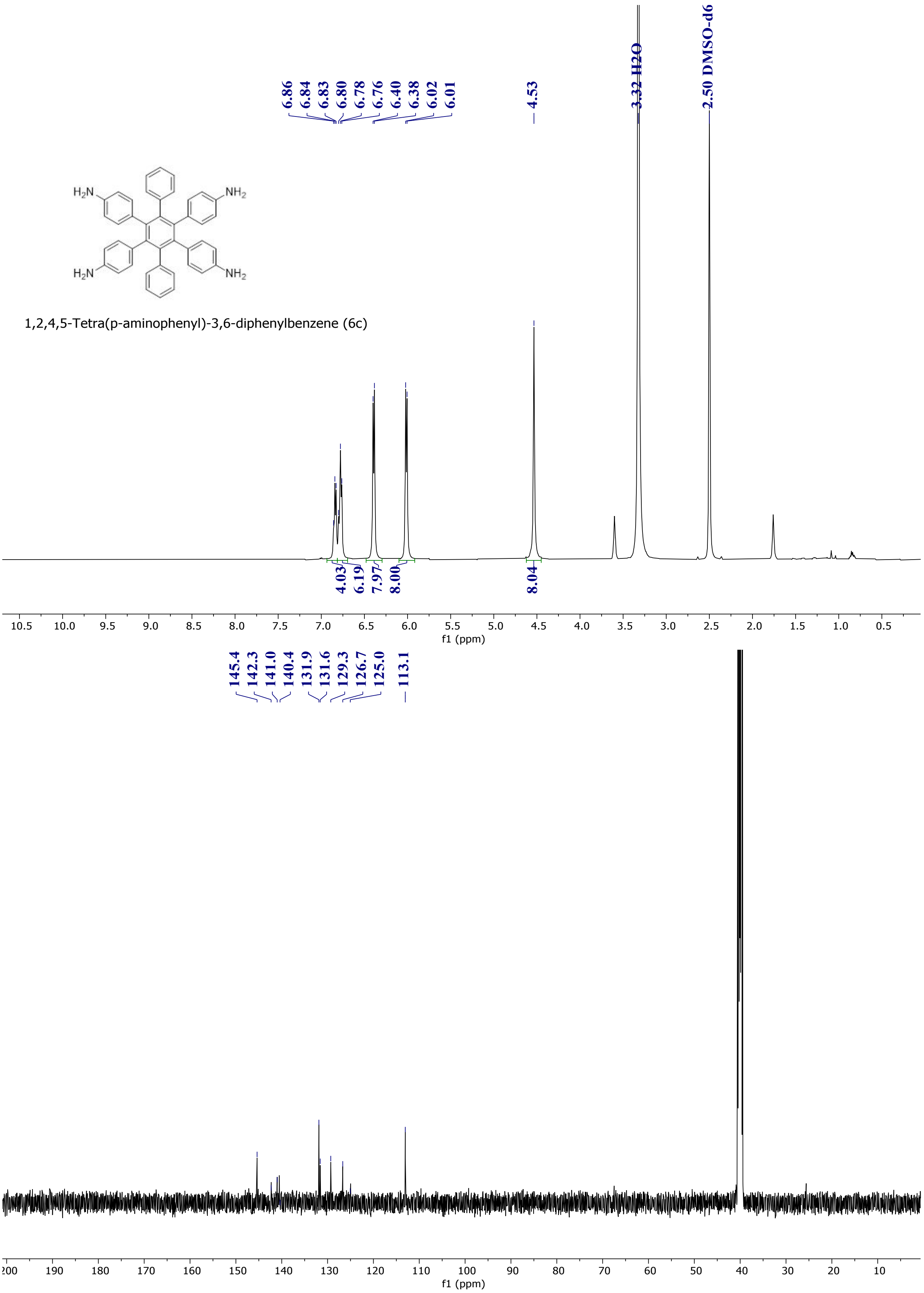

Figure S39. ^{1}H and ^{13}C NMR of 1,2,4,5-tetra(p-aminophenyl)-3,6-diphenylbenzene (6c)

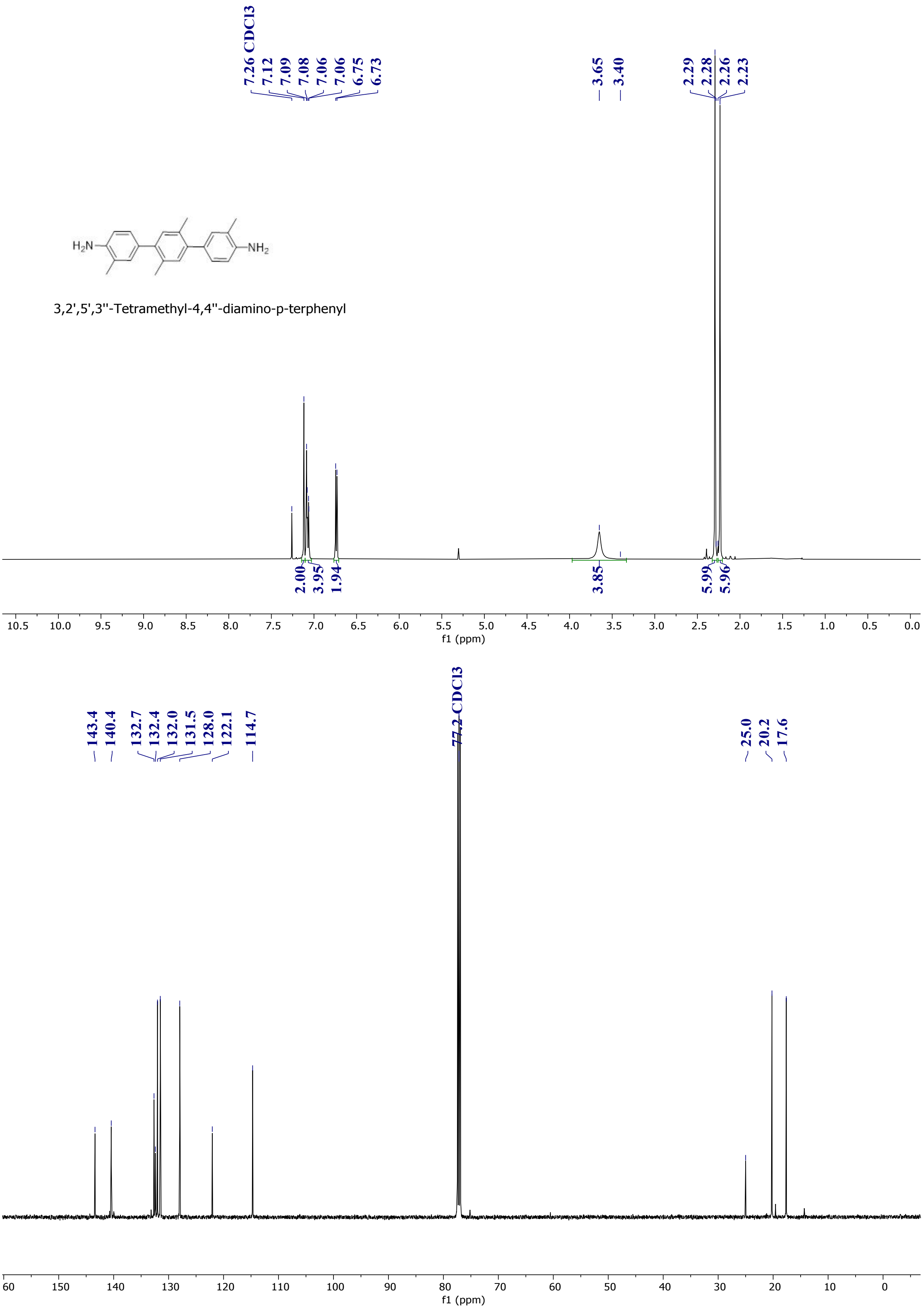

Figure S40. ^{1}H and ^{13}C NMR of 3,2',5',3''-tetramethyl-4,4''-diamino-p-terphenyl

References

(1) Myers, A. L.; Prausnitz, J. M. Thermodynamics of mixed-gas adsorption. *AIChE Journal* **1965**, *11* (1), 121-127.

(2) Lee, S.; Lee, J. H.; Kim, J. User-friendly graphical user interface software for ideal adsorbed solution theory calculations. *Korean Journal of Chemical Engineering* **2018**, *35* (1), 214-221.

(3) Pal, R.; Mukherjee, S.; Chandrasekhar, S.; Guru Row, T. N. Exploring Cyclopentadienone Antiaromaticity: Charge Density Studies of Various Tetracyclones. *The Journal of Physical Chemistry A* **2014**, *118* (19), 3479-3489.

(4) Potter, R. G.; Hughes, T. S. Predicting the UV−Vis Spectra of Tetraarylcyclopentadienones: Using DFT Molecular Orbital Energies to Model Electronic Transitions of Organic Materials. *The Journal of Organic Chemistry* **2008**, *73* (8), 2995-3004.

(5) Chmil, K.; Scherf, U. Poly(arylene)s containing structural units derived from the 1,2-diketo building block. *Die Makromolekulare Chemie* **1993**, *194* (5), 1377-1386.

(6) Shan, L.; Liu, D.; Li, H.; Xu, X.; Shan, B.; Xu, J.-B.; Miao, Q. Monolayer Field-Effect Transistors of Nonplanar Organic Semiconductors with Brickwork Arrangement. *Advanced Materials* **2015**, *27* (22), 3418-3423.

(7) Ruengsangtongkul, S.; Chaisan, N.; Thongsornkleeb, C.; Tummatorn, J.; Ruchirawat, S. Rate Enhancement in CAN-Promoted Pd(PPh3)2Cl2-Catalyzed Oxidative Cyclization: Synthesis of 2-Ketofuran-4-carboxylate Esters. *Organic Letters* **2019**, *21* (8), 2514-2517.

(8) Nakano, E.; Mutoh, K.; Kobayashi, Y.; Abe, J. Electrochemistry of Photochromic [2.2]Paracyclophane-Bridged Imidazole Dimers: Rational Understanding of the Electronic Structures. *The Journal of Physical Chemistry A* **2014**, *118* (12), 2288-2297.

(9) Mutoh, K.; Miyashita, N.; Arai, K.; Abe, J. Turn-On Mode Fluorescence Switch by Using Negative Photochromic Imidazole Dimer. *Journal of the American Chemical Society* **2019**, *141* (14), 5650-5654.

(10) Kuhn, R.; Zilliken, F.; Dury, K. Anästhesierende Wirkungen von 4.4 ′ -Diaminobenzil. *Naturwissenschaften* **1951**, *38* (1), 12-13.

(11) Thiemann, T.; Iniesta, J.; Walton, D. J. Thermal oxidation of tetracyclones (2,3,4,5-tetraarylcyclopentadienones). *Journal of Chemical Research* **2008**, *2008* (3), 173-180.

(12) Gagnon, E.; Maris, T.; Maly, K. E.; Wuest, J. D. The potential of intermolecular N center dot center dot center dot O interactions of nitro groups in crystal engineering, as revealed by structures of hexakis (4-nitrophenyl) benzene. *Tetrahedron* **2007**, *63* (28), 6603-6613.

(13) Sakaguchi, Y.; Harris, F. W. Synthesis and characterization of aromatic polyamides derived from new phenylated aromatic diamines. *Polymer journal* **1992**, *24* (10), 1147-1154.

(14) Rabbani, M. G.; Reich, T. E.; Kassab, R. M.; Jackson, K. T.; El-Kaderi, H. M. High CO 2 uptake and selectivity by triptycene-derived benzimidazole-linked polymers. *Chemical Communications* **2012**, *48* (8), 1141-1143.

(15) Antonangelo, A. R.; Hawkins, N.; Tocci, E.; Muzzi, C.; Fuoco, A.; Carta, M. Tröger's Base Network Polymers of Intrinsic Microporosity (TB-PIMs) with Tunable Pore Size for Heterogeneous Catalysis. *Journal of the American Chemical Society* **2022**, *144* (34), 15581-15594.

(16) Carta, M.; Malpass-Evans, R.; Croad, M.; Rogan, Y.; Lee, M.; Rose, I.; McKeown, N. B. The synthesis of microporous polymers using Tröger's base formation. *Polymer Chemistry* **2014**, *5* (18), 5267-5272,
